# Supplementary material for: Accessing the main-group metal formyl scaffold through CO-activation in beryllium hydride complexes
Source: Nat Commun. 2022 Jan 24;13:461. doi: 10.1038/s41467-022-28095-0 (PMC8786820; doi:10.1038/s41467-022-28095-0)
Supplement: Supplementary file 1 — Supplementary Information [file 41467_2022_28095_MOESM1_ESM.pdf]

Supplementary Information

Accessing the Main-Group Metal Formyl Scaffold through CO-Activation in Beryllium Hydride Complexes

Terrance J. Hadlington,*[,a] and Tibor Szilvási[b]

[a] Technische Universität München, Lichtenbergstraße 4, 85747 Garching, Germany

[b] Department of Chemical and Biological Engineering, University of Alabama, Tuscaloosa, AL 35487, United States

1. **Experimental methods and data…........................S2**
    a. Synthetic methods…………………………...……...S2
    b. NMR Spectra……………………….………...S5
    c. IR Spectra……………………………..…………S32
    d. Mass Spectra………………………..………..S36
2. **Single-crystal X-ray crystallographic details…..…S42**
3. **Power X-ray diffraction details……………………...S45**
4. **Computational methods and details……..……...S47**
5. **Supplementary references……………….……….S80**

1. Experimental methods and data

General considerations. All experiments and manipulations were carried out under a dry oxygen free argon atmosphere using standard Schlenk techniques, or in an MBraun inert atmosphere glovebox containing an atmosphere of high purity argon. THF and diethyl ether were dried by distillation over a sodium/benzophenone mixture and stored over activated 4Å mol sieves. C_6D_6 was dried and stored over a potassium mirror. All other solvents were dried over activated 4Å mol sieves and degassed prior to use. ^{PhiP}DippK,[1] ^{PhPh}DippK,[1] DippN(H)Li,[2] $^{i}Pr_2PCH_2Li$, $BeBr_2{\cdot}(Et_2O)_2$,[3] and BzK [4] were synthesized according to known literature procedures, which are given below (in all cases analytical data is in keeping with those reported in the given references). All other reagents were used as received. Solution-state NMR spectra were recorded on a Bruker AV 400 or 500 Spectrometer. Solid-state NMR spectra were recorded on a Bruker AV 300 Spectrometer. The ^{1}H and $^{13}C\{^{1}H\}$ NMR spectra were referenced to the residual solvent signals as internal standards. ^{9}Be, ^{11}B, ^{29}Si, and ^{31}P NMR spectra were externally calibrated with $BeBr_2{\cdot}(Et_2O)_2$, $BF_3.OEt_2$, $SiMe_4$, and H_3PO_4, respectively. LIFDI MS spectra were measured at a Waters Micromass LCT TOF mass spectrometer equipped with an LIFDI ion source (LIFDI 700) from Linden CMS GmbH. The samples were dissolved in dry toluene and filtered using a syringe filter under an inert atmosphere. The TOF setup was externally calibrated using polystyrene. ESI-MS was performed on an exactive plus orbitrap spectrometer from Thermo Fischer Scientific. Infrared spectra were measured with the Alpha FT IR from Bruker containing a platinum diamond ATR device. The compounds were measured as solids under inert conditions in a glovebox. For the ammonia activation experiments water free ammonia 5.0 was used.

CAUTION: *Beryllium, most notably as fine powders, and beryllium compounds are regarded as highly toxic and carcinogenic. A severe allergic reaction can also occur if inhaled, with the risk of causing chronic beryllium disease.*[5] *One should take care and use adequate safety measures (i.e. breathing apparatus, protective clothing, well-ventilated fume-hoods) for any manipulations involving beryllium and compounds containing this element.*[6]

$^{i}Pr_2PCH_2Li$. This compound was synthesized by modification of a reported procedure.[7] $^{i}Pr_2PCl$ (25 g, 163 mmol) was dissolved in Et_2O (50 mL), and cooled to -78 °C. MeLi (1.6M in Et_2O, 108 mL, 172 mmol) was added, and the reaction warmed to ambient temperature, leading to the formation of a colourless precipitate. After stirring at ambient temperature for 1 h, the reaction mixture was filtered, and subsequently cooled to 0 °C. The Et_2O was removed at this temperature under a dynamic vacuum, until a volume of ~20 mL is reached, yielding crude $^{i}Pr_2PMe$ of high purity, as ascertained by ^{31}P NMR spectroscopic analysis. $^{t}BuLi$ (1.7M in pentane, 115 mL, 196 mmol) is added to a separate flask, and all volatiles removed *in vacuo* leaving a colourless crystalline residue, which is redissolved in heptane (30 mL). This solution is cooled to -40 °C, and the crude $^{i}Pr_2PMe$ transferred into this flask. The reaction mixture is then heated under an Ar atmosphere for 24 h, without stirring, leading to formation of a pale orange solution and thick 'cake' of precipitated **$^{i}Pr_2PCH_2Li$**. Filtration, followed by washing with pentane (40 mL) and drying under vacuum for 1 h gives a free-flowing off-white powder, of adequate purity for subsequent use (15.6 g, 69 %).

^{1}**H NMR** (D_8-THF, 400 MHz, 298 K): δ = -1.24 (s, 2H, P-C*H*$_2$-Li), 0.76-0.94 (overlapping d, 12H, P-^{i}Pr-C*H*$_3$), 1.22 (sept of d, 2H, $^{2}J_{HP}$ = 1.6 Hz, $^{3}J_{HH}$ = 6.9 Hz, P-^{i}Pr-C*H*$_3$).

^{31}**P{^{1}H} NMR** (D_8-THF, 162 MHz, 298 K): δ = 21.3 (*P*-^{i}Pr$_2$).

N. B. The above spectral data deviate slightly from those reported previously.[8]

^{PhPh}DippNK. A yellow suspension of PPh_2CH_2Li·TMEDA (6.0 g, 18.6 mmol) in 100 mL hexane was cooled to -78 °C. The mixture was stirred vigorously and Ph_2SiCl_2 (3.9 mL, 18.6 mmol) was added. The mixture was allowed to warm to RT overnight. All volatiles were subsequently removed *in vacuo*, leaving a yellow oil. DippN(H)Li (3.4 g, 18.6 mmol) was added to the residue, and the flask cooled to -78 °C, followed by the addition of 50 mL THF. The mixture was stirred until dissolution of all solids was observed. The cold bath was then removed and the reaction allowed to warm to RT, leading to an orange solution. All volatiles were removed *in vacuo* and the oily residue extracted with 50 mL hexane, and filtered. The solvent was removed *in vacuo* and KH (0.9 g, 28.3 mmol) was added. After addition of 50 mL THF, gas started to evolve, and the mixture was vigorously stirred for a further 16 h. The dark brown suspension was filtered, and all volatiles were removed *in vacuo*. To the resulting oil 50 mL hexane was added, and the mixture treated in an ultrasonic bath causing the precipitation of copious pale brown powder, which was filtered and washed multiple times with hexane, and subsequently dried *in vacuo* to yield ^{PhPh}DippNK as an off-white powder (8.5 g, 14.3 mmol, 77%).

^{PhiP}DippNK. The procedure for the synthesis of **^{PhPh}DippNK** was followed, but using PPh_2CH_2Li·TMEDA (10 g, 31 mmol), DippN(H)Li (5.5 g, 31 mmol), and, in place of Ph_2SiCl_2, $Pr^i{}_2SiCl_2$ (5.6 mL, 31 mmol). The product **^{PhiP}DippNK** was isolated as a pale-yellow powder (11.8 g, 72 %).

DippN(H)Li. A solution of $DippNH_2$ (40 mL, 0.26 mol) in hexane (250 mL) was cooled to -78 °C with rapid stirring, and ^{n}BuLi (107 mL, 2.5 M in hexane, 0.27 mol) added dropwise over 10 min leading to a white slurry. The reaction was continually stirred whilst warming to ambient temperature, and subsequently stirred for a further 1 hr. The white solid was allowed to settle for 1 hr, and the supernatant removed by filtration. The solid was washed (2 x 70 mL hexane), and dried under dynamic vacuum to afford a white free-flowing powder (39.2 g, 82 %).

$BeBr_2$·$(Et_2O)_2$. Beryllium pieces (0.3 g, 33.3 mmol) were suspended in diethyl ether (60 mL) in a flask fitted with a reflux condenser, and the suspension cooled to -40 °C. Bromine (1.7 mL, 33.3 mmol) was slowly added to the stirring suspension, and the reaction then brought to 0 °C. The reaction was held at the temperature for 30 min, and subsequently warmed to ambient temperature, at which stage a vigorous reflux is initiated. Once the reflux does not maintain itself, the reaction is heated under reflux for a further two hours, and cooled to ambient temperature. All volatiles are removed *in vacuo*, and the residue extracted in toluene (50 mL), filtered, and cooled to -40 °C overnight to afford a large crop of grey-white crystalline solid (8.9 g, 85%).

BzK. KO^{t}Bu (10 g, 89.3 mmol) is suspended in toluene (100 mL) and cooled to -78 °C with rapid stirring. ^{n}BuLi (37.5 mL, 2.5 M, 93.8 mmol) is added dropwise over 10 min, leading to a deep orange solution with coloured precipitate. The reaction is warmed to ambient temperature and stirred for 1 hr, leading to the formation of copious deep red solid, and a near colourless supernatant. The solid is allowed to settle, and the solution removed by filtration. The solid is washed (1 x 40 mL toluene, 3 x 100 mL hexane), and dried under vacuum to give an extremely air and moisture sensitive, free flowing red powder.

NMR spectra

^{iPPh}DippNK:

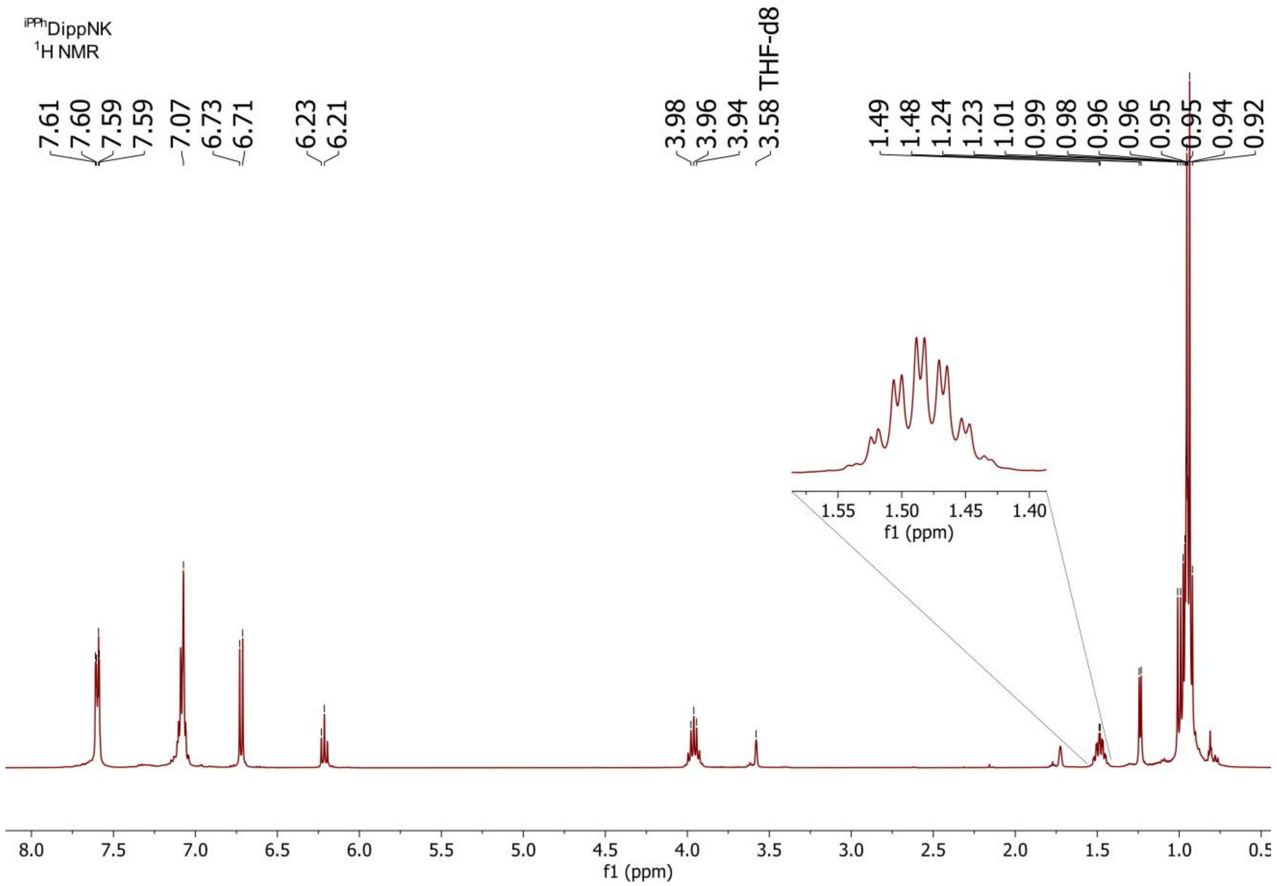

Supplementary Figure 1. ^{1}H NMR spectrum of ^{iPPh}DippK in D_8-THF at ambient temperature.

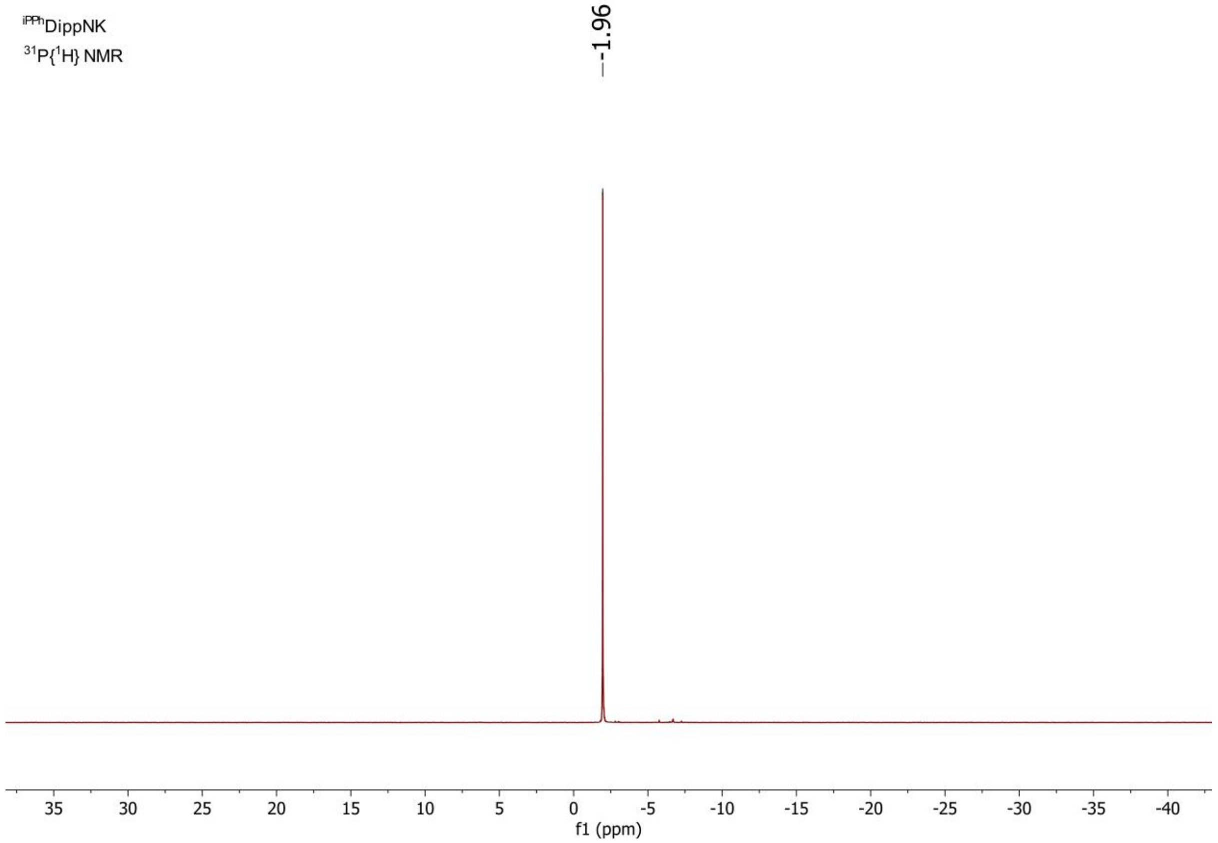

Supplementary Figure 2. ^{31}P{^{1}H} NMR spectrum of ^{iPPh}DippK in D_8-THF at ambient temperature.

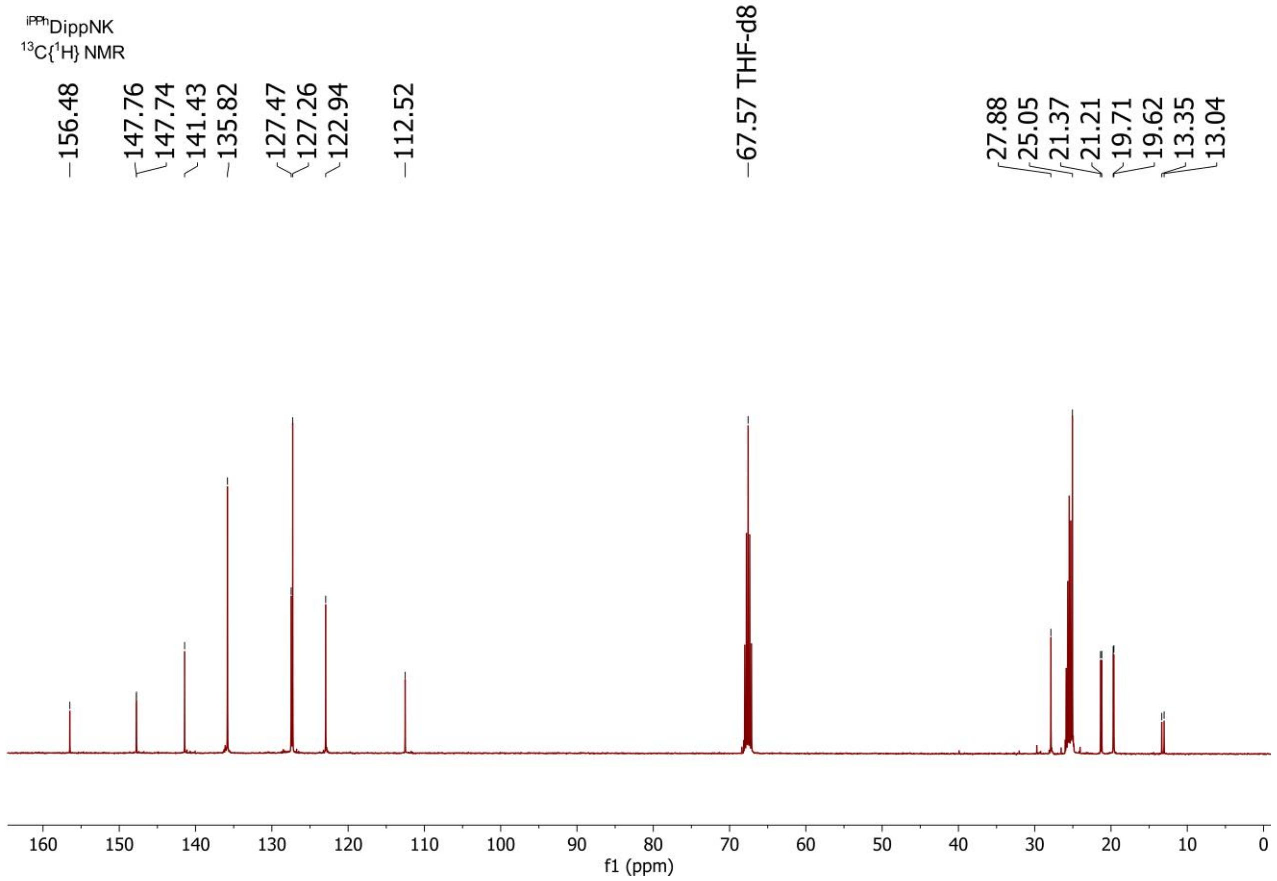

Supplementary Figure 3. $^{13}C\{^{1}H\}$ NMR spectrum of ^{iPPh}DippK in D_8-THF at ambient temperature.

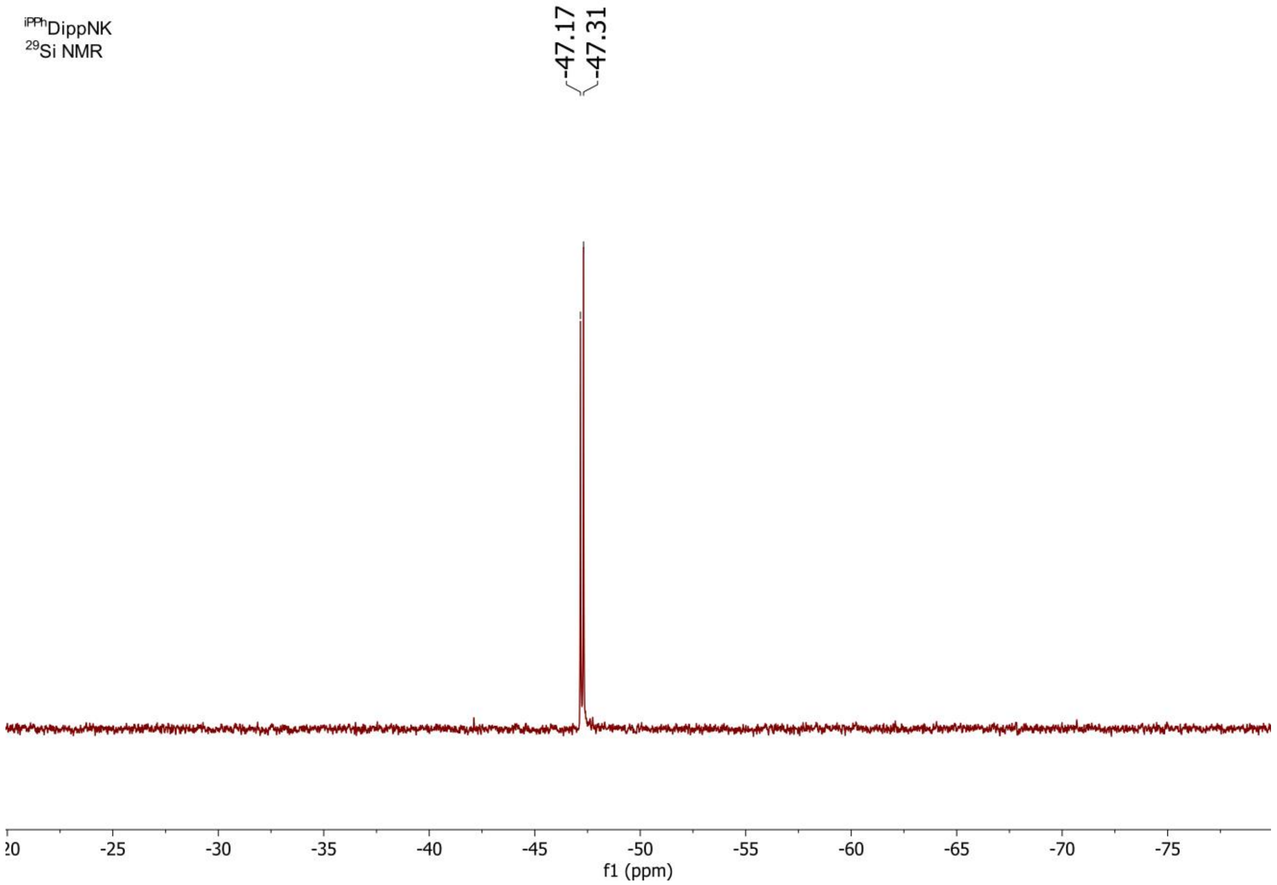

Supplementary Figure 4. ^{29}Si NMR spectrum of ^{iPPh}DippK in D_8-THF at ambient temperature.

PhiPDippBeBr, 1:

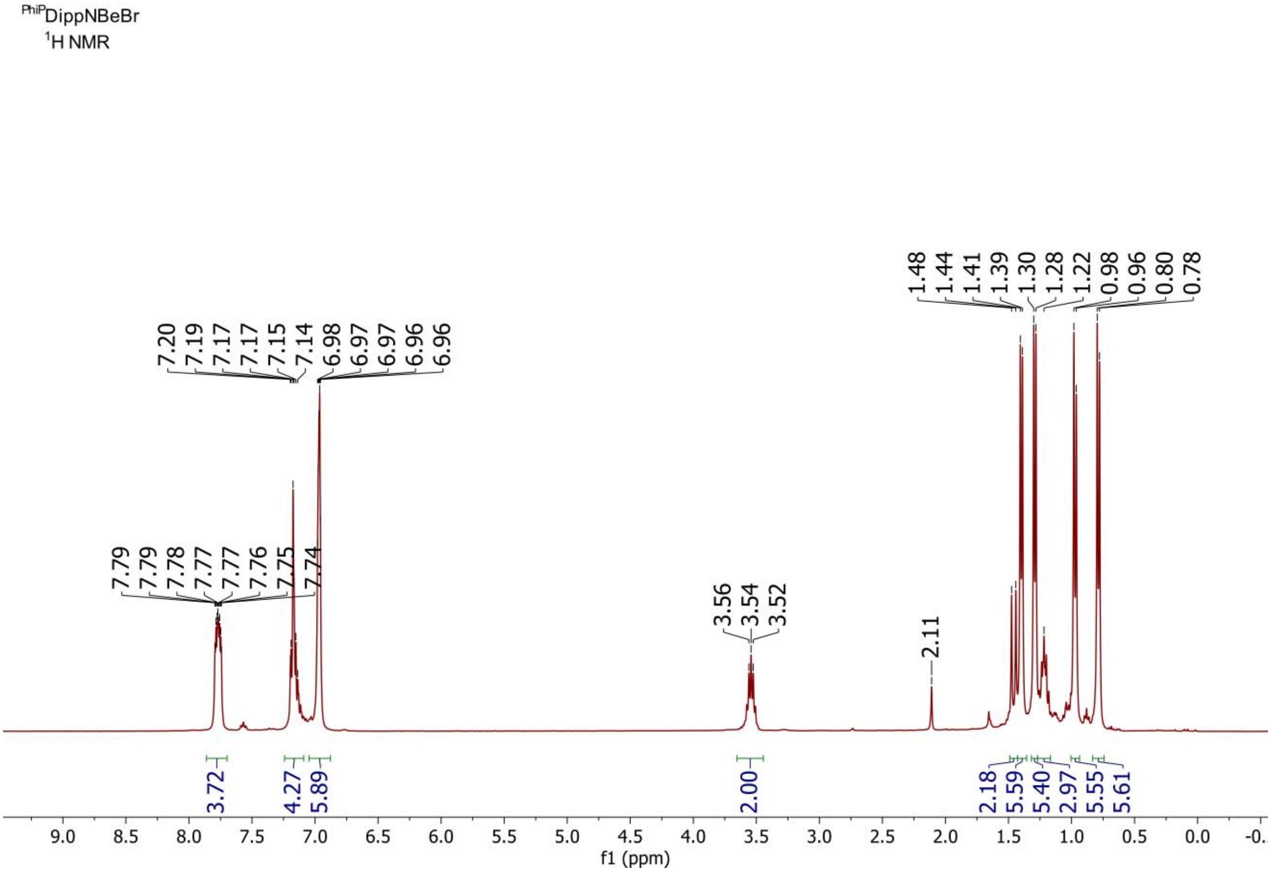

Supplementary Figure 5. ^{1}H NMR spectrum of PhiPDippBeBr in C_6D_6 at ambient temperature.

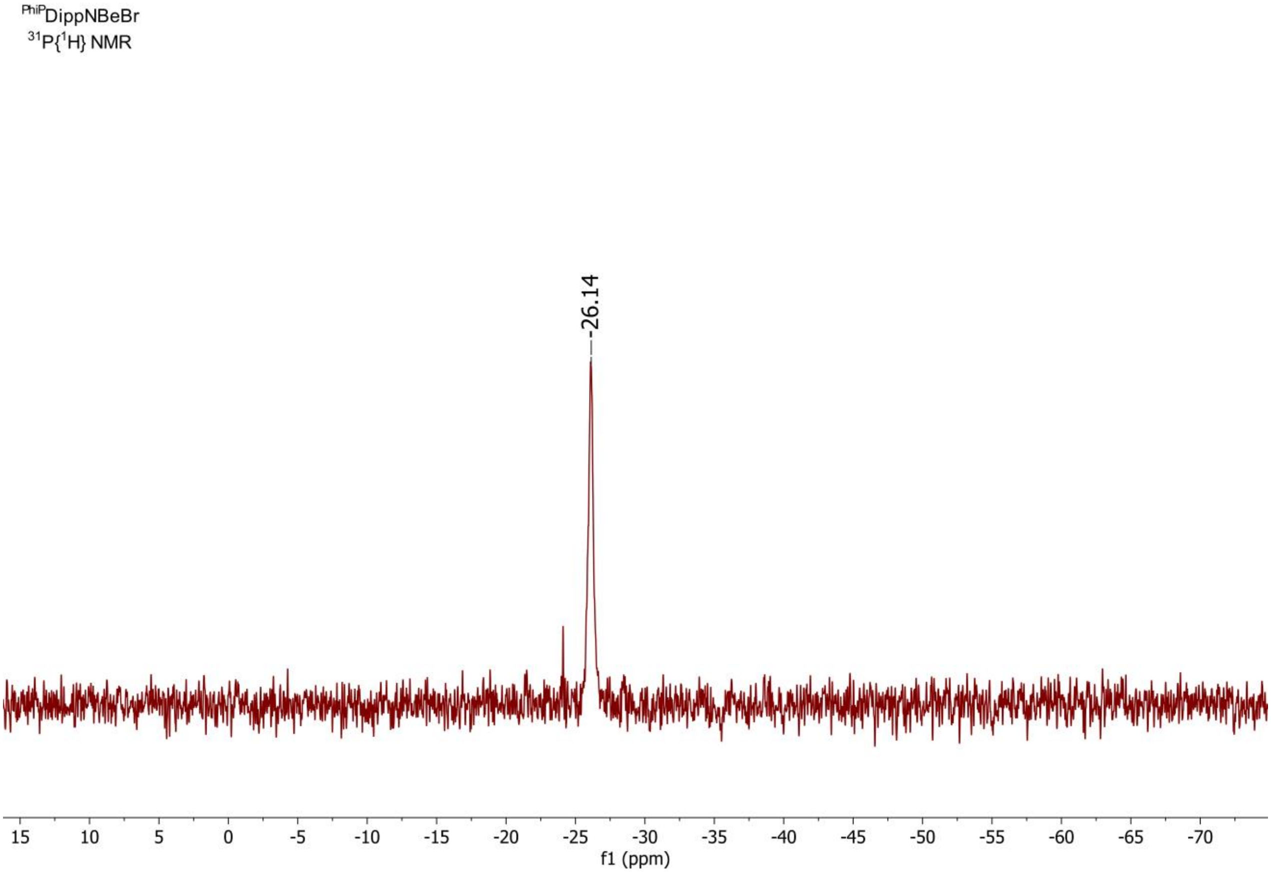

Supplementary Figure 6. $^{31}P\{^{1}H\}$ NMR spectrum of PhiPDippBeBr in C_6D_6 at ambient temperature.

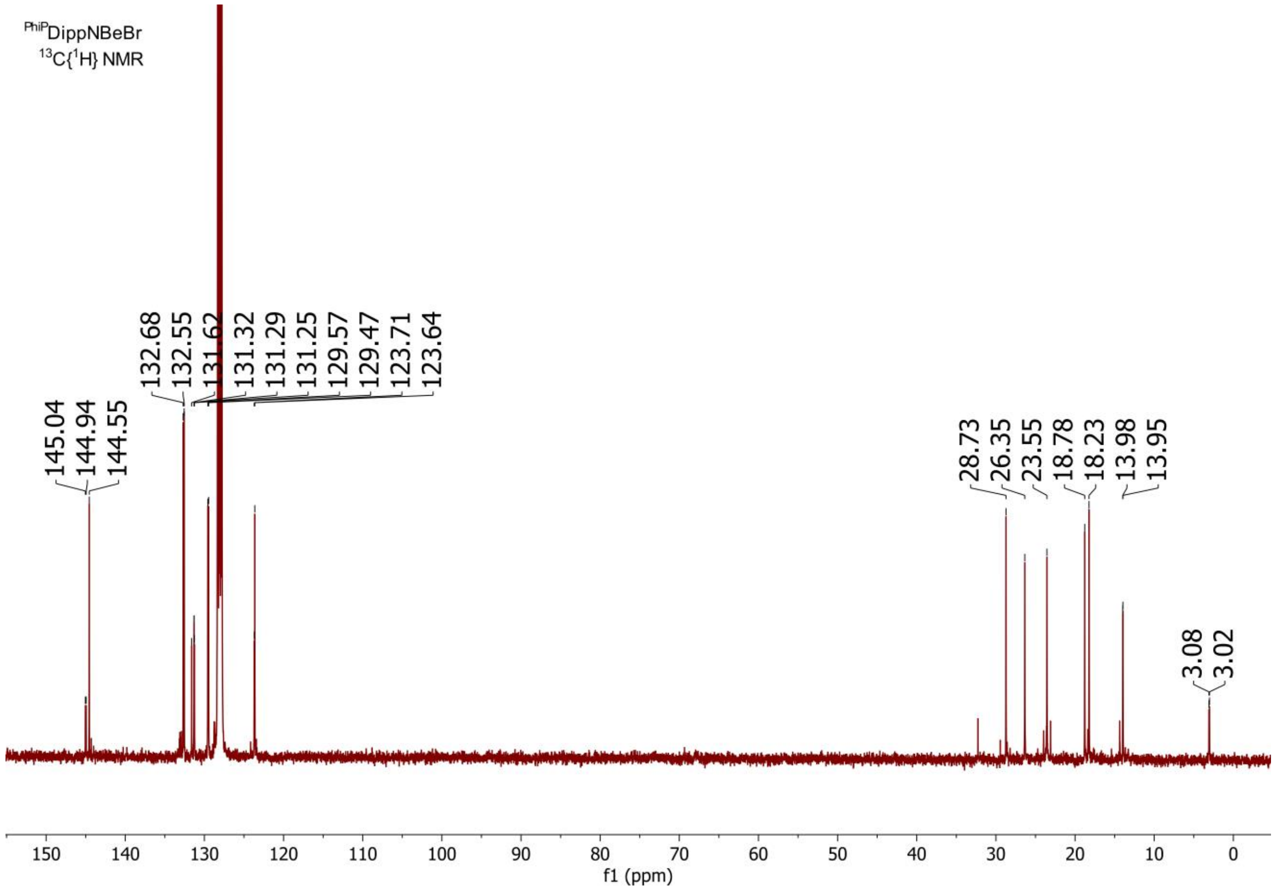

Supplementary Figure 7. $^{13}C\{^1H\}$ NMR spectrum of ^{PhiP}DippBeBr in C_6D_6 at ambient temperature.

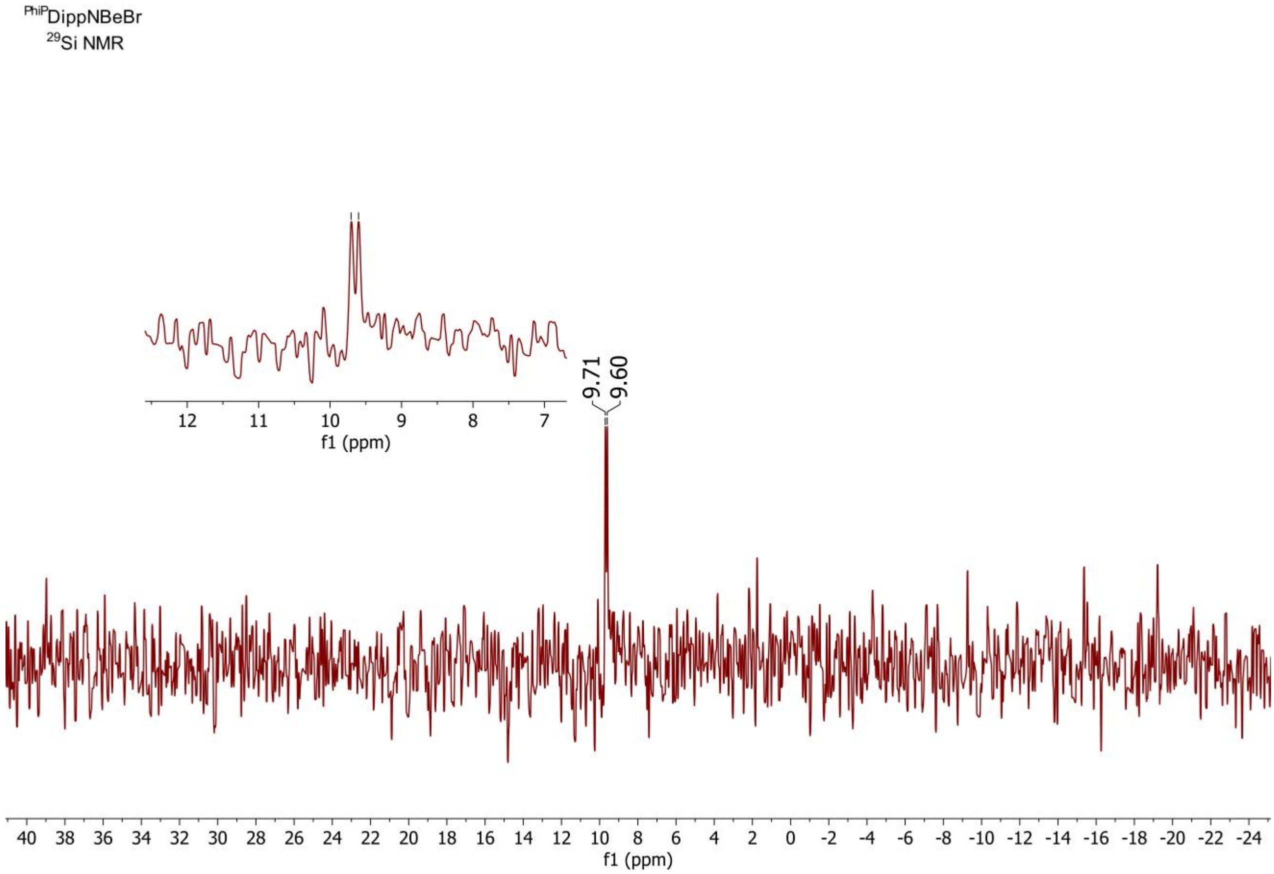

Supplementary Figure 8. ^{29}Si NMR spectrum of ^{PhiP}DippBeBr in C_6D_6 at ambient temperature.

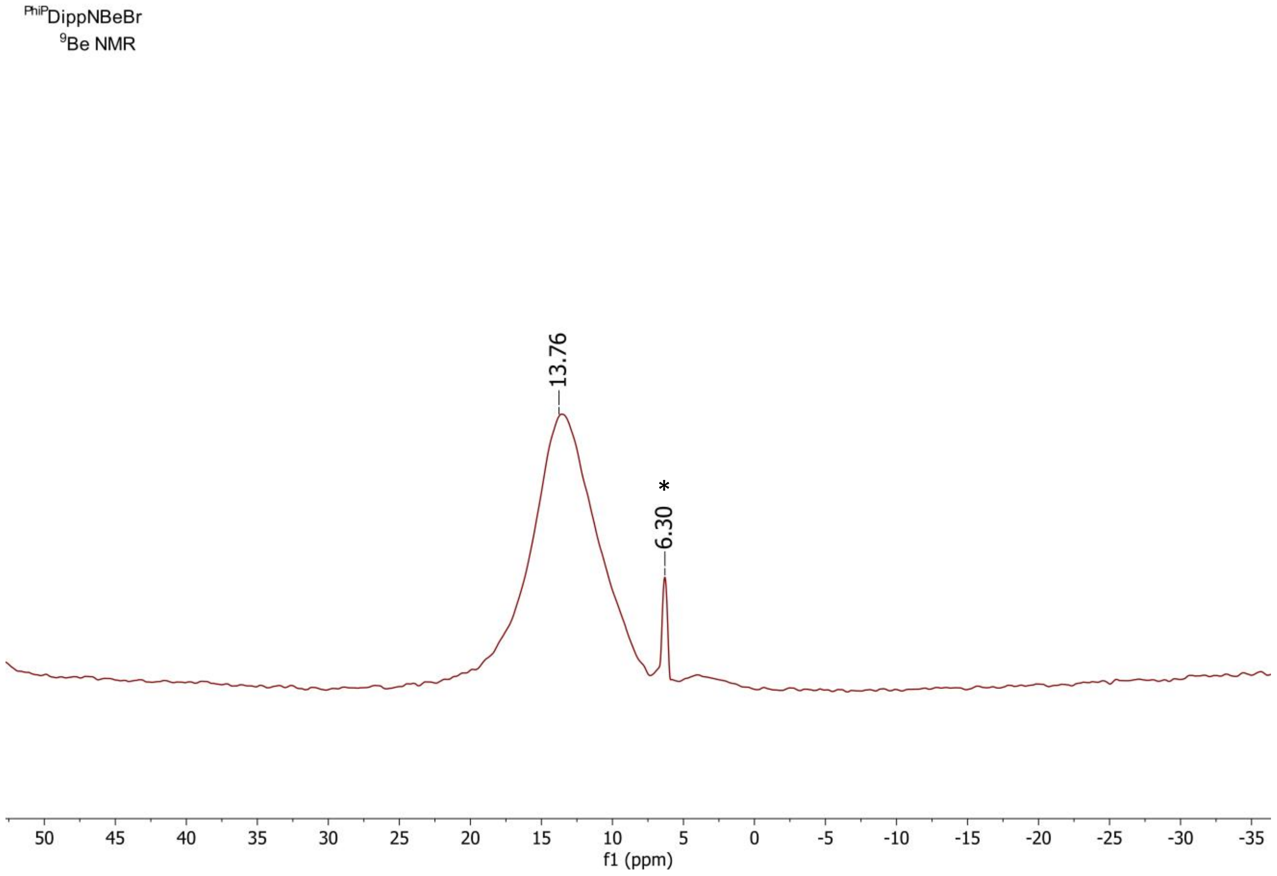

Supplementary Figure 9. ^{9}Be NMR spectrum of ^{PhiP}DippBeBr in C_6D_6 at ambient temperature. * *Denotes unidentified impurity.*

^{PhiP}DippBeBz, 4:

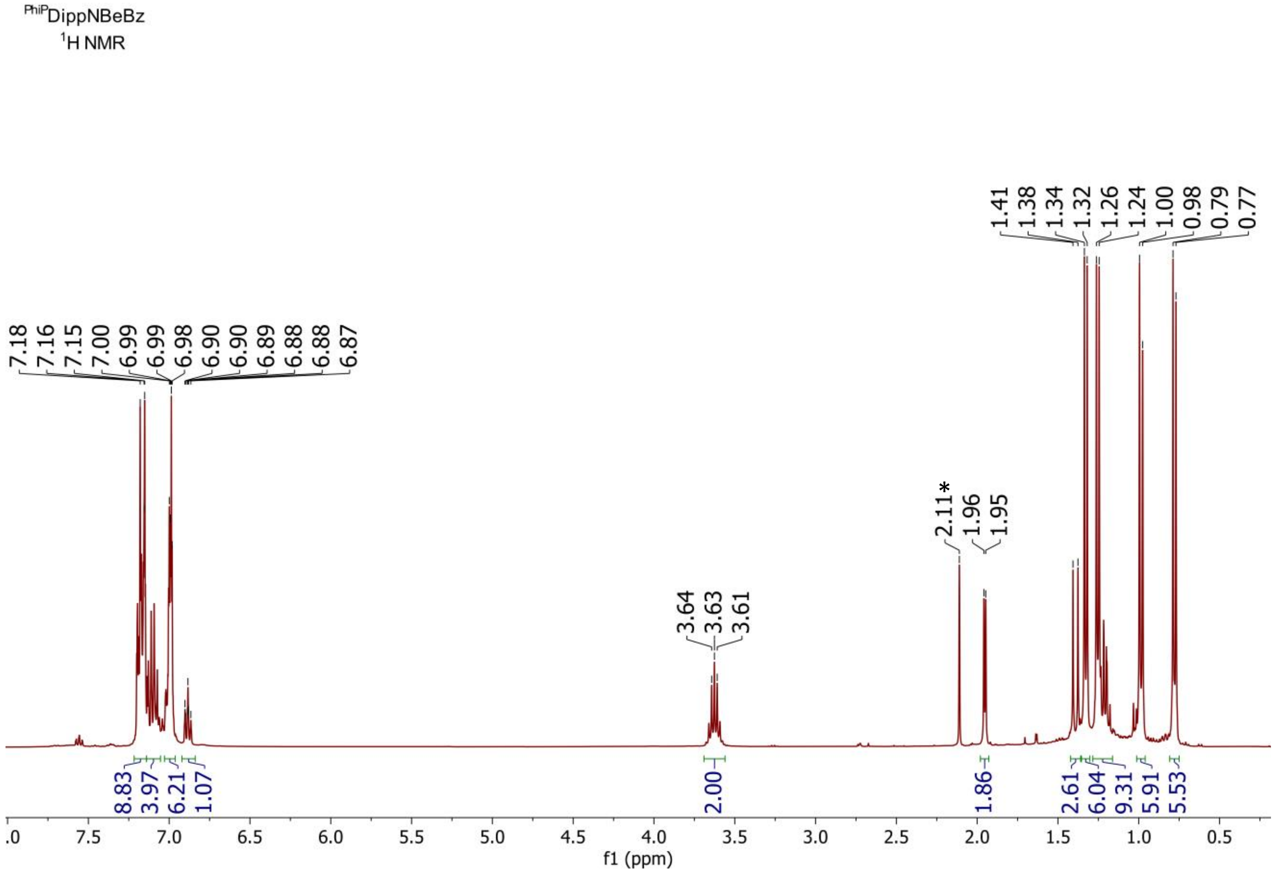

Supplementary Figure 10. ^{1}H NMR spectrum of ^{PhiP}DippBeBz in C_6D_6 at ambient temperature. * *Denotes residual toluene.*

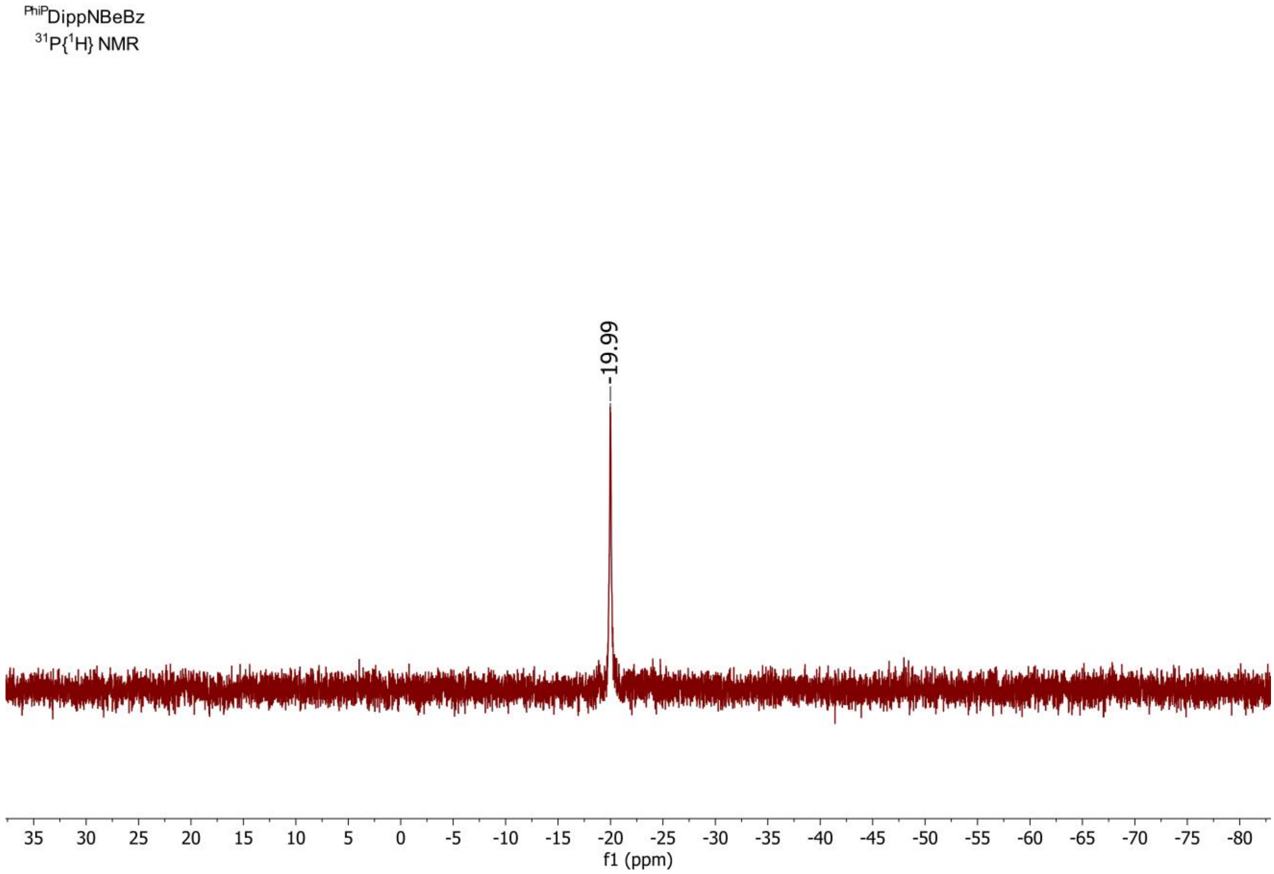

Supplementary Figure 11. $^{31}P\{^{1}H\}$ NMR spectrum of ^{PhiP}DippBeBz in C_6D_6 at ambient temperature.

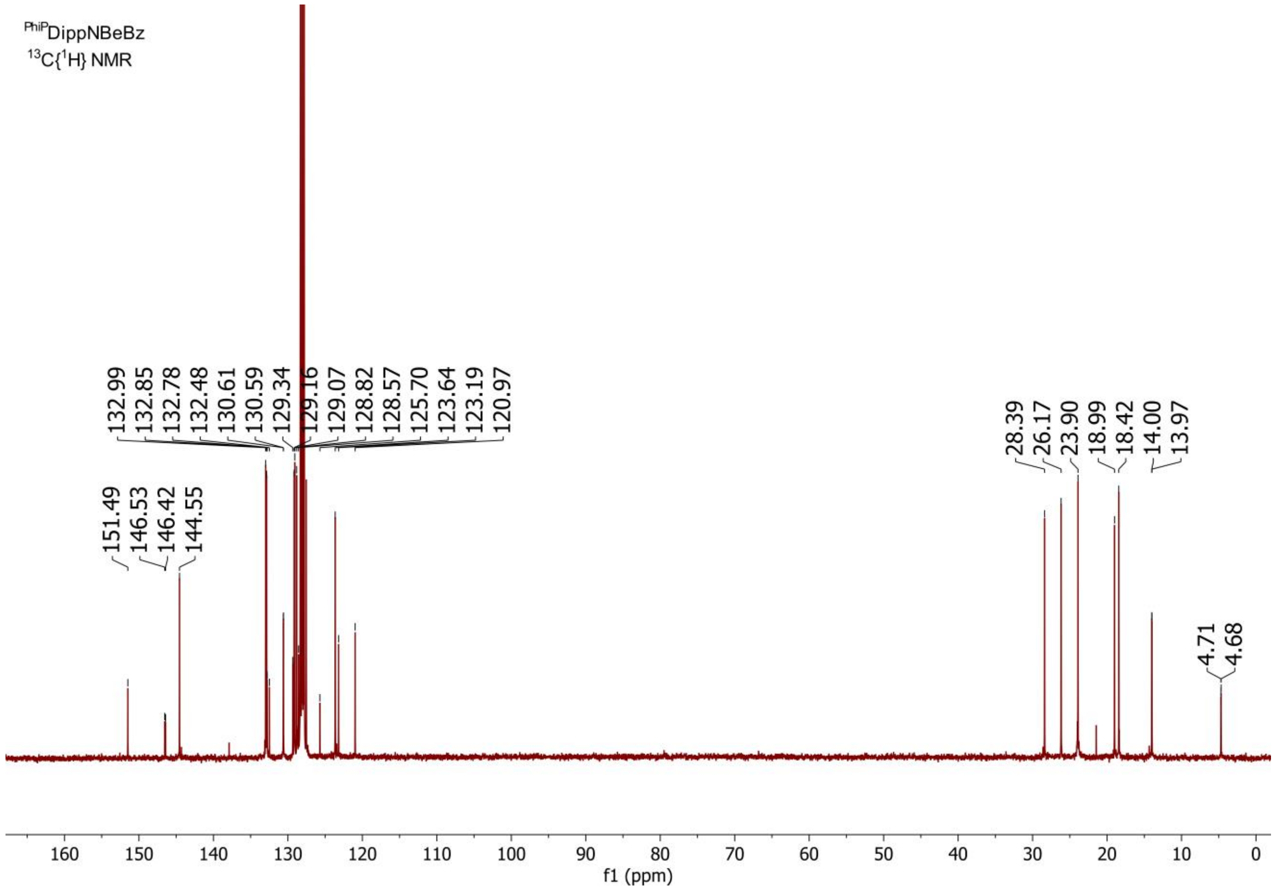

Supplementary Figure 12. $^{13}C\{^{1}H\}$ NMR spectrum of ^{PhiP}DippBeBz in C_6D_6 at ambient temperature.

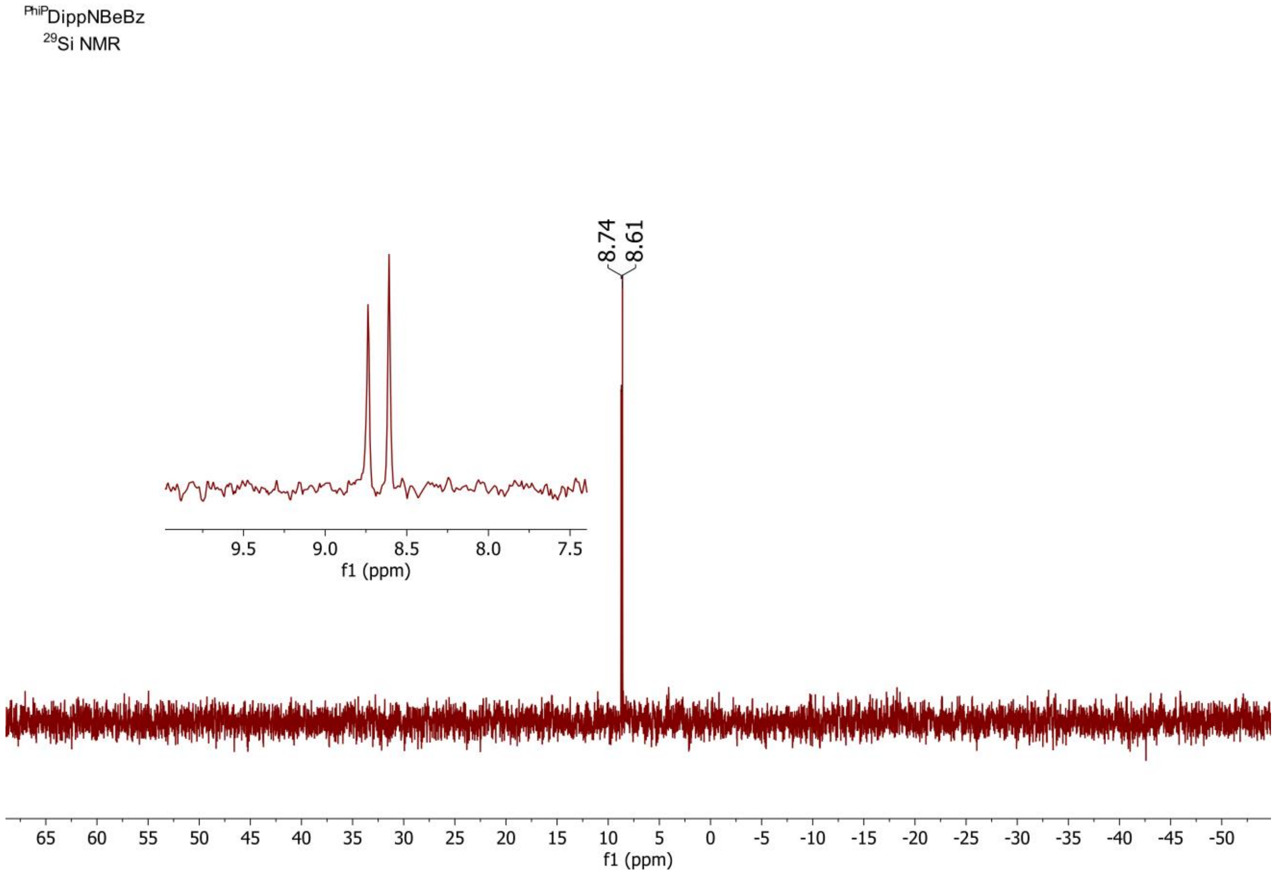

Supplementary Figure 13. ^{29}Si NMR spectrum of ^{PhiP}DippBeBz in C_6D_6 at ambient temperature.

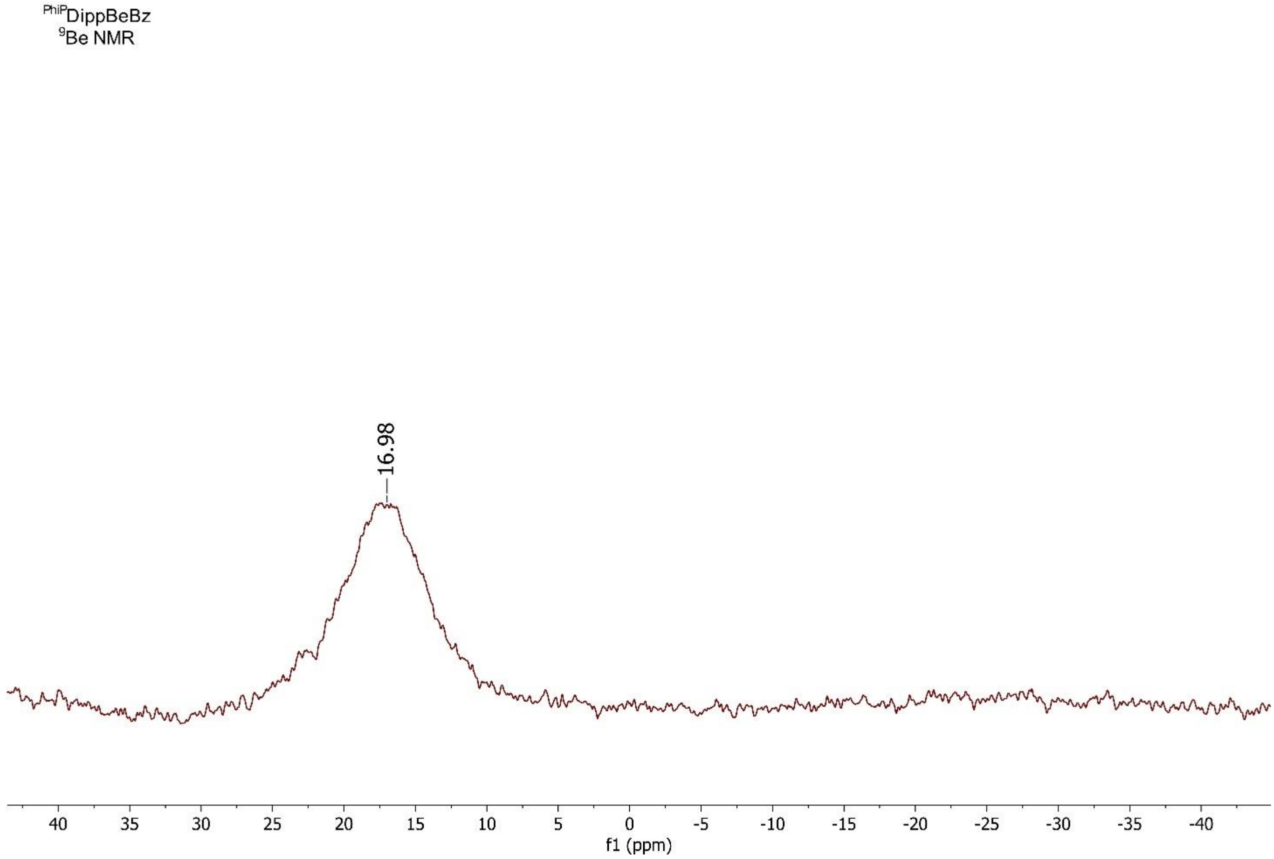

Supplementary Figure 14. ^{9}Be NMR spectrum of ^{PhiP}DippBeBz in C_6D_6 at ambient temperature.

^{PhiP}DippBe(pin)BH$_2$:

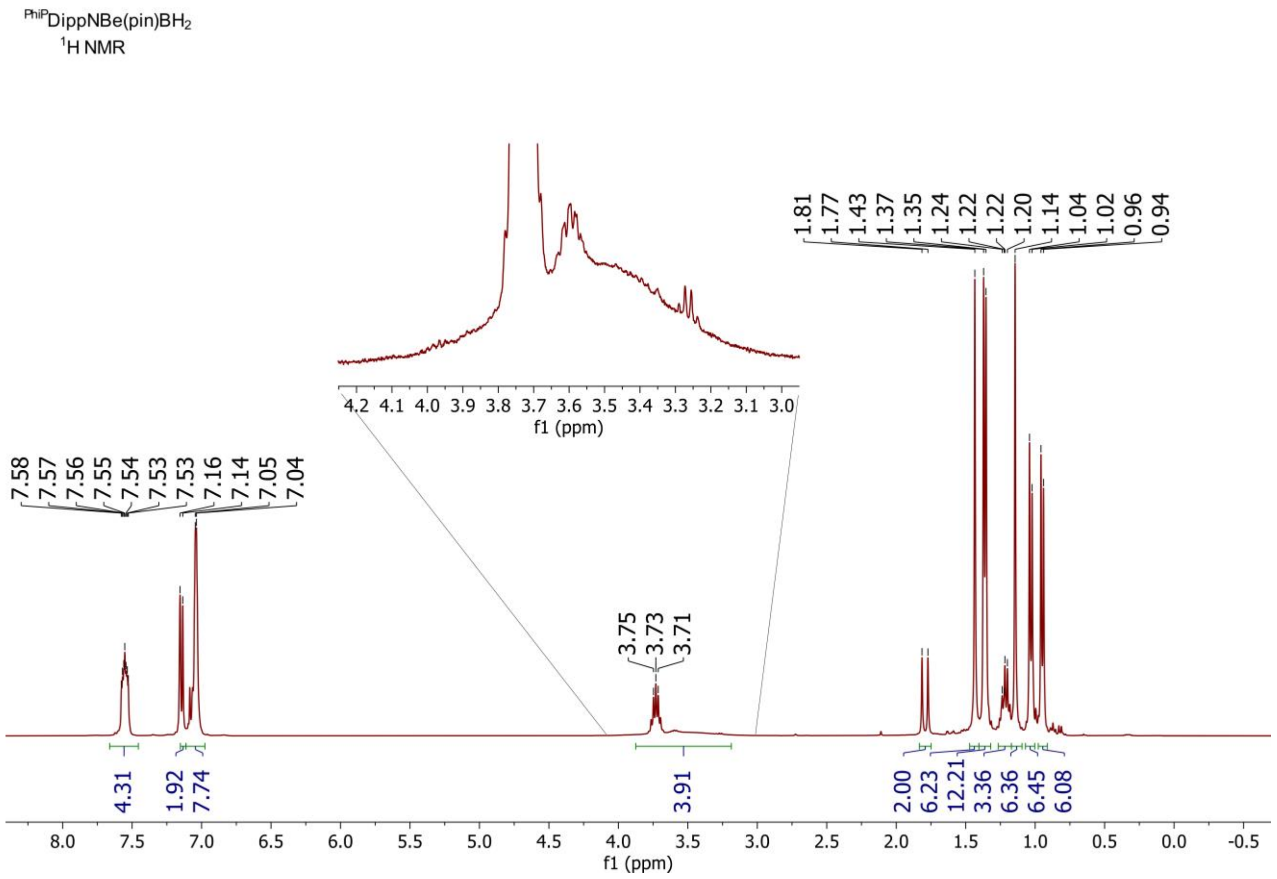

Supplementary Figure 15. ^{1}H NMR spectrum of ^{PhiP}DippBe(pin)BH$_2$ in C_6D_6 at ambient temperature.

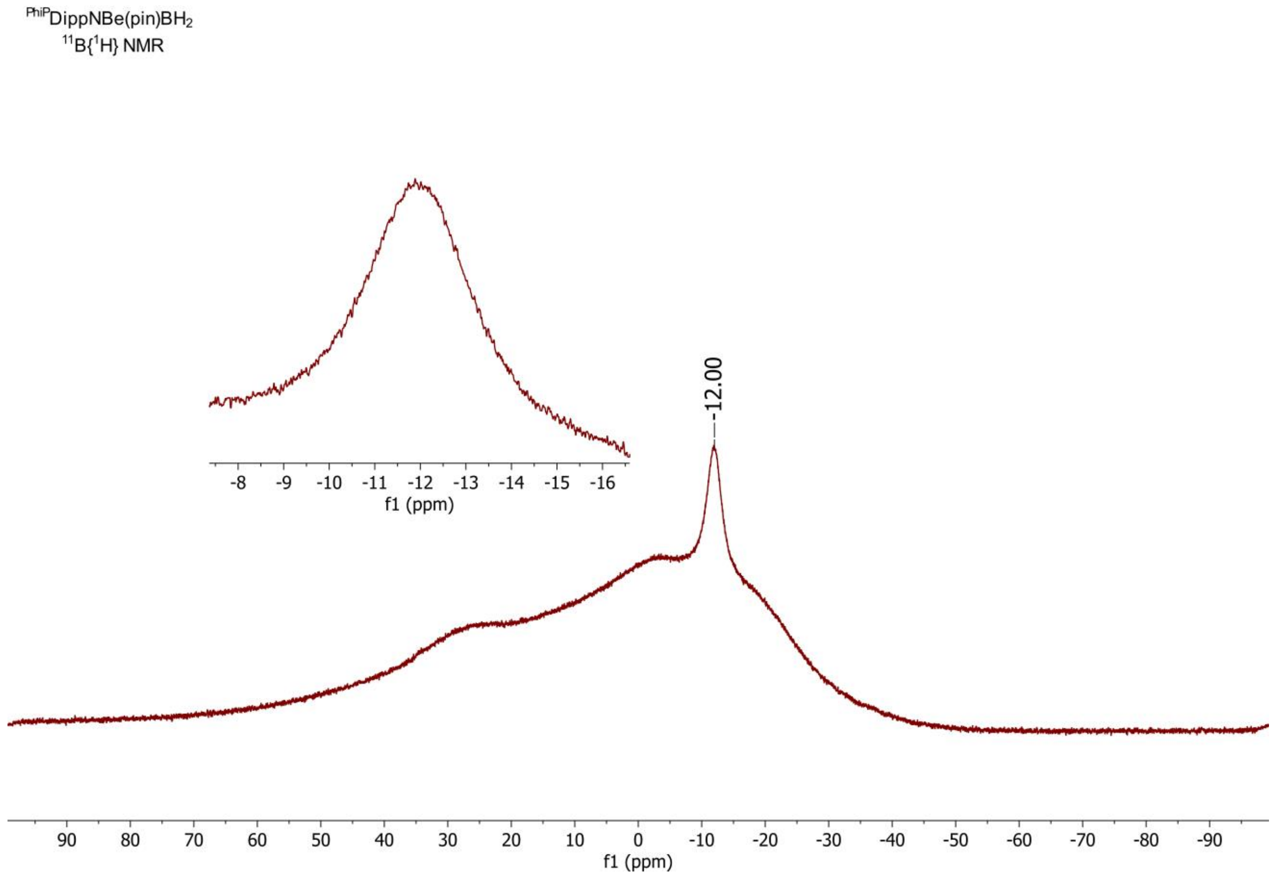

Supplementary Figure 16. ^{11}B{^{1}H} NMR spectrum of ^{PhiP}DippBe(pin)BH$_2$ in C_6D_6 at ambient temperature.

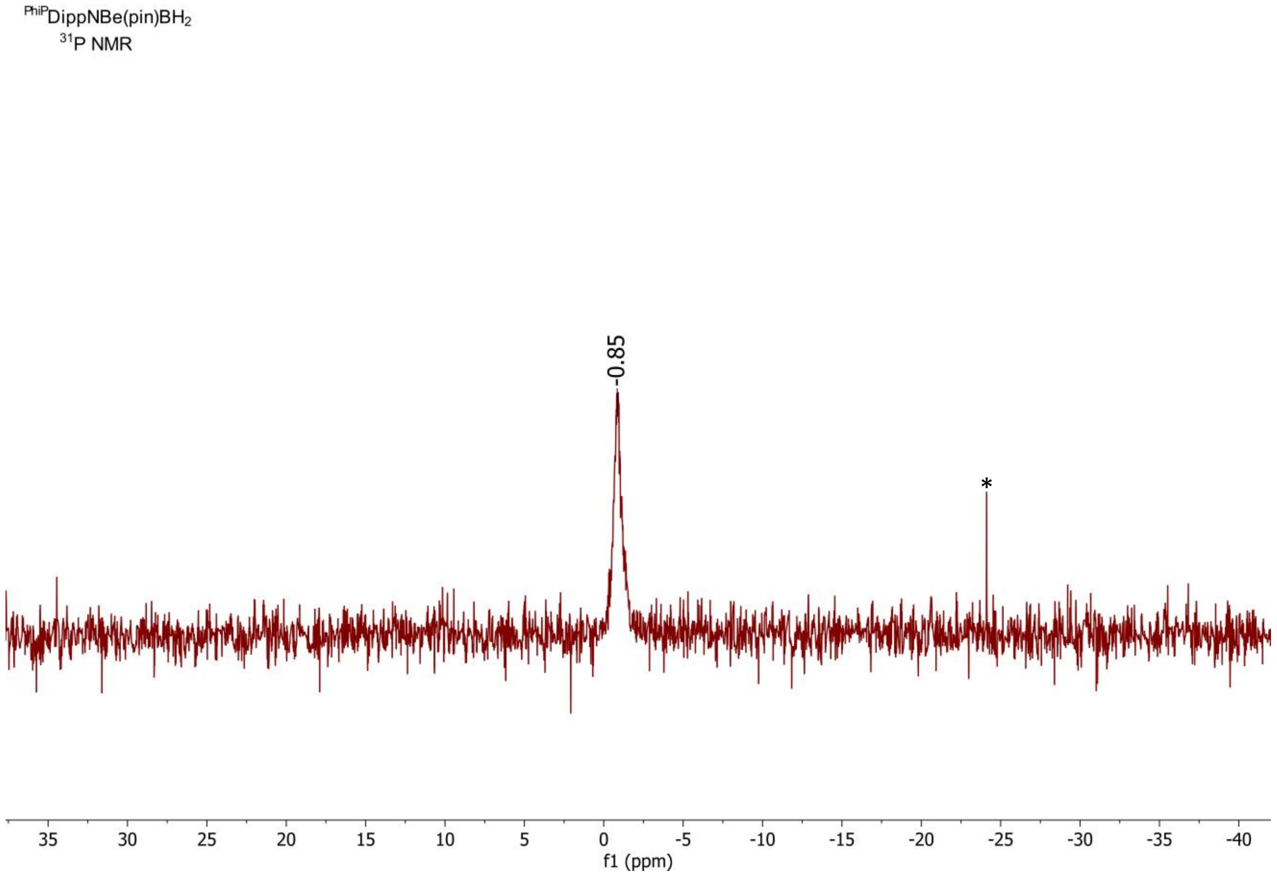

Supplementary Figure 17. $^{31}P\{^{1}H\}$ NMR spectrum of ^{PhiP}DippBe(pin)BH$_2$ in C_6D_6 at ambient temperature. * *Denotes a small amount of protonated ligand,* ^{PhiP}*DippNH.*

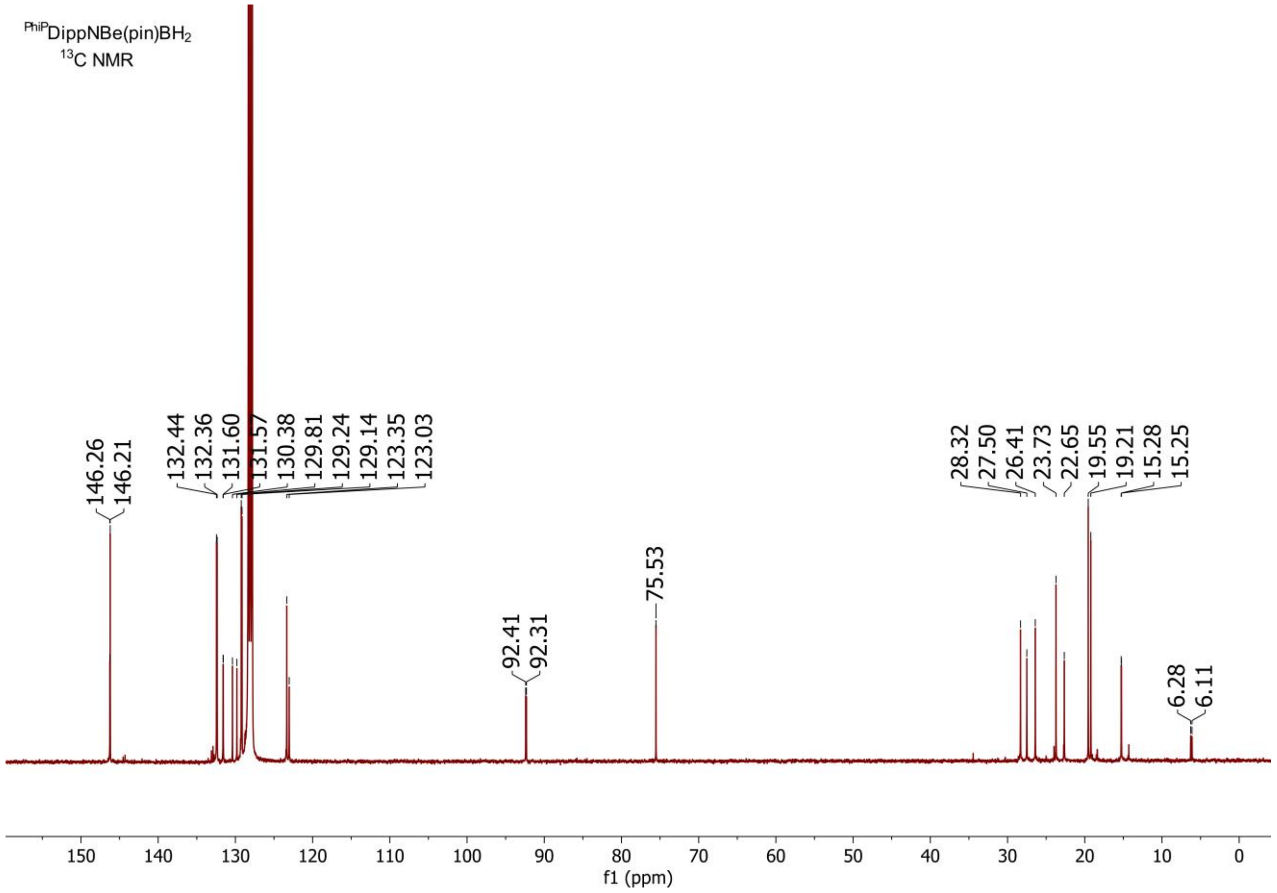

Supplementary Figure 18. $^{31}C\{^{1}H\}$ NMR spectrum of ^{PhiP}DippBe(pin)BH$_2$ in C_6D_6 at ambient temperature.

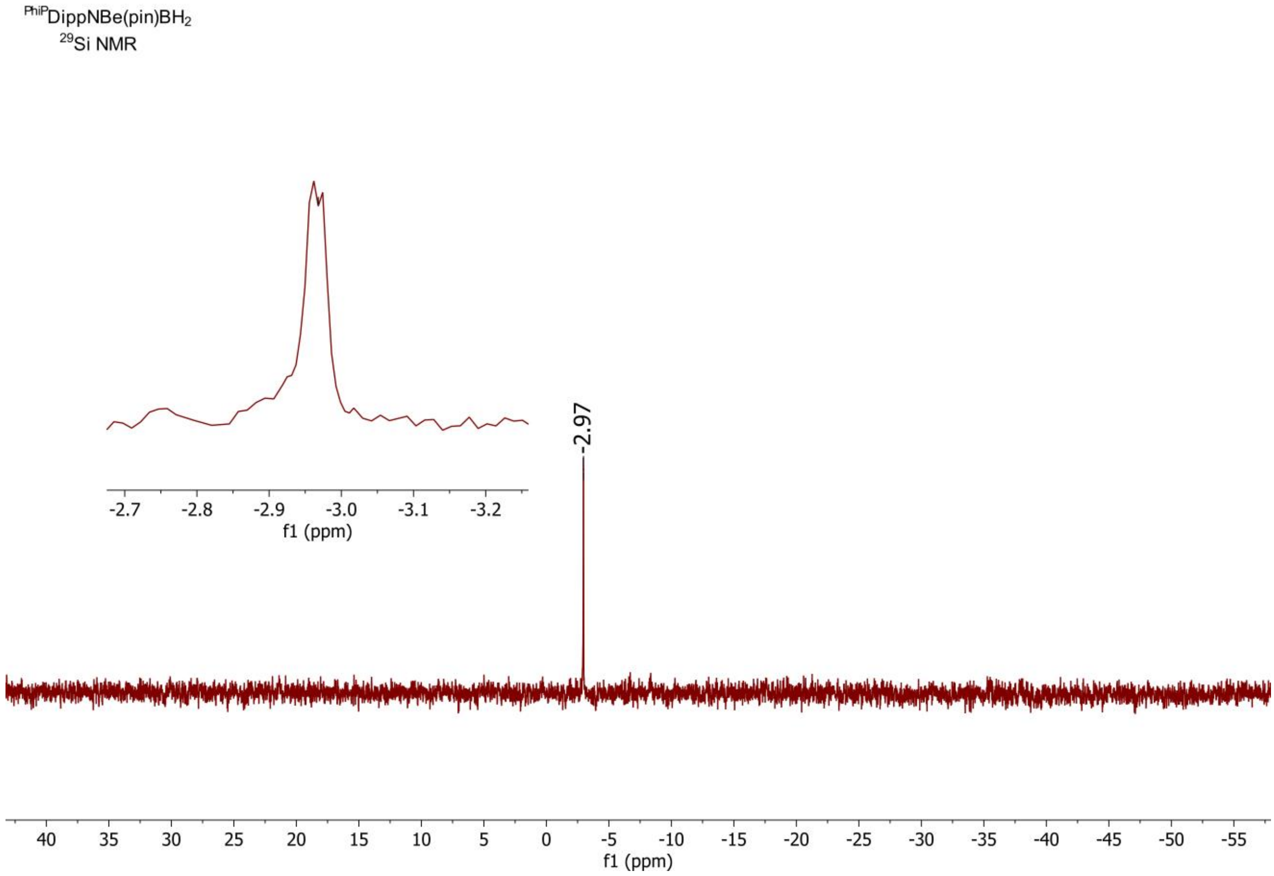

Supplementary Figure 19. ^{29}Si NMR spectrum of ^{PhiP}DippBe(pin)BH_2 in C_6D_6 at ambient temperature.

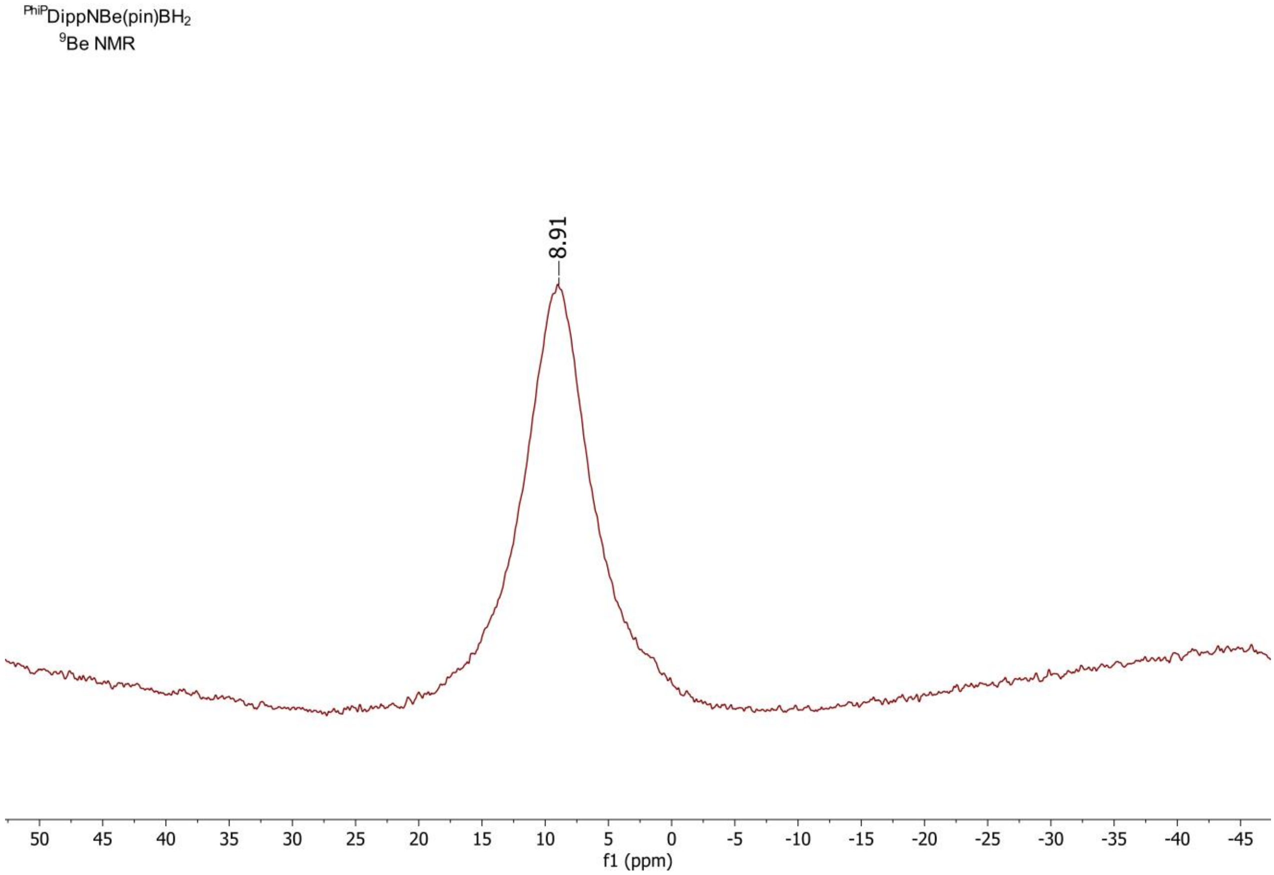

Supplementary Figure 20. ^{9}Be NMR spectrum of ^{PhiP}DippBe(pin)BH_2 in C_6D_6 at ambient temperature.

[^{PhiP}DippBe-μ$_2$-H]$_2$, 6.

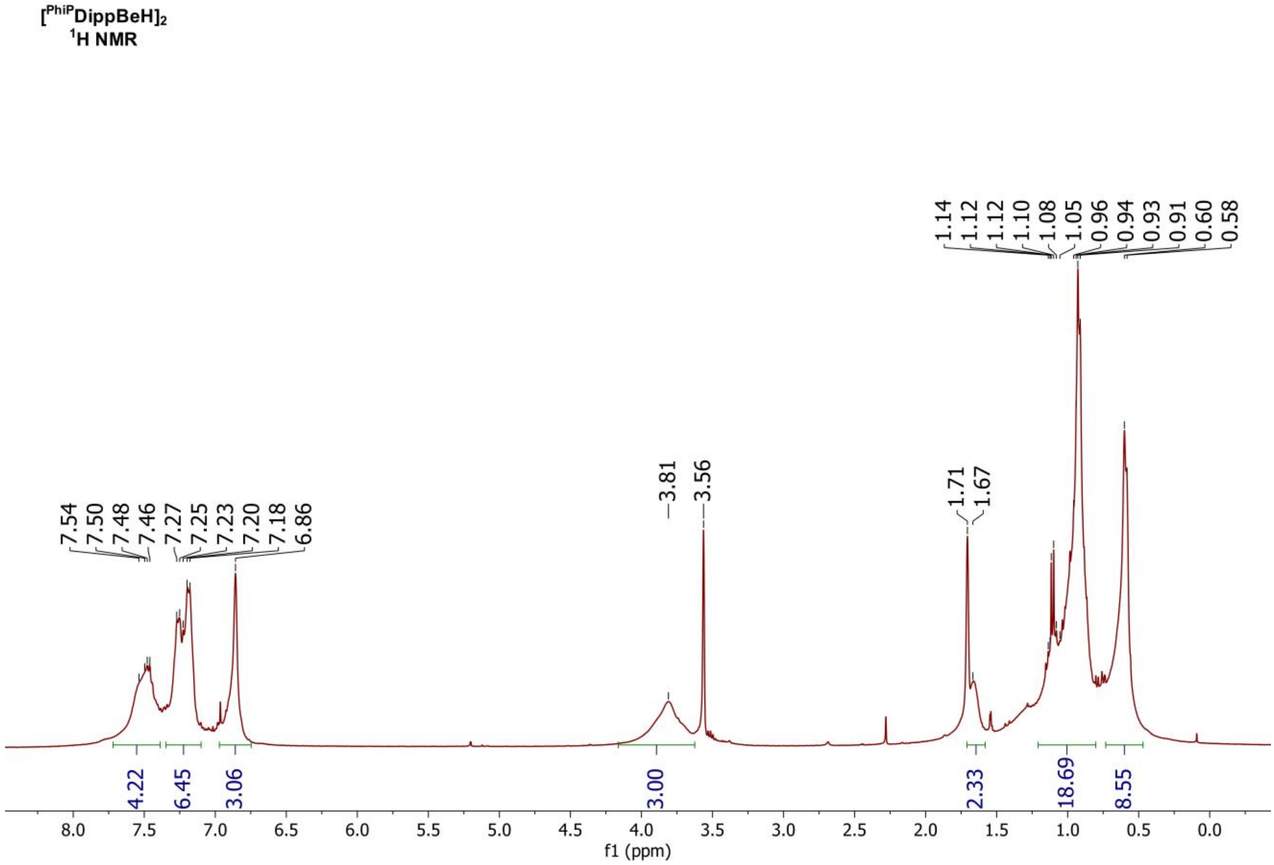

Supplementary Figure 21. ^{1}H NMR spectrum of [^{PhiP}DippBe-μ$_2$-H]$_2$ in D$_8$-THF at 338 K.

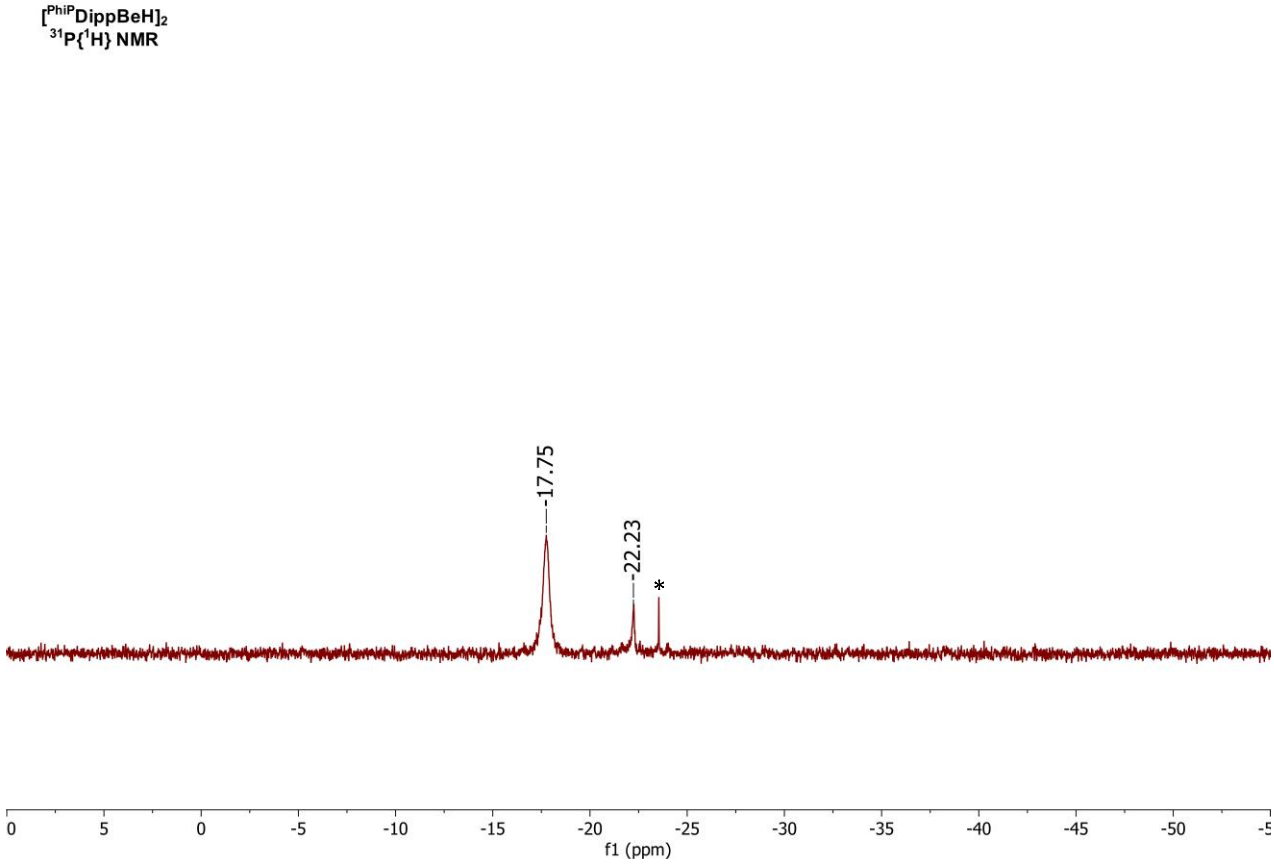

Supplementary Figure 22. ^{31}P{^{1}H} NMR spectrum of [^{PhiP}DippBe-μ$_2$-H]$_2$ in D$_8$-THF at 338 K. * *Denotes a small amount of protonated ligand, ^{PhiP}DippNH.*

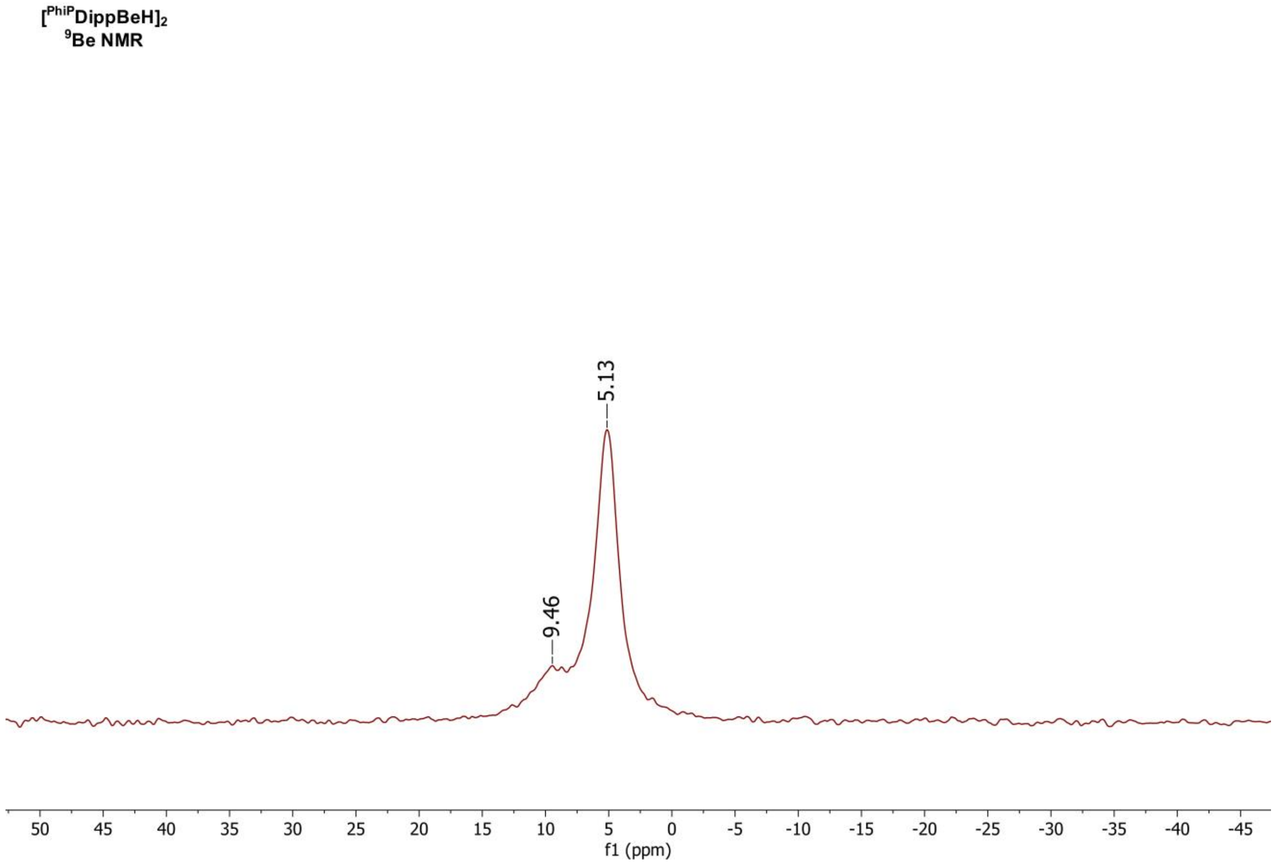

Supplementary Figure 23. ^{9}Be NMR spectrum of [^{PhiP}DippBe-μ$_2$-H]$_2$ in D$_8$-THF at 338 K.

[^{PhPh}DippBeH]$_2$, 7.

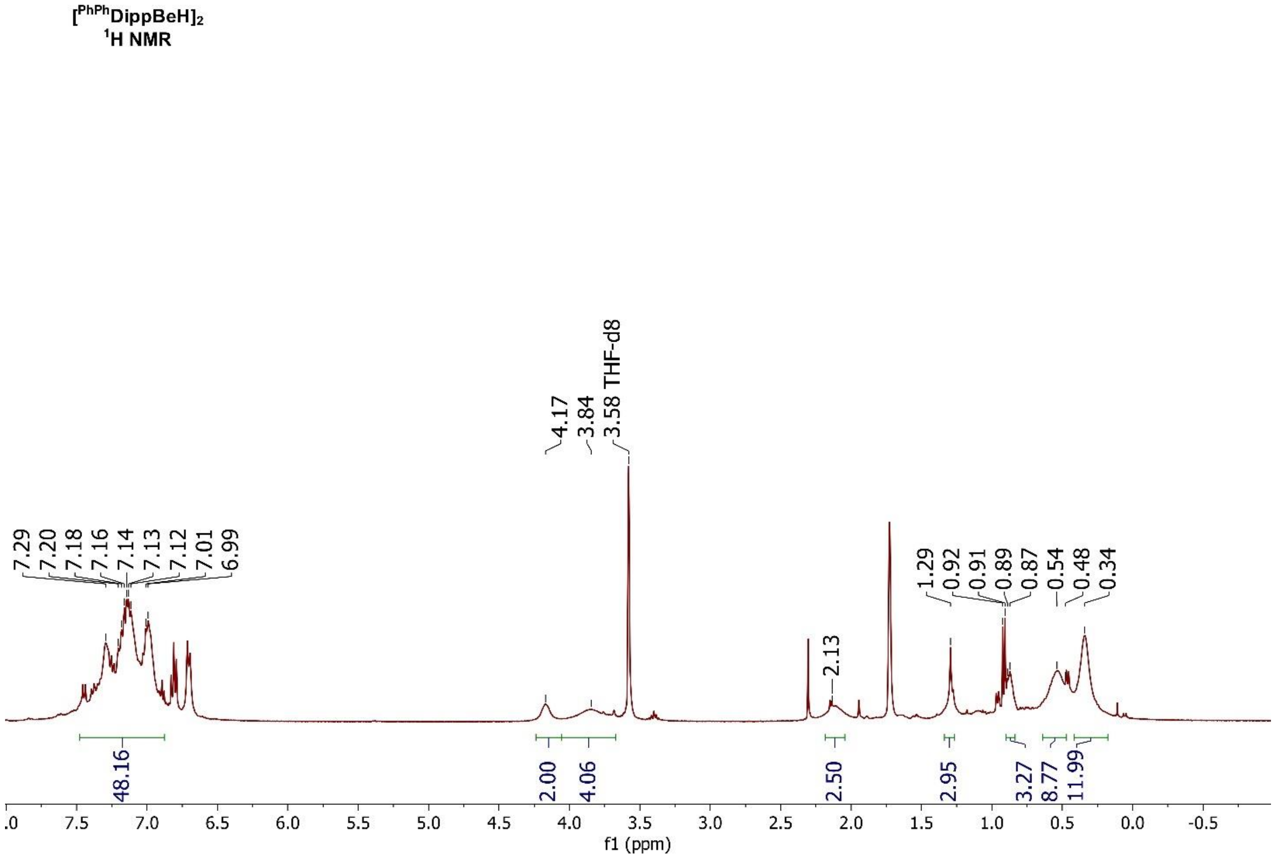

Supplementary Figure 24. ^{1}H NMR spectrum of [^{PhPh}DippBe-μ$_2$-H]$_2$ in D$_8$-THF at 298 K.

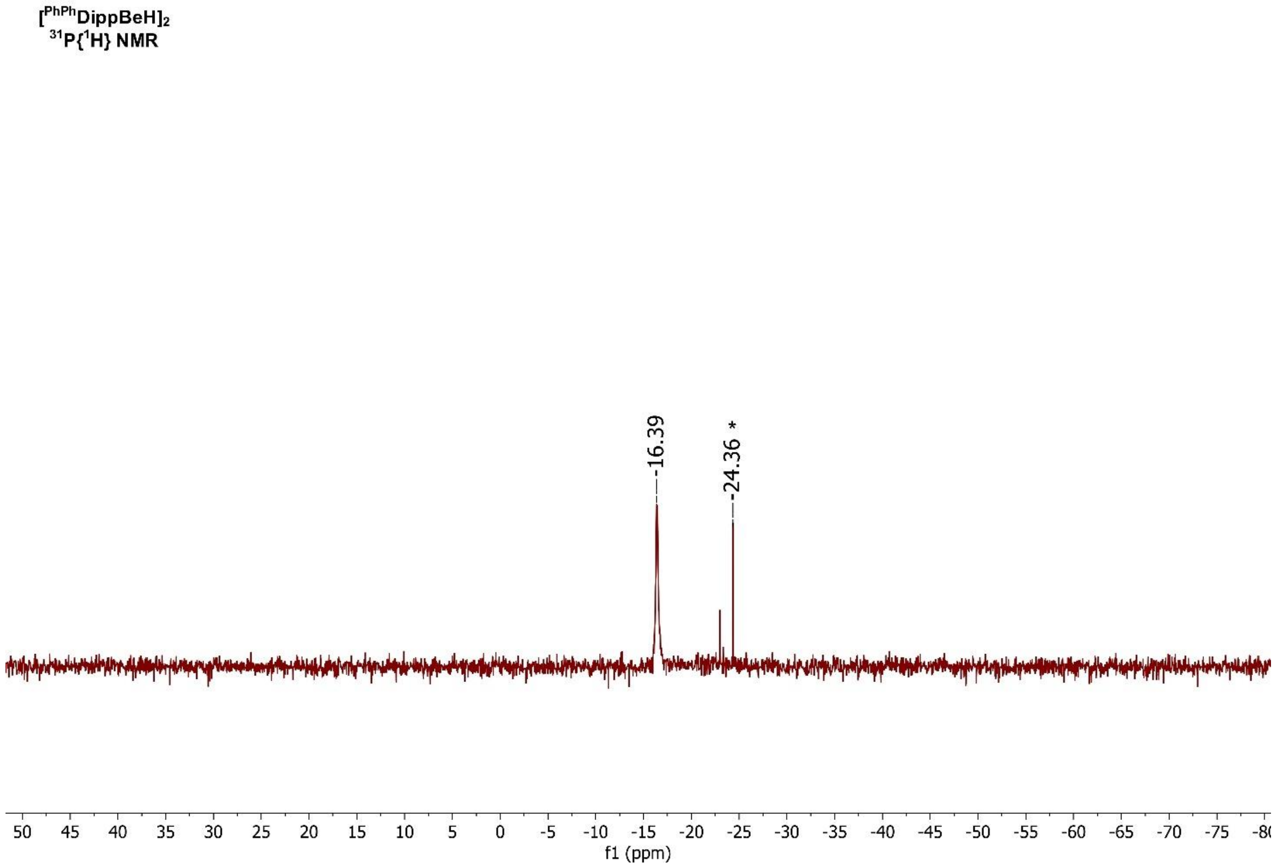

Supplementary Figure 25. $^{31}P\{^{1}H\}$ NMR spectrum of [^{PhPh}DippBe-μ_2-H]$_2$ in D_8-THF at 298 K. * *Denotes a small amount of protonated ligand, ^{PhPh}DippNH, formed when heating the sample in order to dissolve it.*

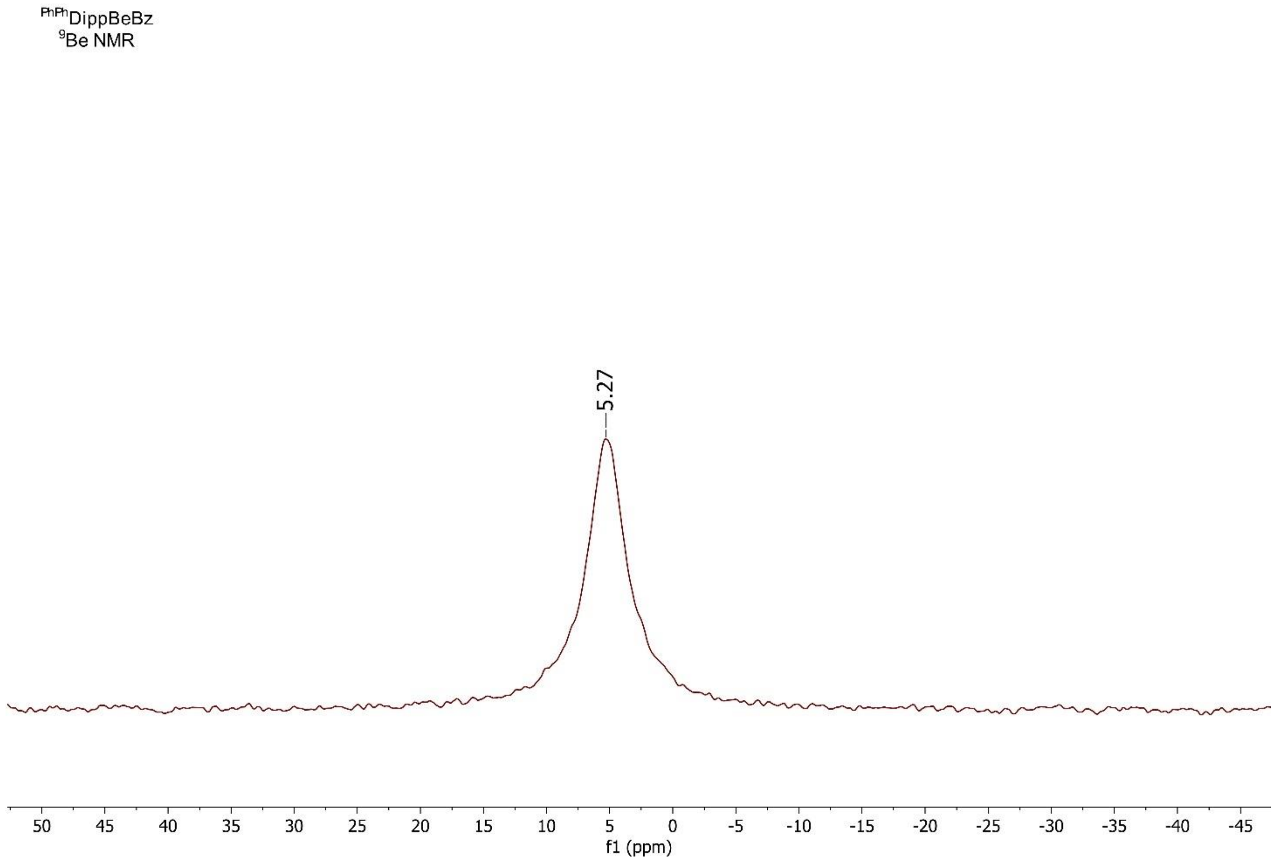

Supplementary Figure 26. ^{9}Be NMR spectrum of [^{PhPh}DippBe-μ_2-H]$_2$ in D_8-THF at 298 K.

[^{iPPh}DippBeH]$_2$, 8.

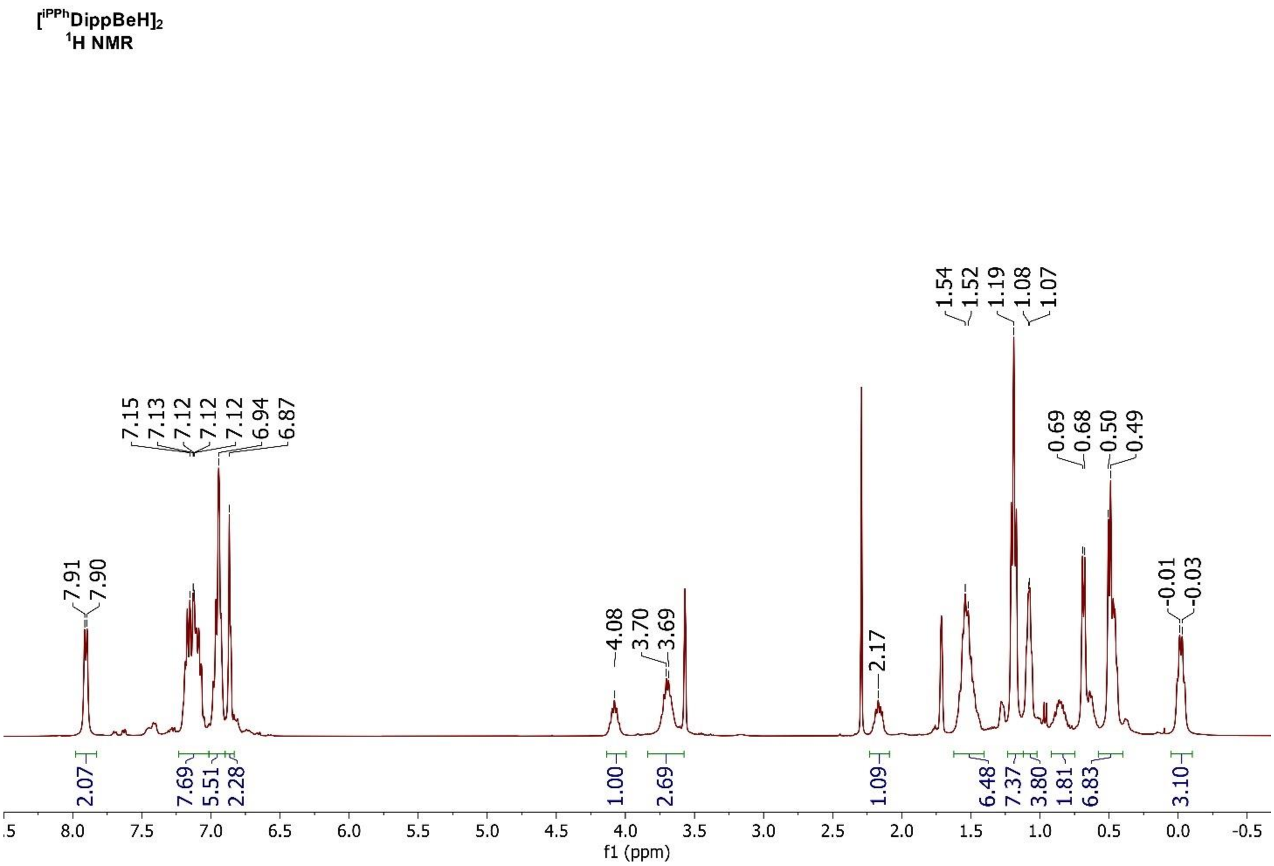

Supplementary Figure 27. ^{1}H NMR spectrum of [^{iPPh}DippBe-μ$_2$-H]$_2$ in D$_8$-THF at 338 K.

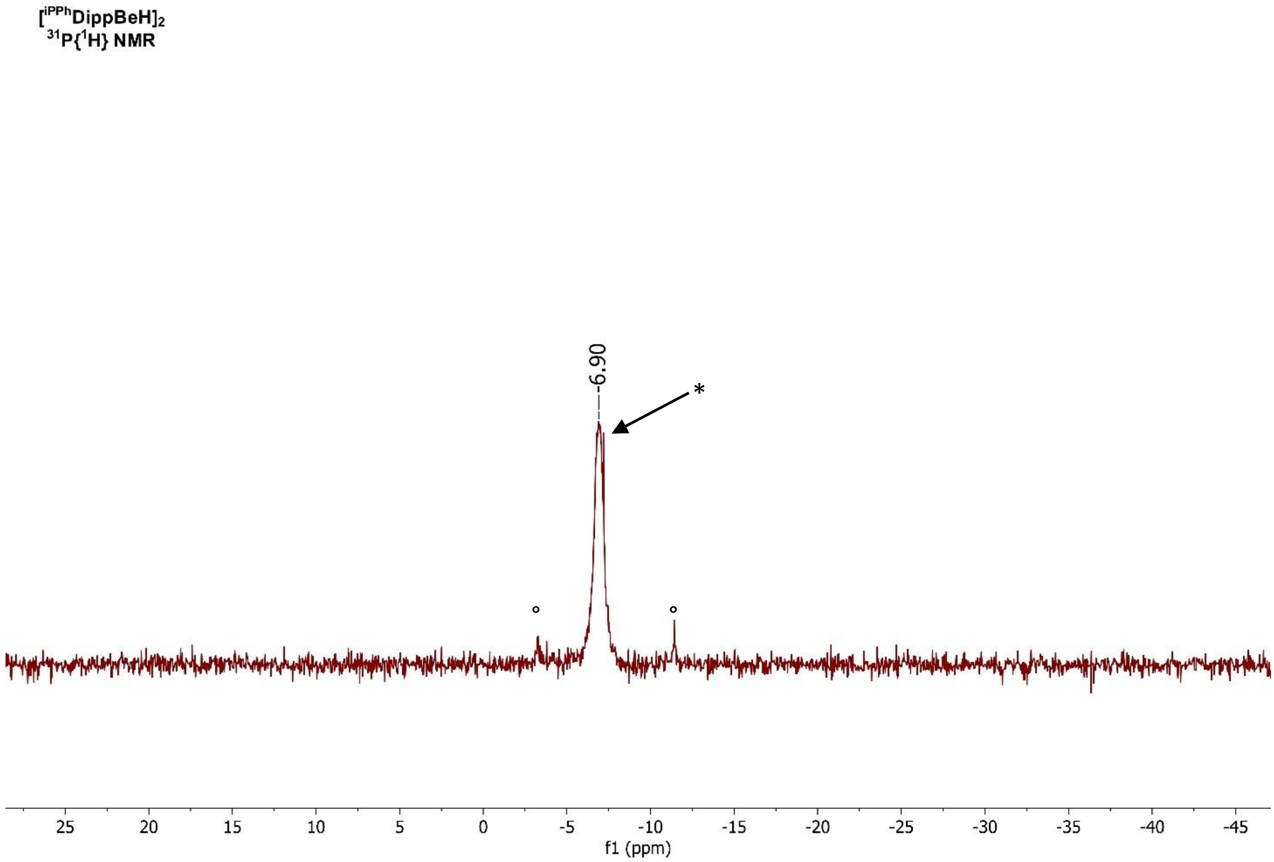

Supplementary Figure 28. ^{31}P{^{1}H} NMR spectrum of [^{iPPh}DippBe-μ$_2$-H]$_2$ in D$_8$-THF at 338 K. * *Denotes a small amount of protonated ligand, ^{iPrPh}DippNH; ° Denotes unidentified impurities.*

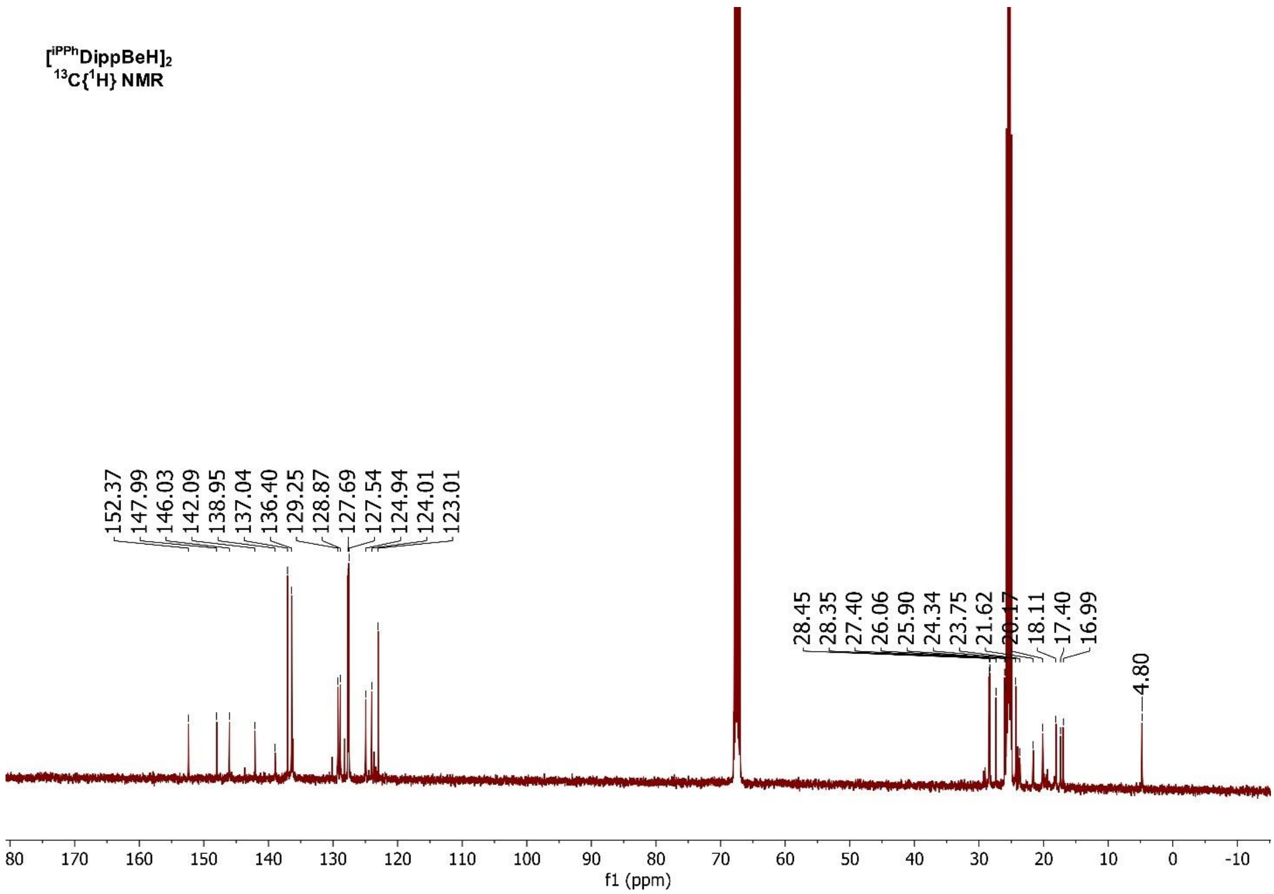

Supplementary Figure 29. $^{13}C\{^{1}H\}$ NMR spectrum of [^{iPPh}DippBe-μ$_2$-H]$_2$ in D$_8$-THF at 298 K.

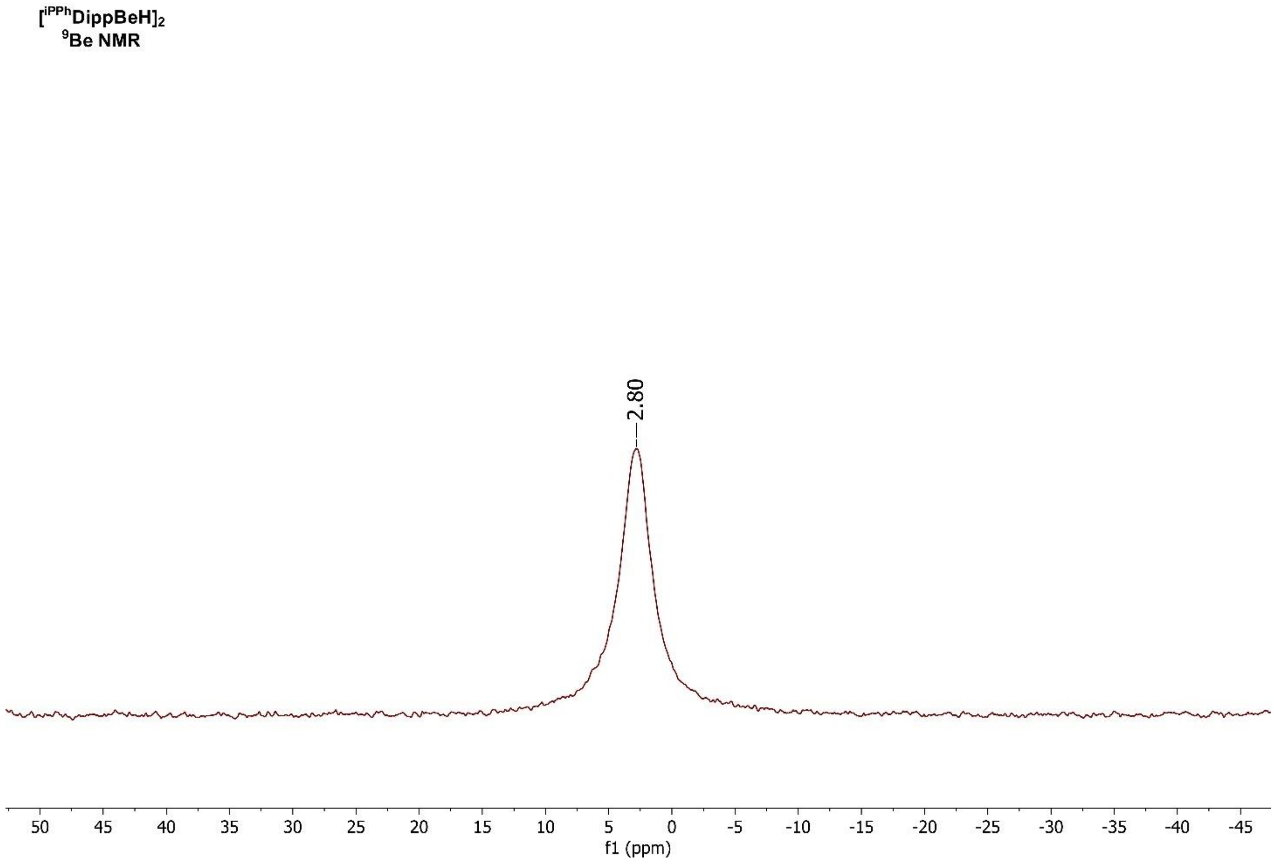

Supplementary Figure 30. ^{9}Be NMR spectrum of [^{iPPh}DippBe-μ$_2$-H]$_2$ in D$_8$-THF at 338 K.

^{PhiP}DippBeH·NHC, 9.

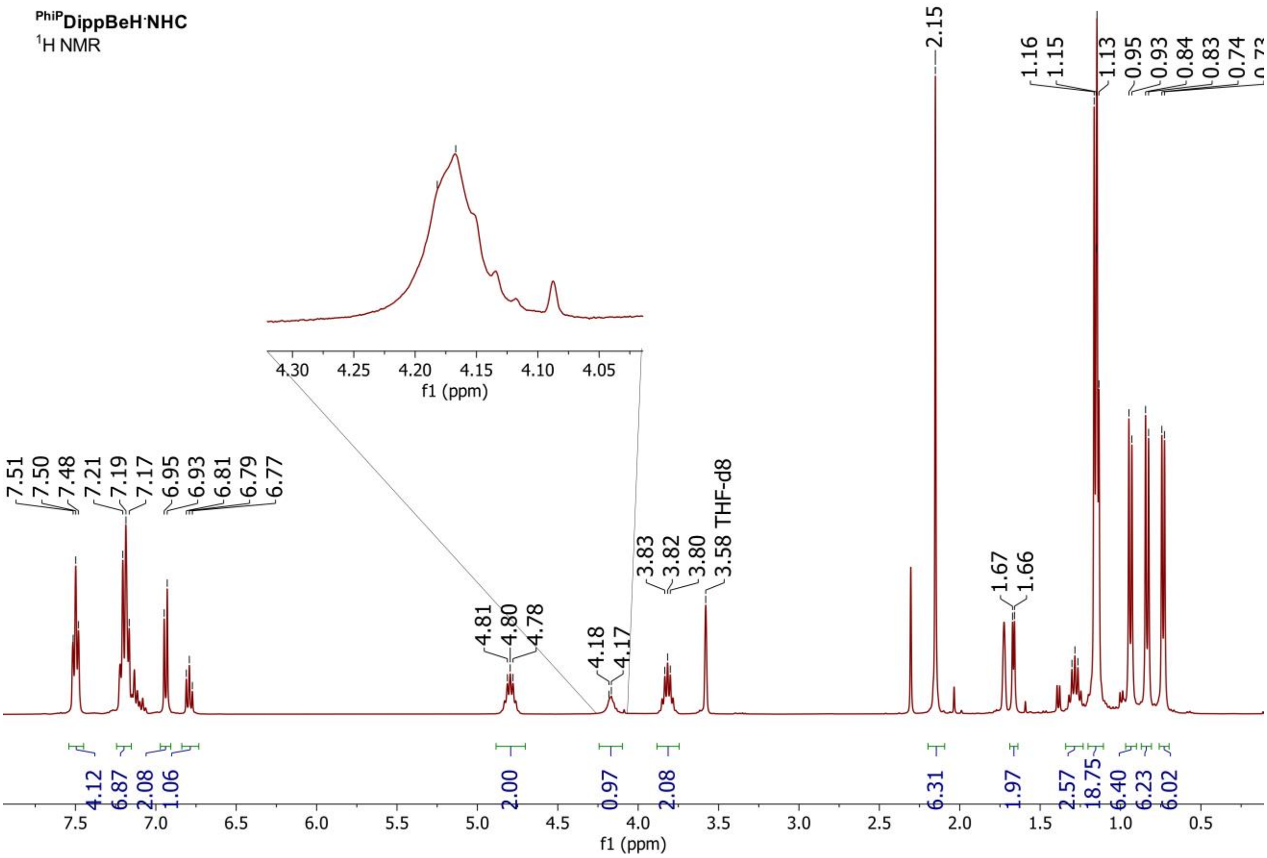

Supplementary Figure 31. ^{1}H NMR spectrum of ^{PhiP}DippBeH·NHC in C_6D_6 at ambient temperature.

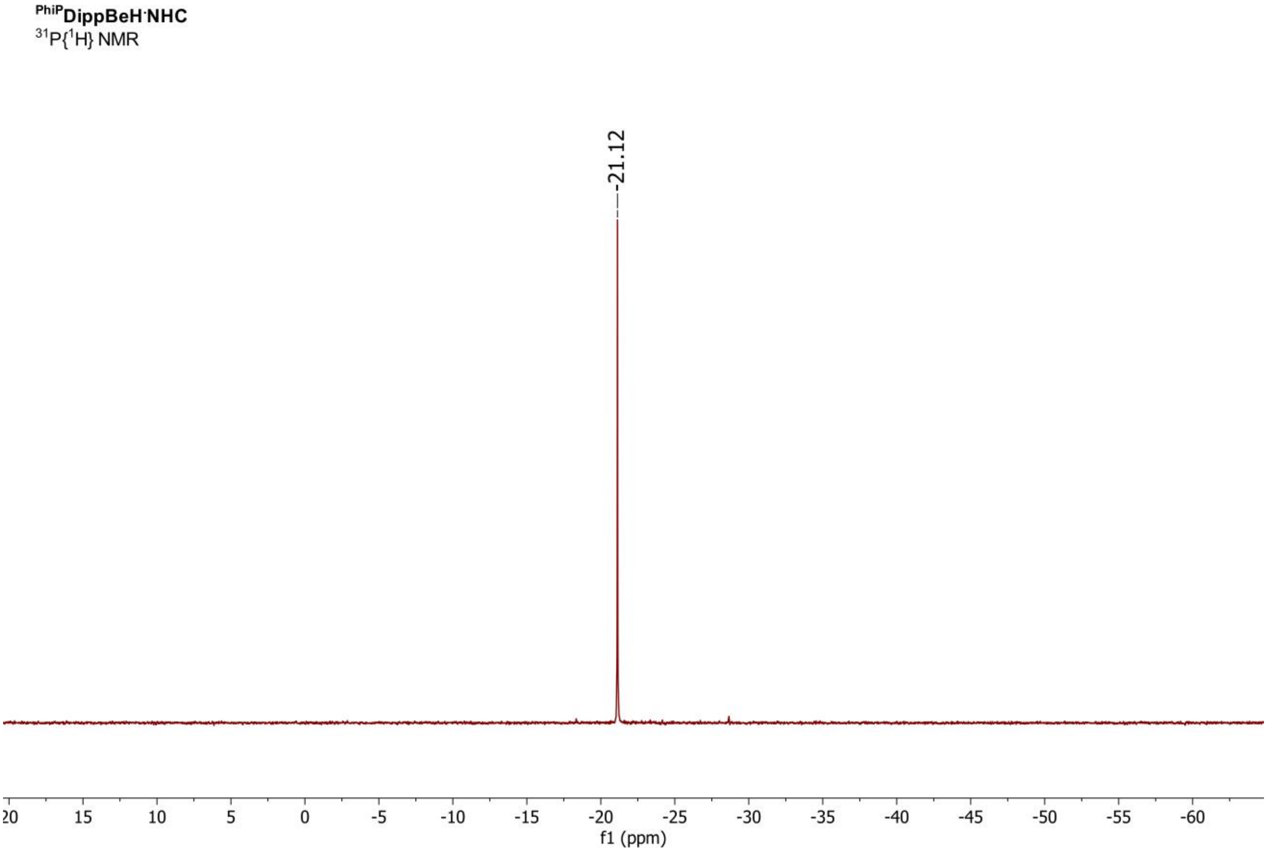

Supplementary Figure 32. ^{31}P{^{1}H} NMR spectrum of ^{PhiP}DippBeH·NHC in C_6D_6 at ambient temperature.

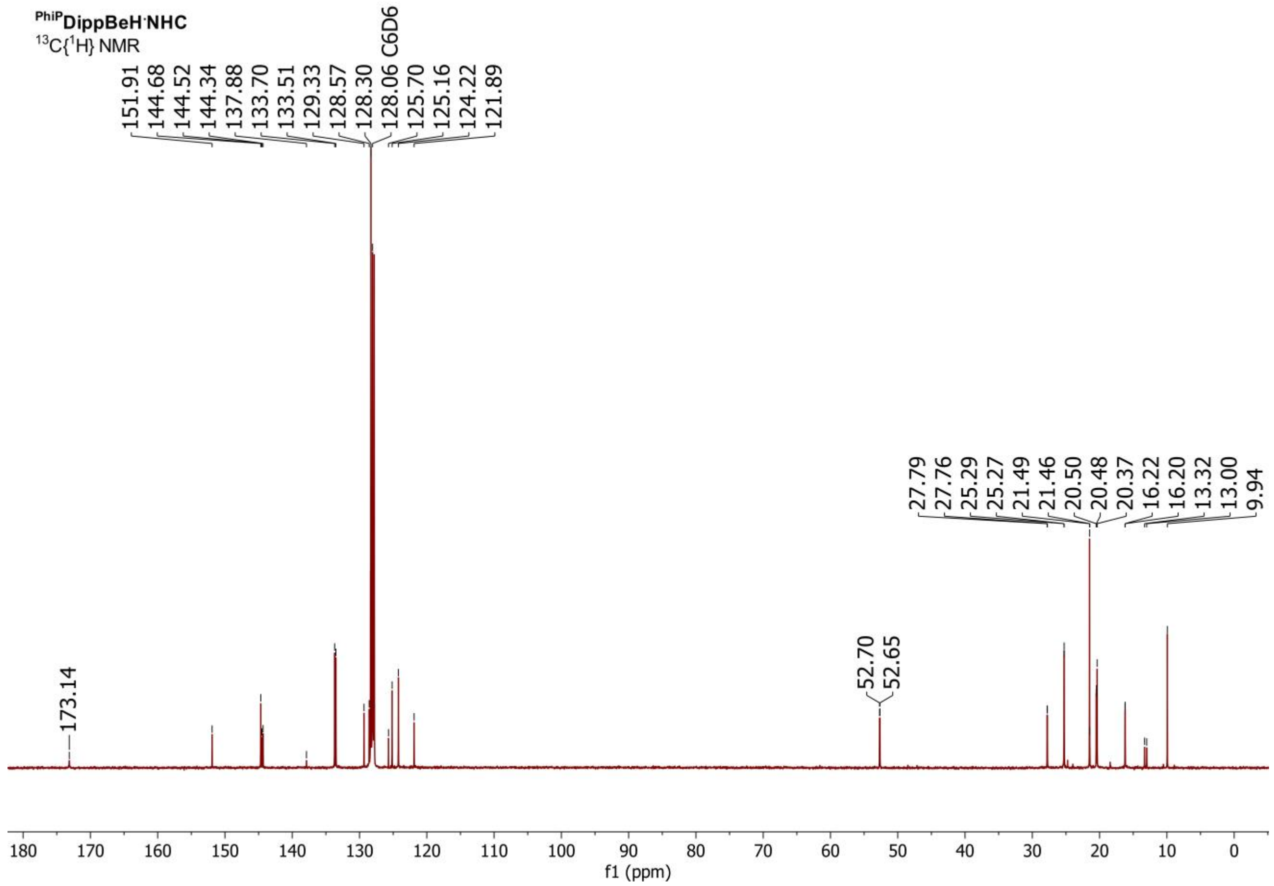

Supplementary Figure 33. $^{13}C\{^1H\}$ NMR spectrum of ^{PhiP}DippBeH·NHC in C_6D_6 at ambient temperature.

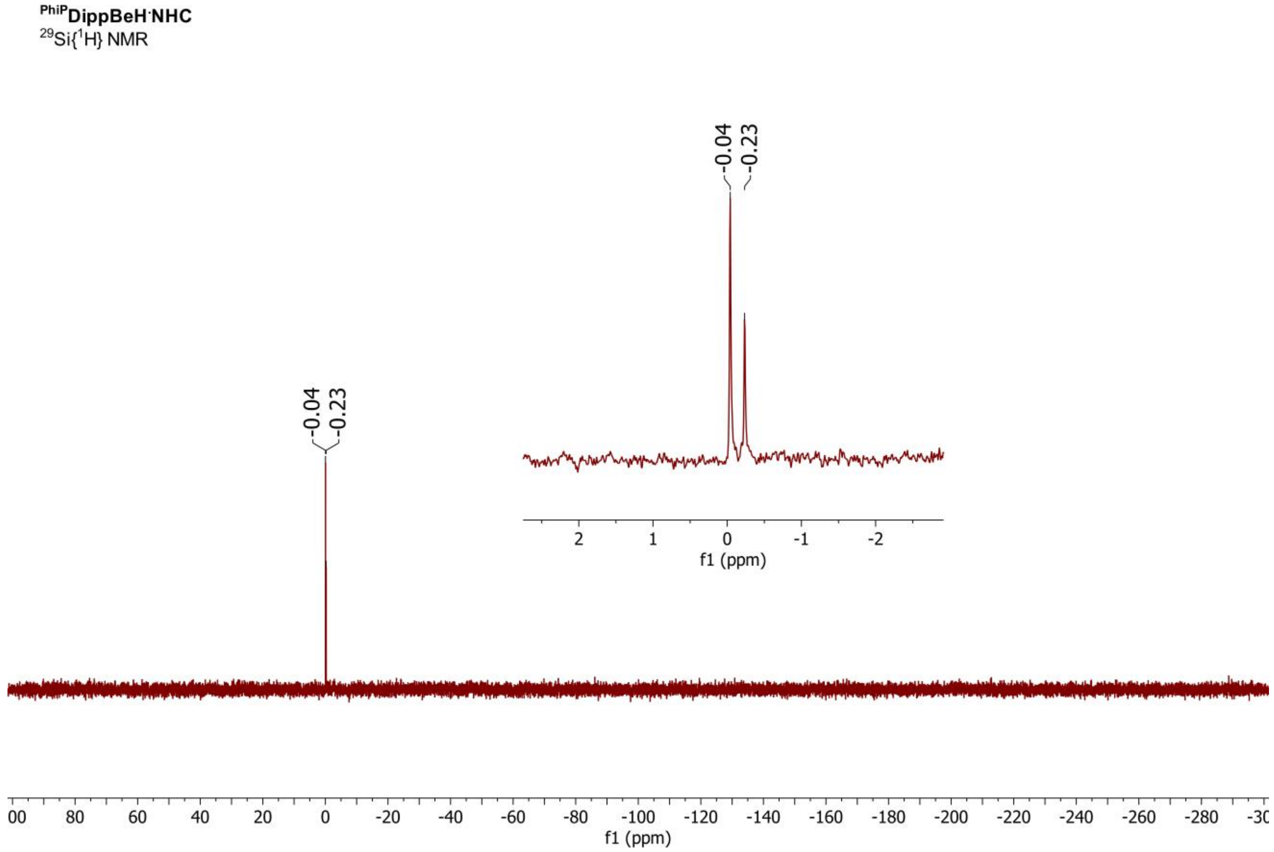

Supplementary Figure 34. $^{29}Si\{^1H\}$ NMR spectrum of ^{PhiP}DippBeH·NHC in C_6D_6 at ambient temperature.

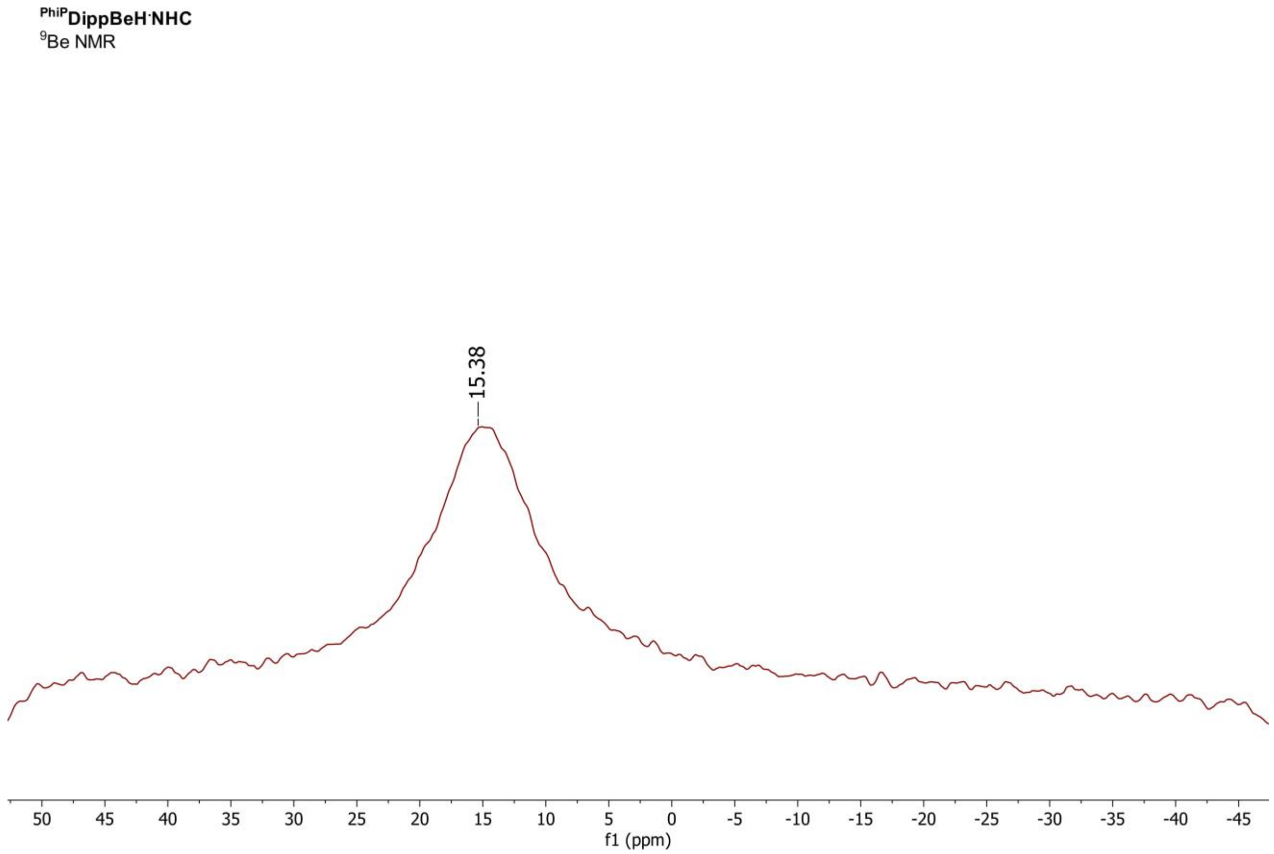

Supplementary Figure 35. ^{9}Be NMR spectrum of ^{PhiP}DippBeH·NHC in C_6D_6 at ambient temperature.

[^{PhiP}DippBeOC(H)O]$_2$, 10.

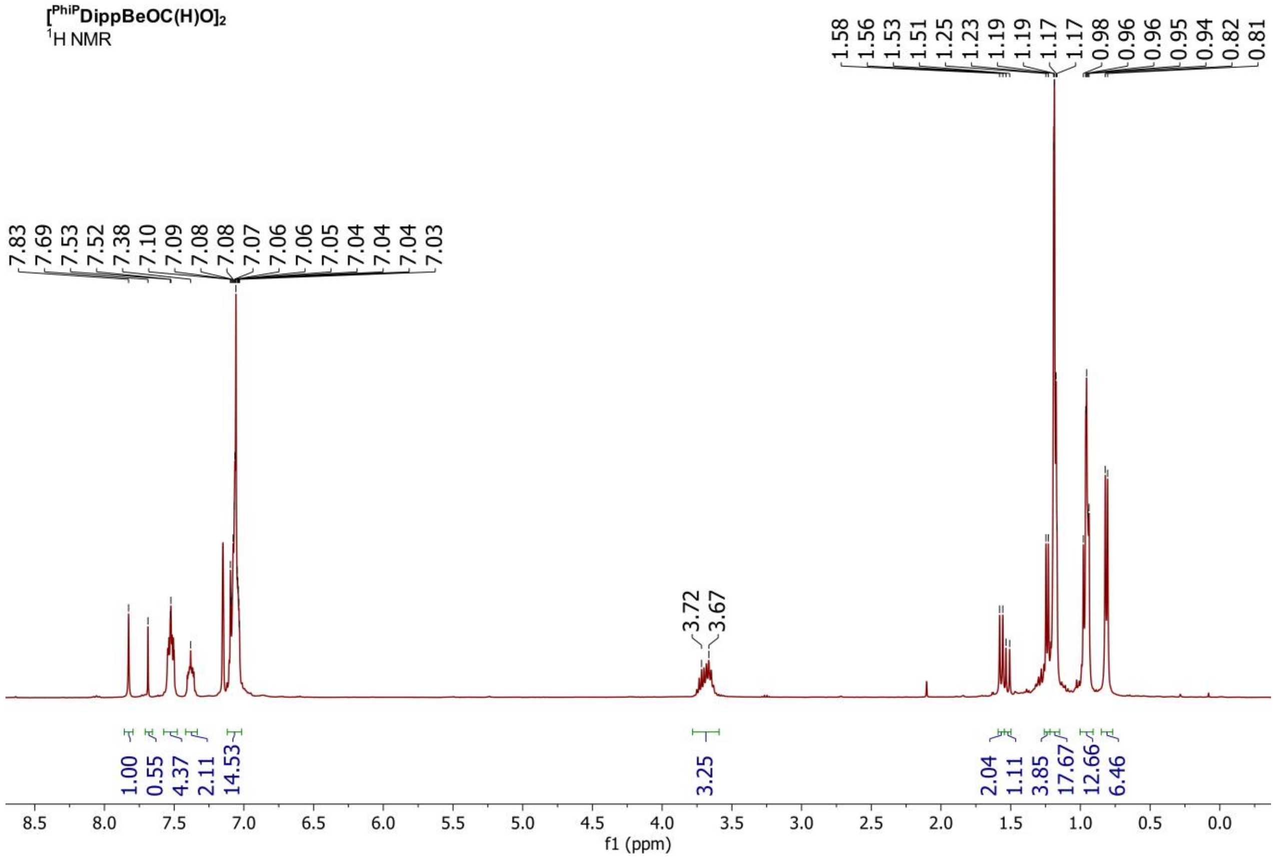

Supplementary Figure 36. ^{1}H NMR spectrum of [^{PhiP}DippBe-μ_2-{OC(H)O}]$_2$ in C_6D_6 at ambient temperature.

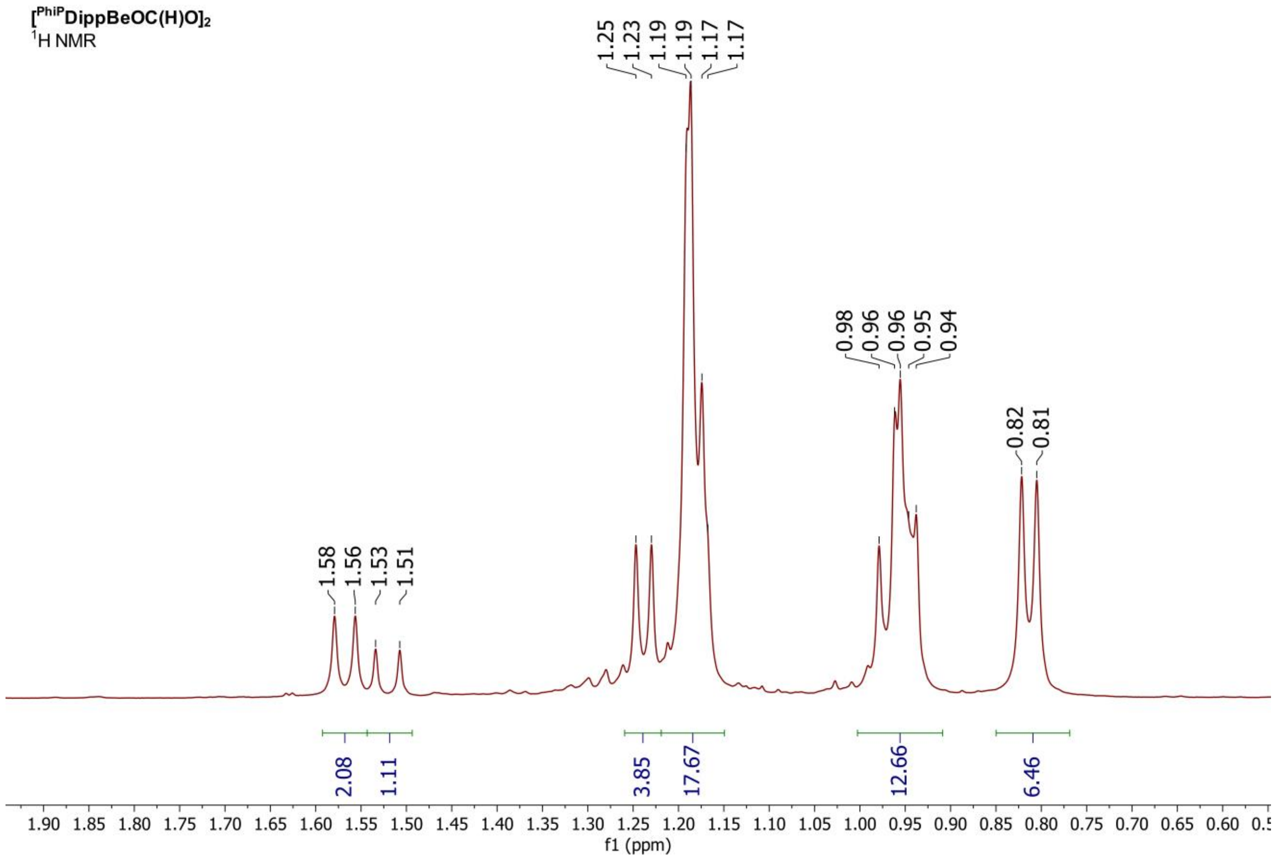

Supplementary Figure 37. ^{1}H NMR spectrum of [^{PhiP}DippBe-μ$_2$-{OC(H)O}]$_2$ (0.55-1.95 ppm) in C_6D_6 at ambient temperature.

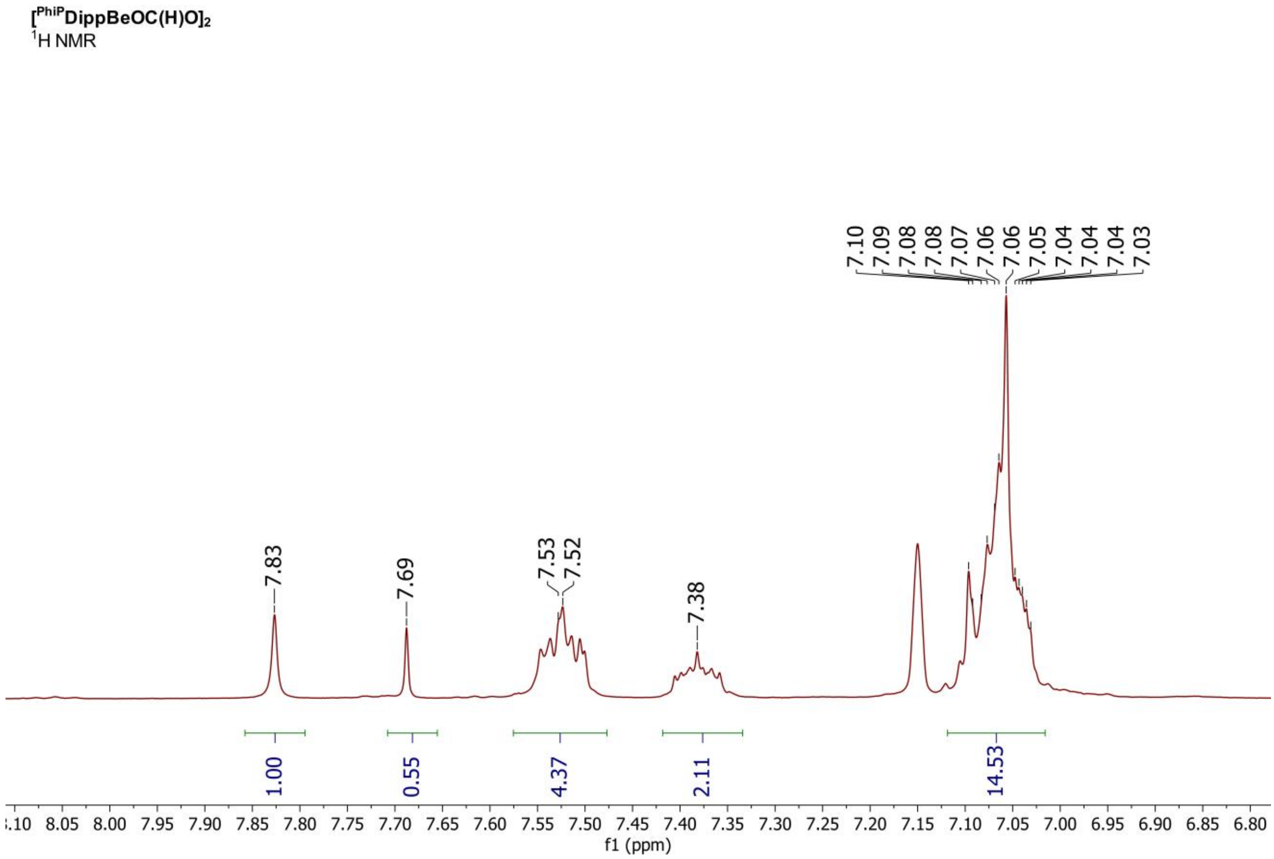

Supplementary Figure 38. ^{1}H NMR spectrum of [^{PhiP}DippBe-μ$_2$-{OC(H)O}]$_2$ (6.80-8.10 ppm) in C_6D_6 at ambient temperature.

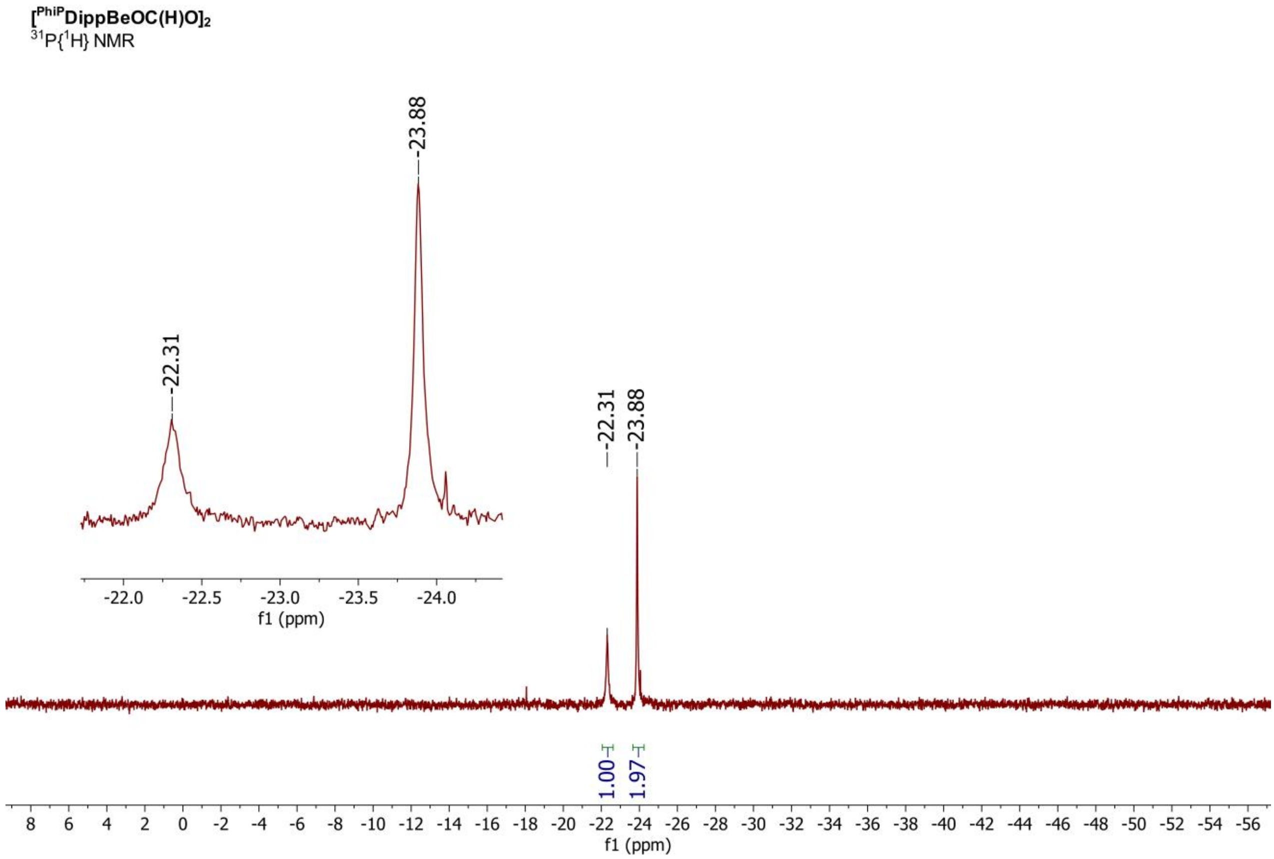

Supplementary Figure 39. $^{31}P\{^{1}H\}$ NMR spectrum of $[^{PhiP}DippBe\text{-}\mu_2\text{-}\{OC(H)O\}]_2$ in C_6D_6 at ambient temperature.

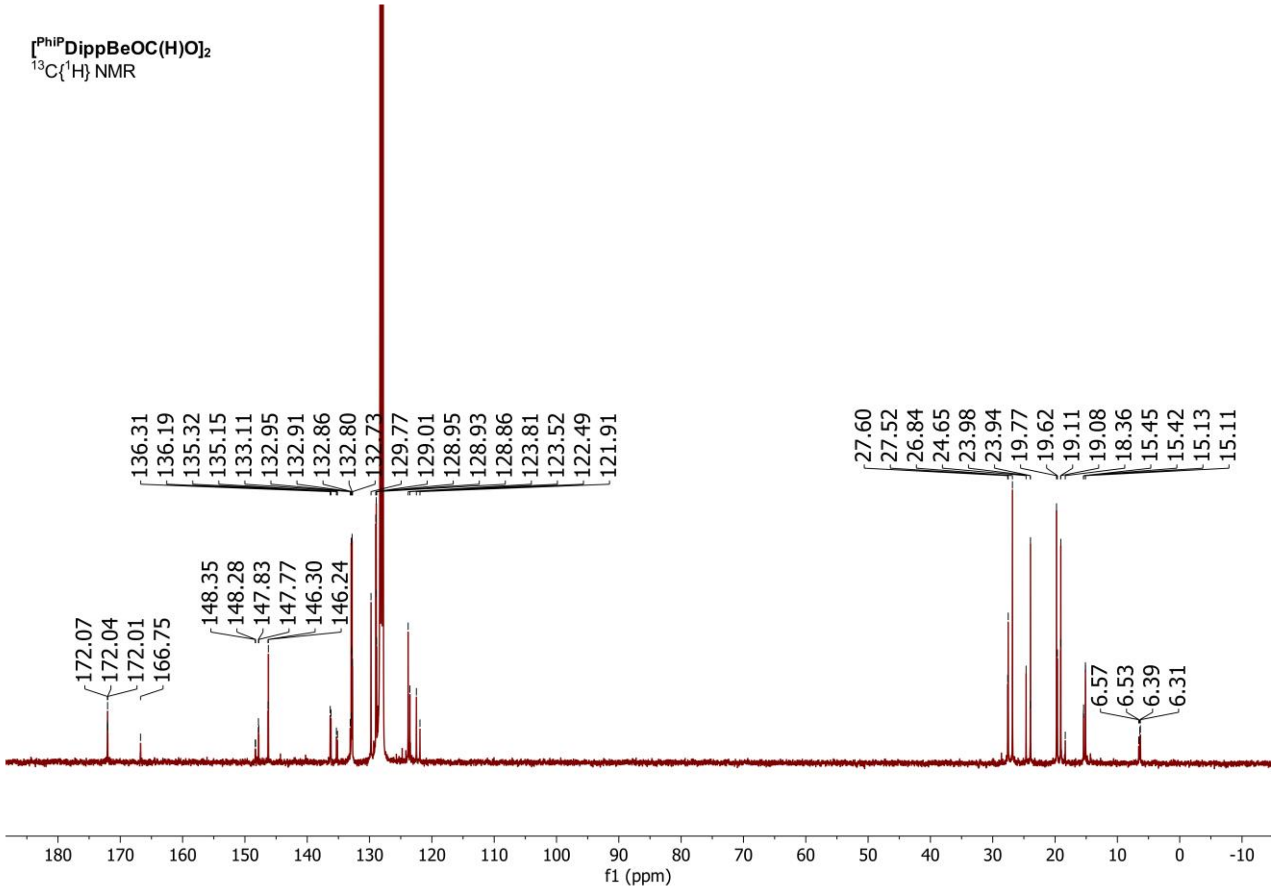

Supplementary Figure 40. $^{13}C\{^{1}H\}$ NMR spectrum of $[^{PhiP}DippBe\text{-}\mu_2\text{-}\{OC(H)O\}]_2$ in C_6D_6 at ambient temperature.

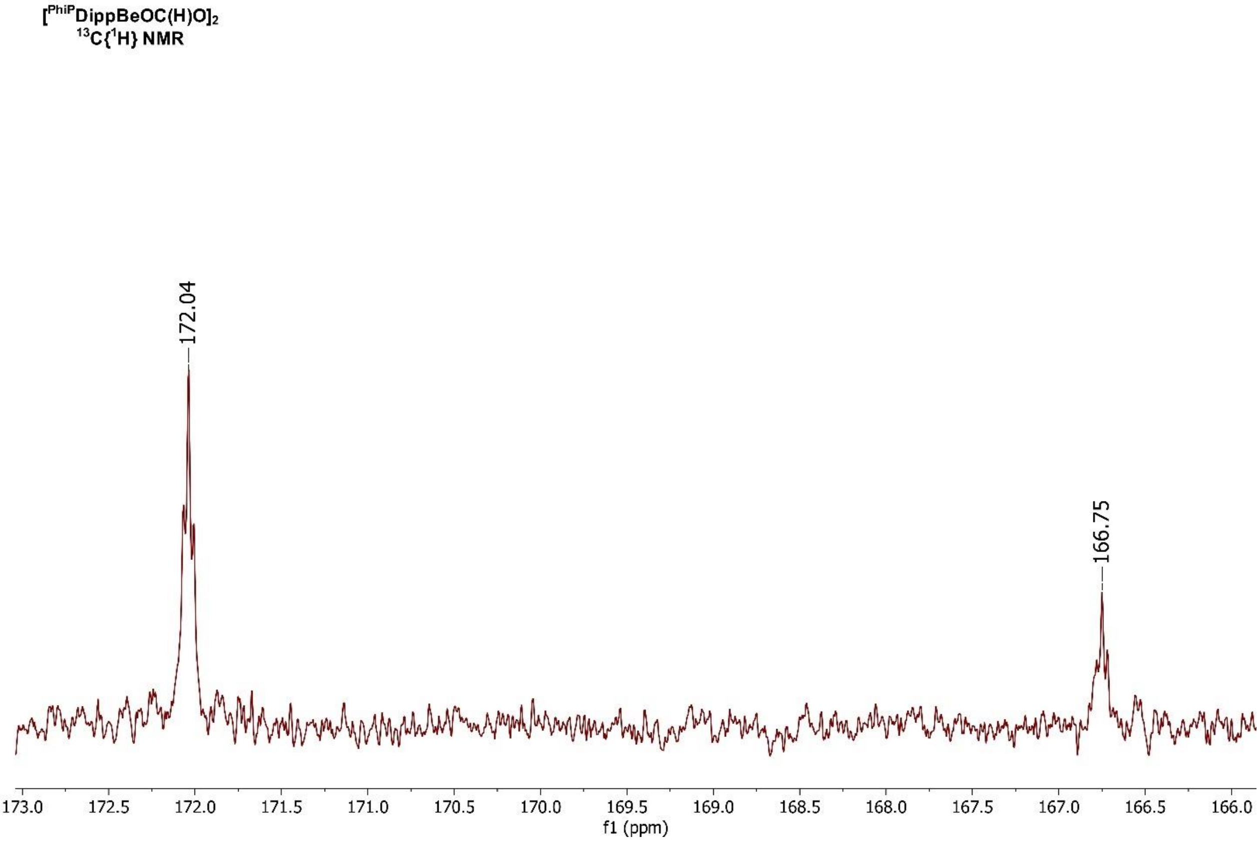

Supplementary Figure 41. $^{13}C\{^1H\}$ NMR spectrum of [^{PhiP}DippBe-μ_2-{OC(H)O}]$_2$ (166-173 ppm) in C_6D_6 at ambient temperature.

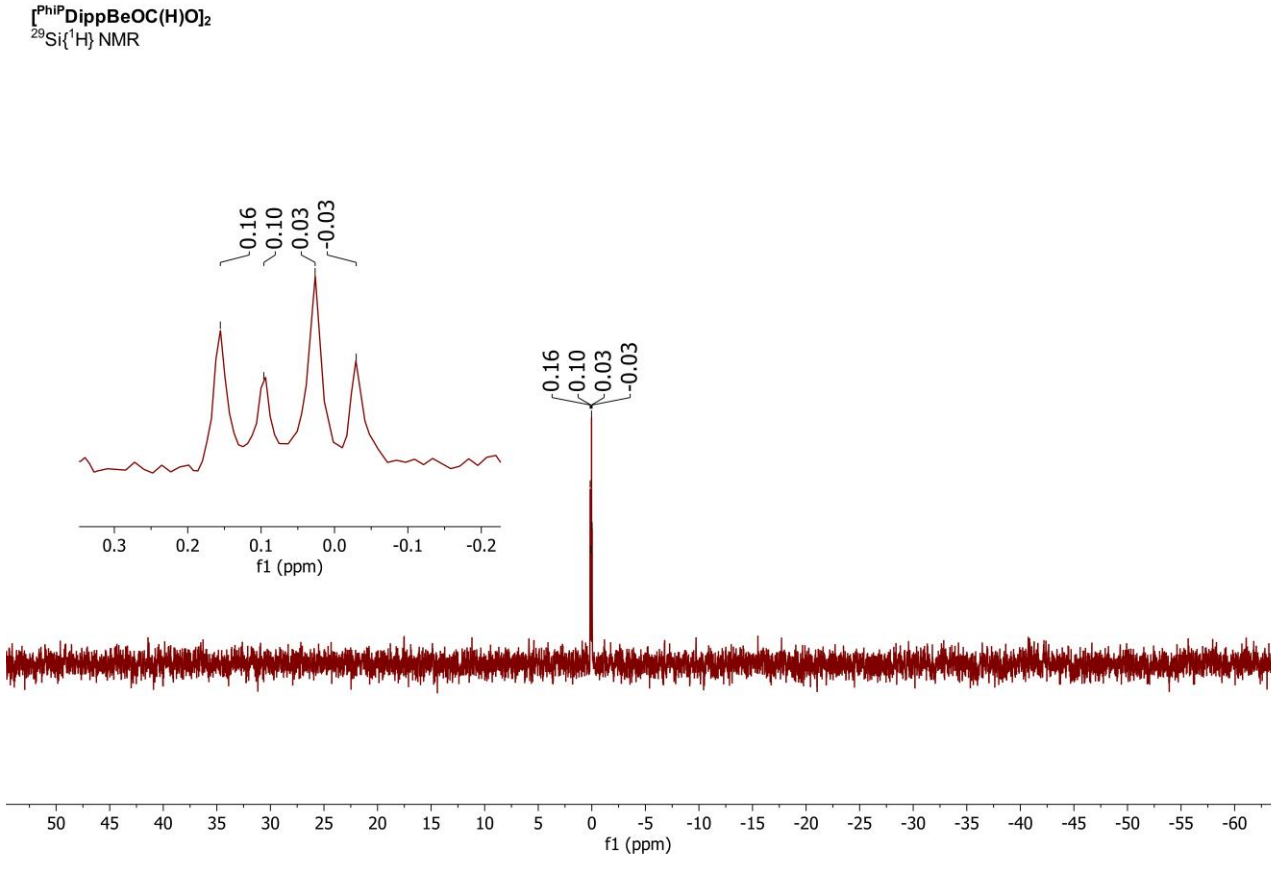

Supplementary Figure 42. $^{29}Si\{^1H\}$ IG NMR spectrum of [^{PhiP}DippBe-μ_2-{OC(H)O}]$_2$ in C_6D_6 at ambient temperature.

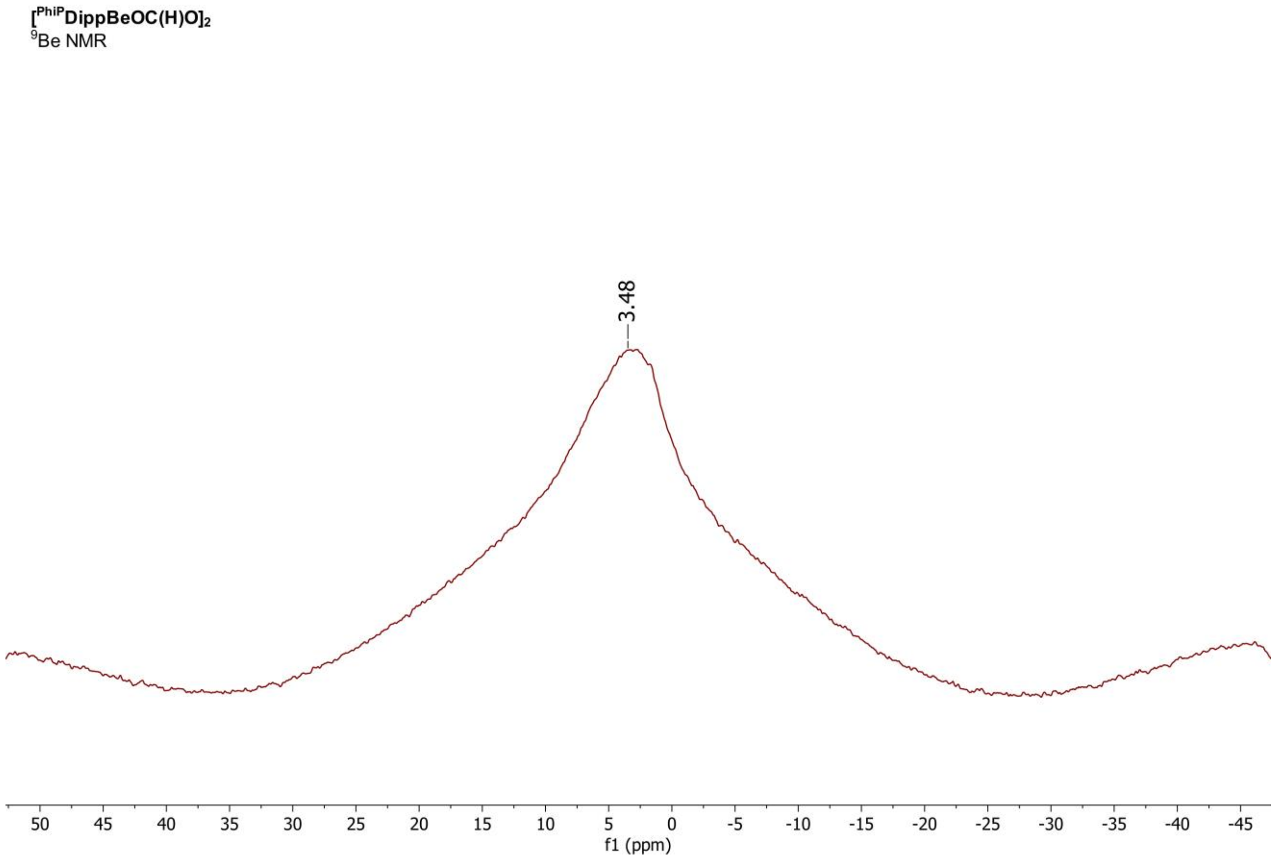

Supplementary Figure 43. ^{9}Be NMR spectrum of [^{PhiP}DippBe-μ$_2$-{OC(H)O}]$_2$ in C_6D_6 at ambient temperature.

[^{PhiP}DippBeC(H)O]$_2$, 11.

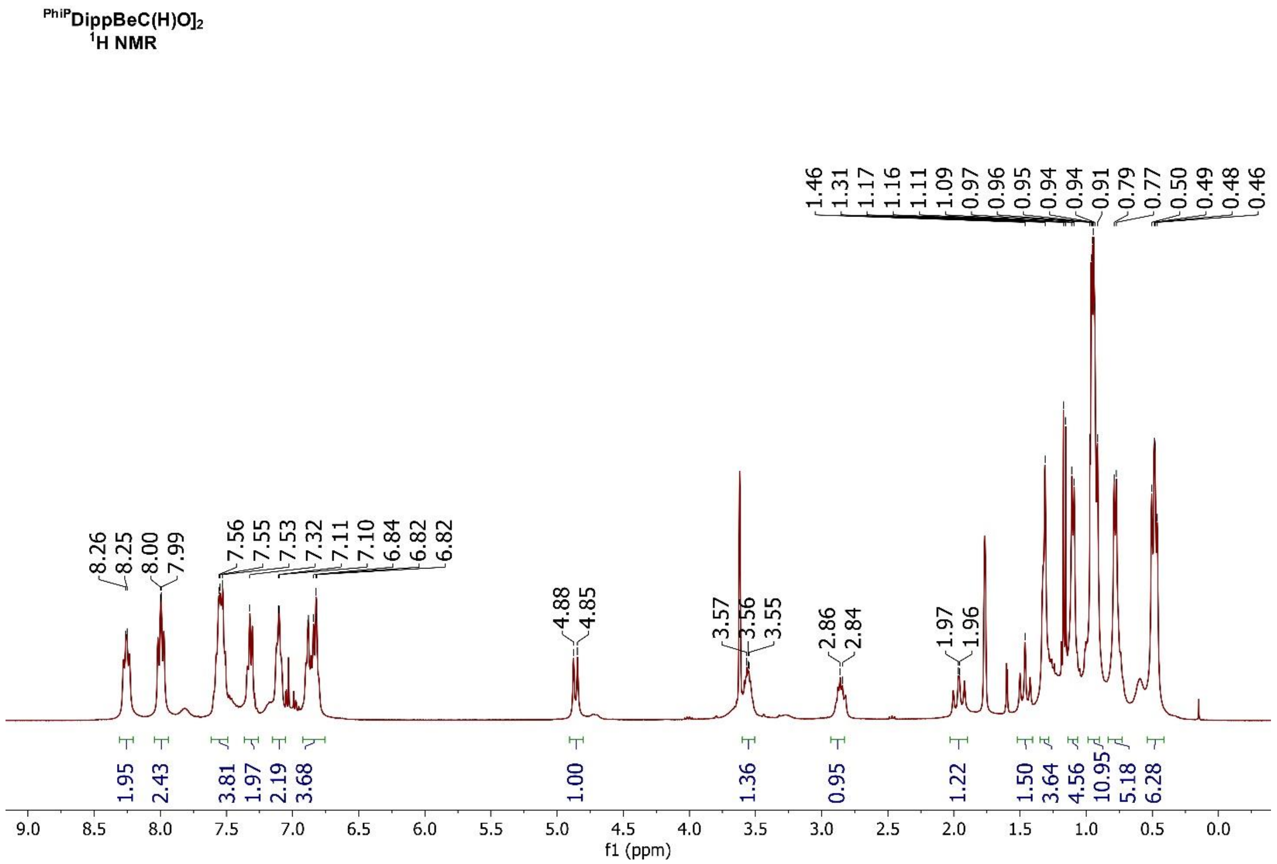

Supplementary Figure 44. ^{1}H NMR spectrum of [^{PhiP}DippBeC(H)O]$_2$ in D_8-THF at ambient temperature.

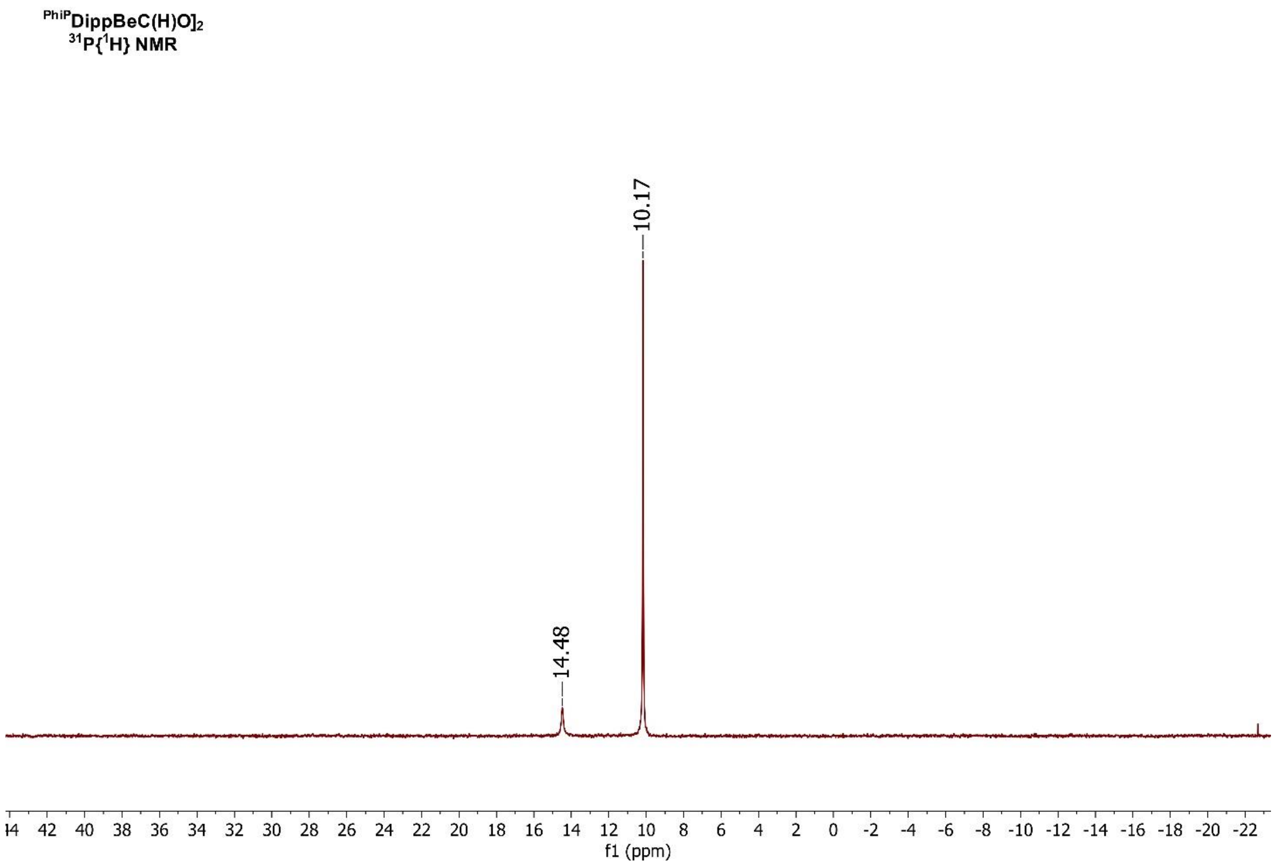

Supplementary Figure 45. $^{31}P\{^1H\}$ NMR spectrum of [^{PhiP}DippBeC(H)O]$_2$ in D_8-THF at ambient temperature.

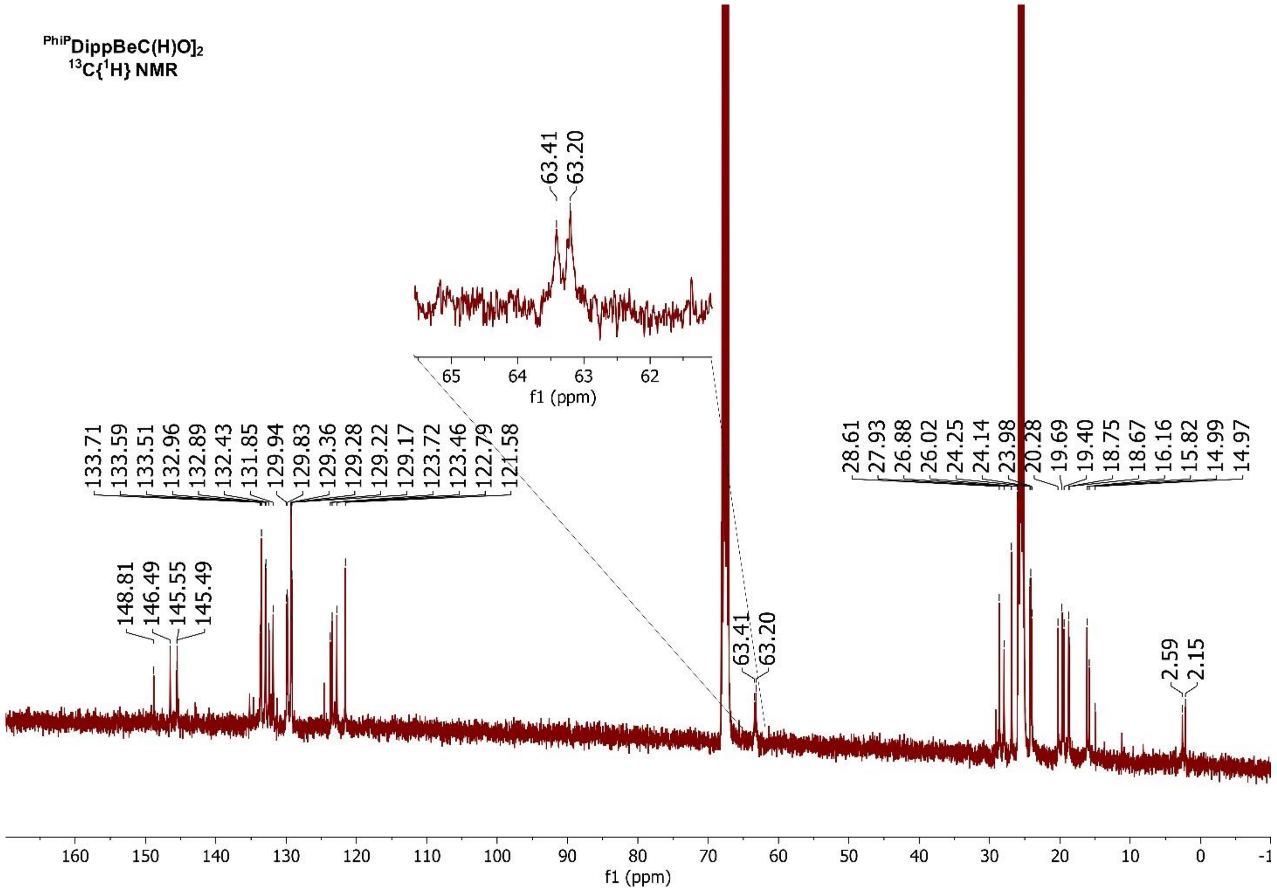

Supplementary Figure 46. $^{13}C\{^1H\}$ NMR spectrum of [^{PhiP}DippBeC(H)O]$_2$ in D_8-THF at ambient temperature.

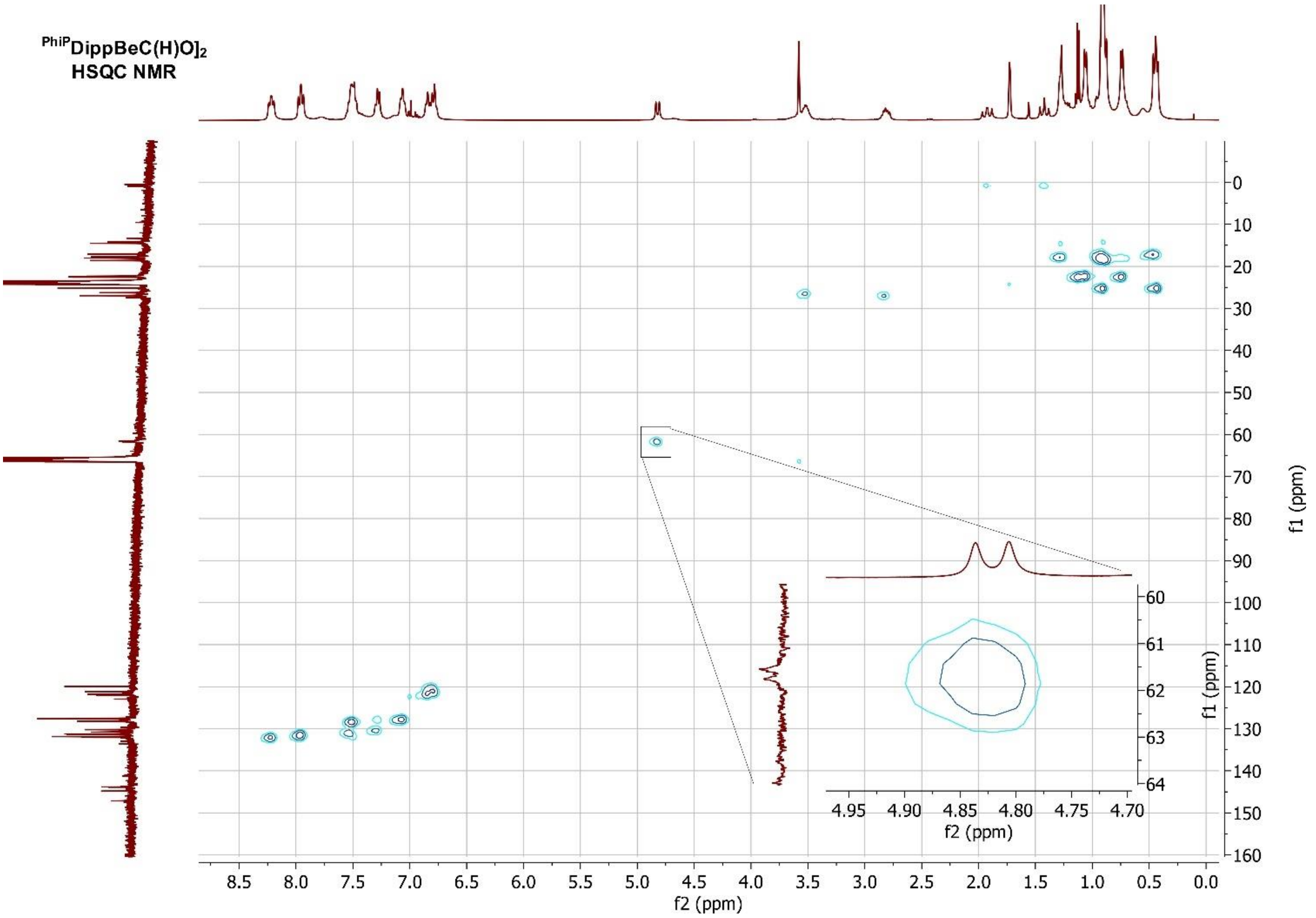

Supplementary Figure 47. HSQC NMR spectrum of [^{PhiP}DippBeC(H)O]$_2$ in D_8-THF at ambient temperature.

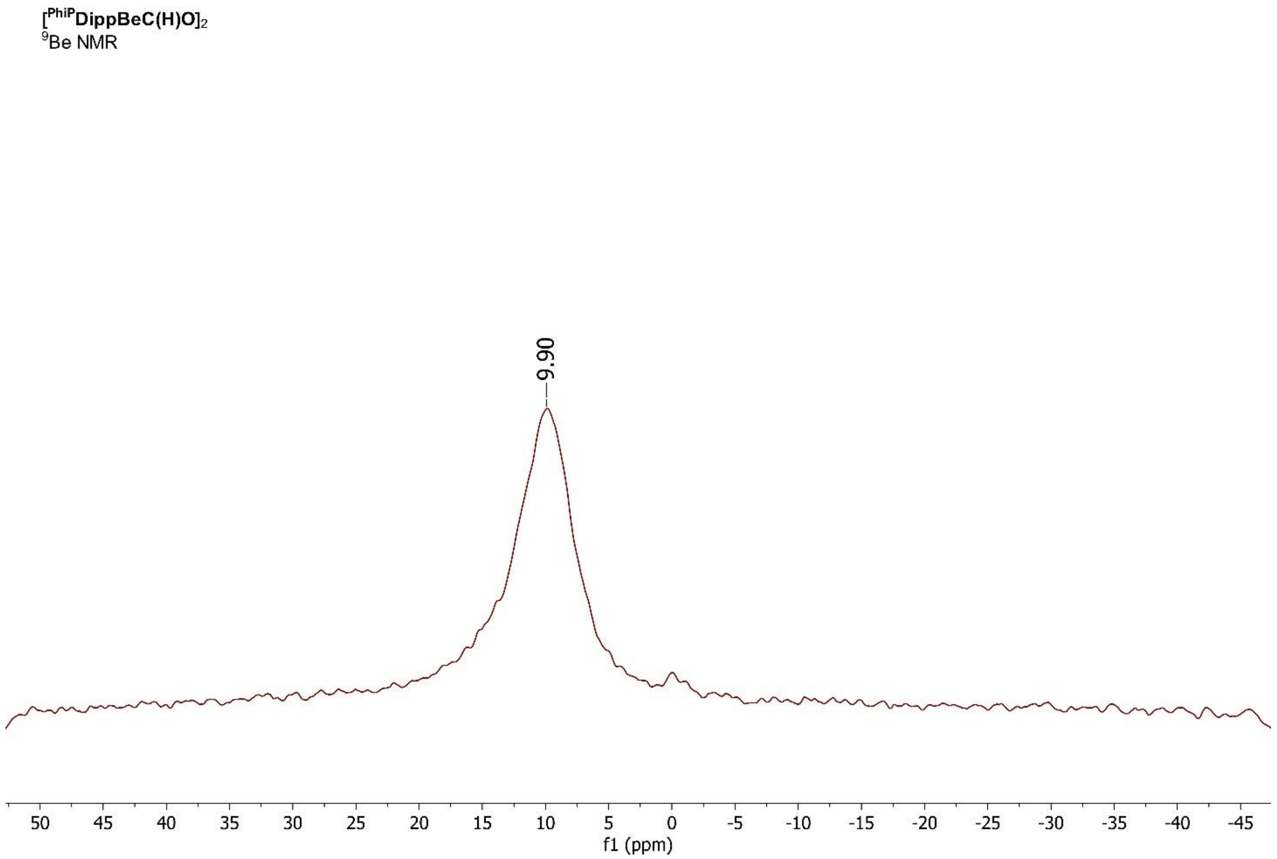

Supplementary Figure 48. ^{9}Be NMR spectrum of [^{PhiP}DippBeC(H)O]$_2$ in D_8-THF at ambient temperature.

[^{iPPh}DippBeC(H)O]$_2$, 12.

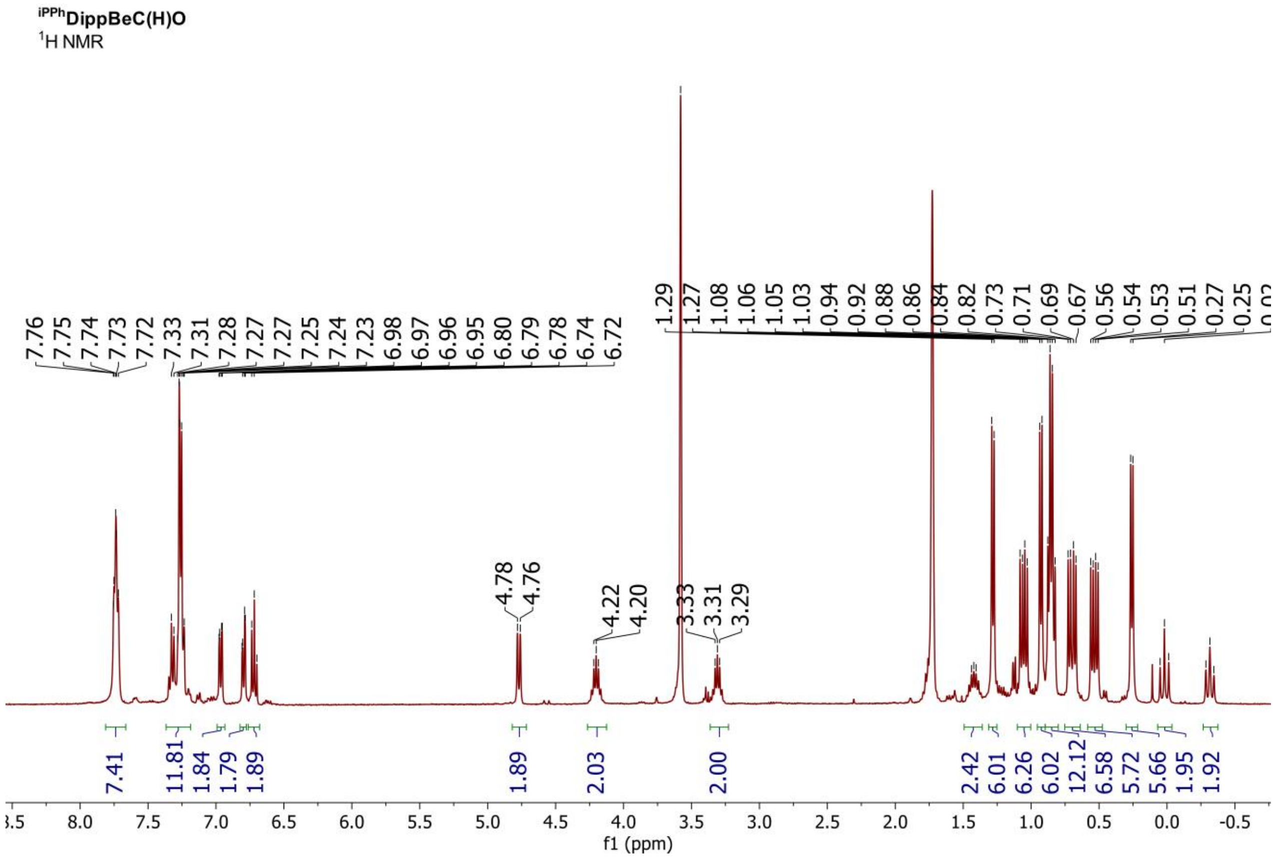

Supplementary Figure 49. ^{1}H NMR spectrum of [^{iPPh}DippBeC(H)O]$_2$ in D_8-THF at ambient temperature.

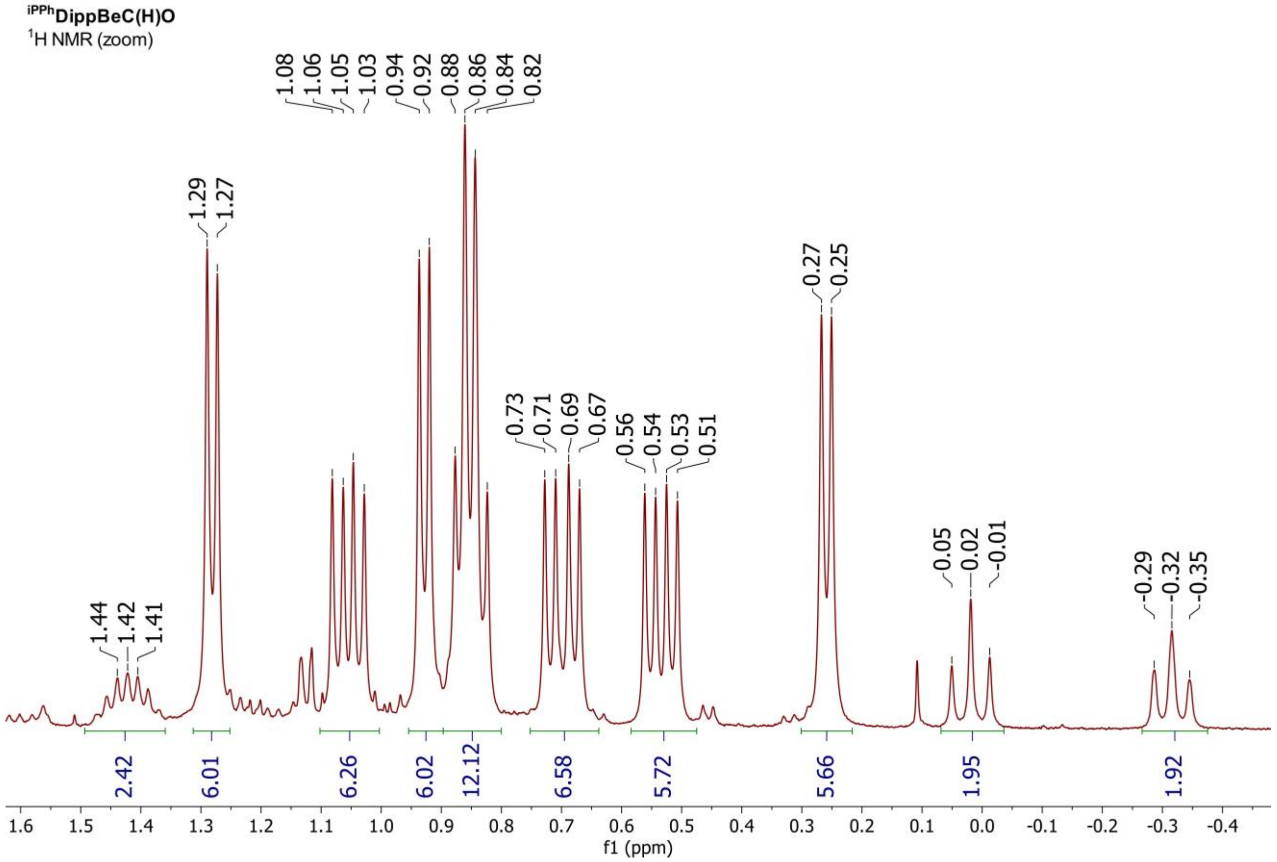

Supplementary Figure 50. ^{1}H NMR spectrum of [^{iPPh}DippBeC(H)O]$_2$ (-0.4-1.6 ppm) in D_8-THF at ambient temperature.

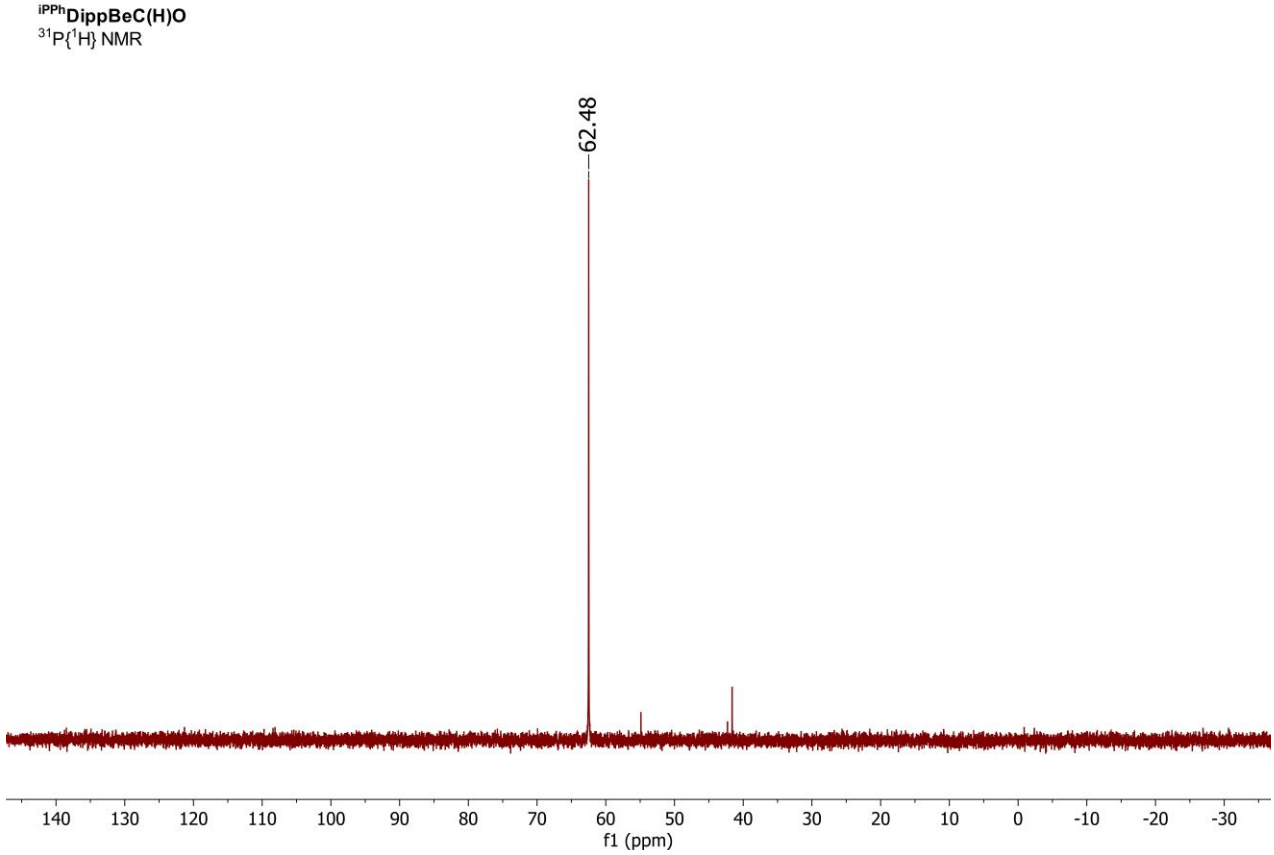

Supplementary Figure 51. $^{31}P\{^{1}H\}$ NMR spectrum of $[^{iPPh}DippBeC(H)O]_2$ in D_8-THF at ambient temperature.

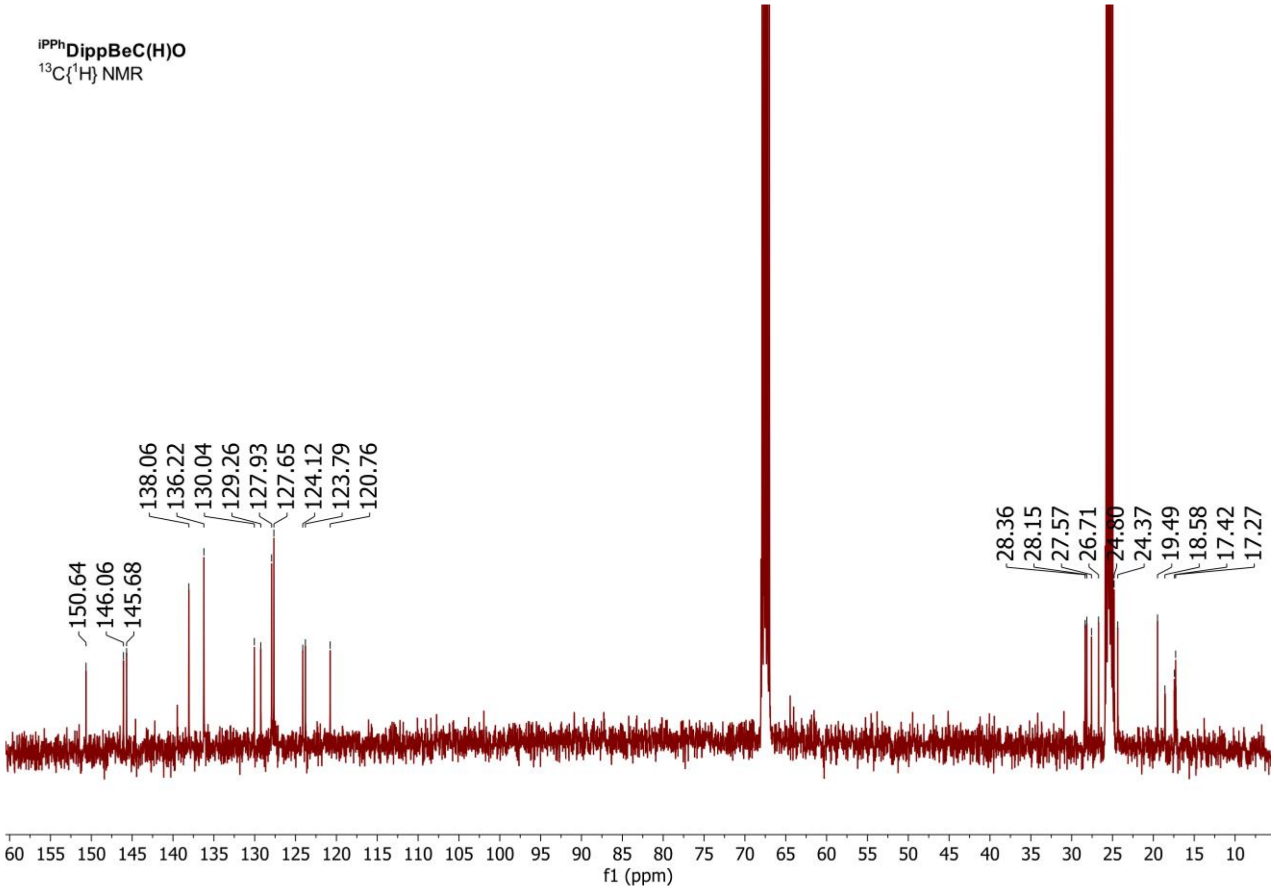

Supplementary Figure 52. $^{13}C\{^{1}H\}$ NMR spectrum of $[^{iPPh}DippBeC(H)O]_2$ in D_8-THF at ambient temperature.

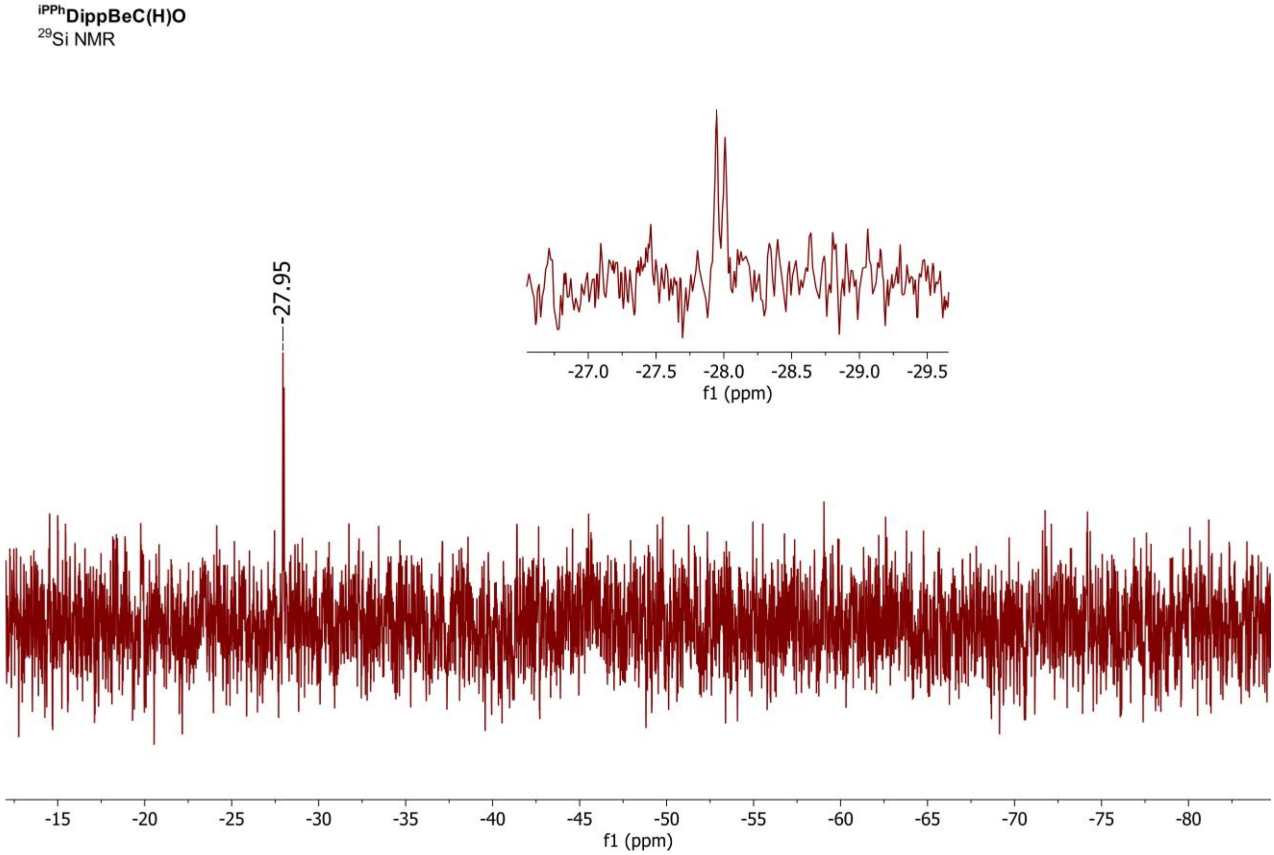

Supplementary Figure 53. ^{29}Si{^{1}H} NMR spectrum of [^{iPPh}DippBeC(H)O]$_2$ in D$_8$-THF at ambient temperature.

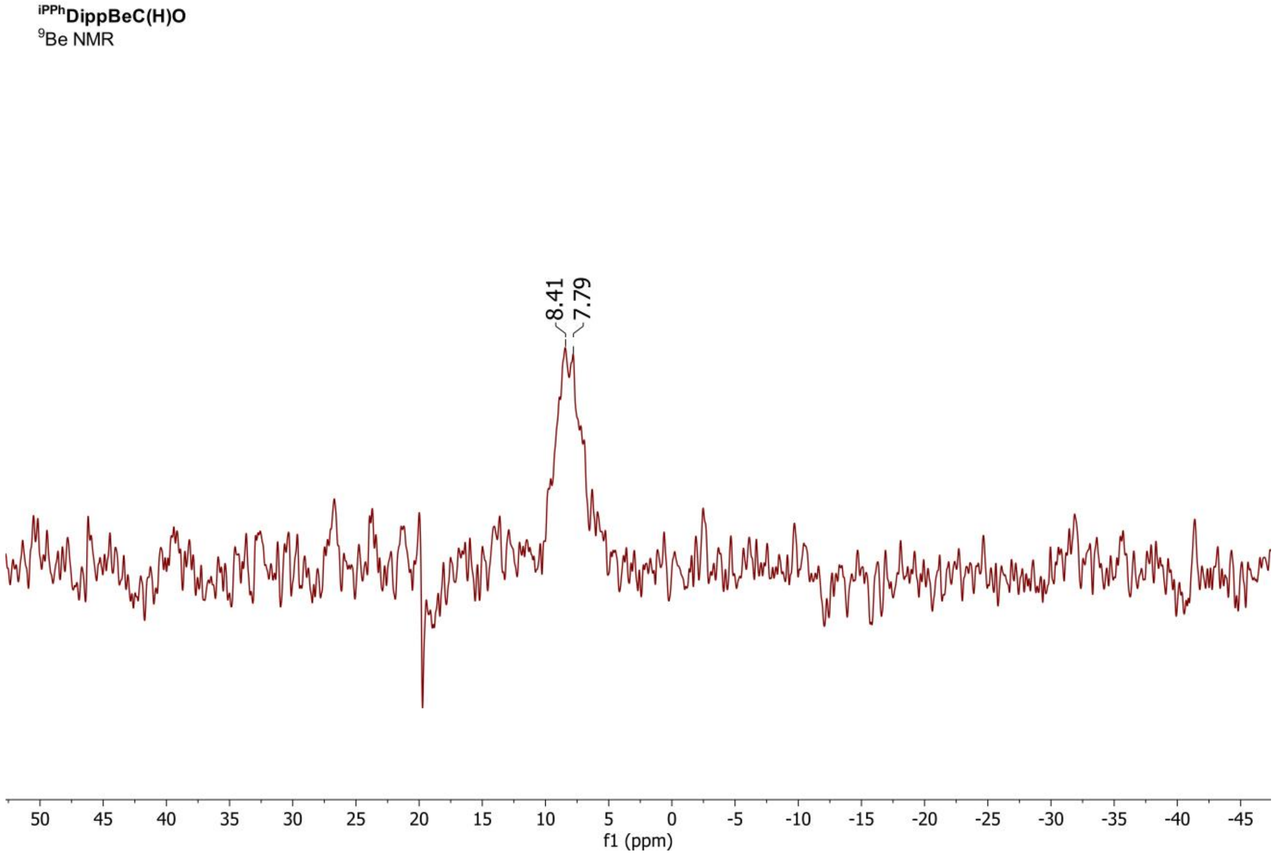

Supplementary Figure 54. ^{9}Be NMR spectrum of [^{iPPh}DippBeC(H)O]$_2$ in D$_8$-THF at ambient temperature.

IR Spectra

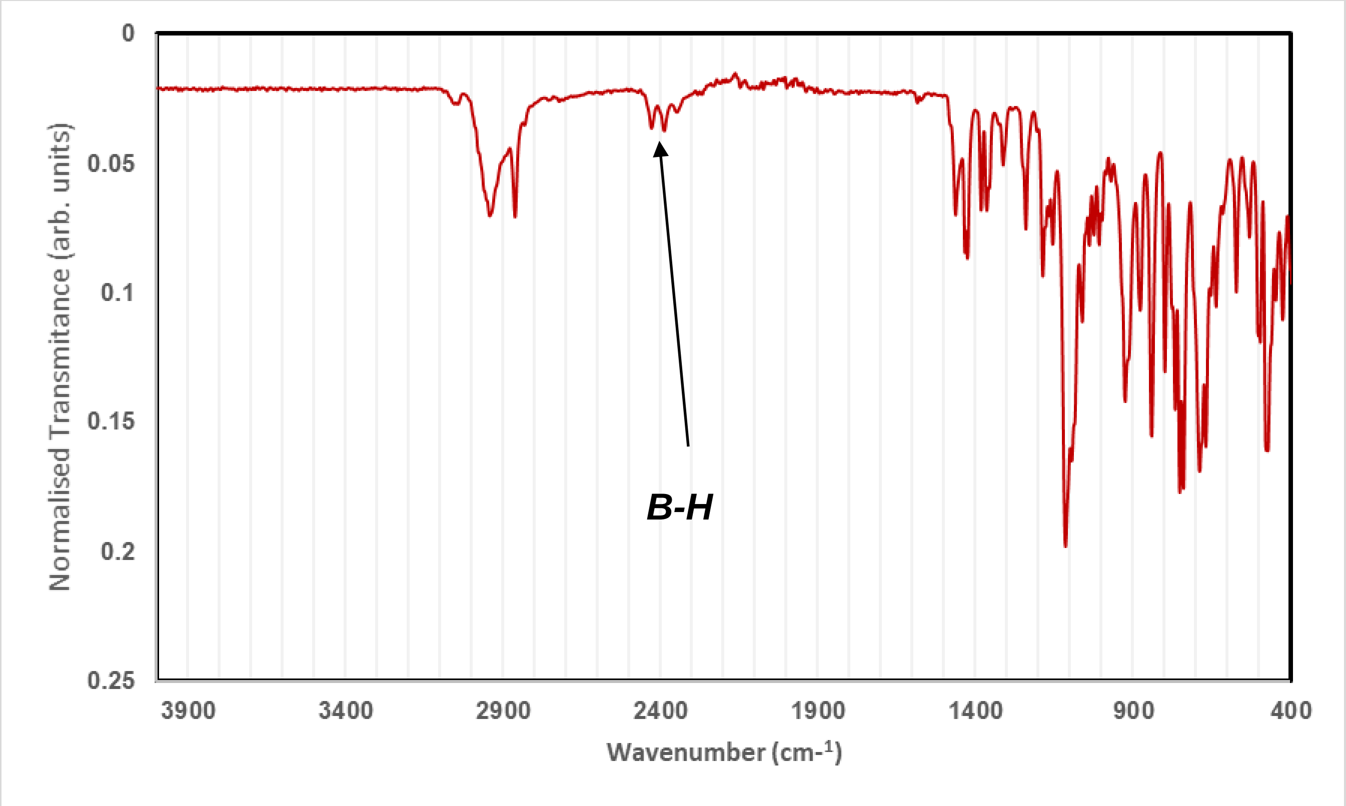

Supplementary Figure 55. ATR IR spectrum of ^{PhiP}DippBe(pin)BH_2, **5.**

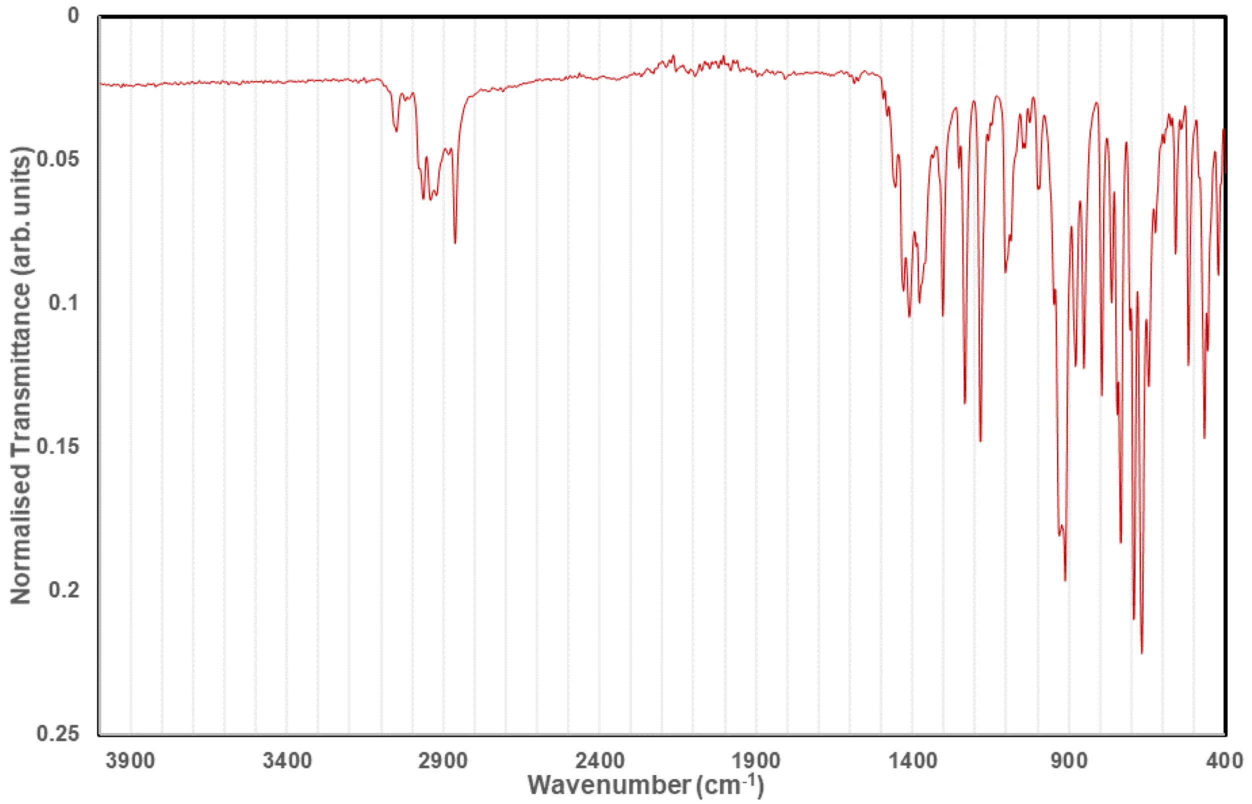

Supplementary Figure 56. ATR IR spectrum of [^{PhiP}Dipp-μ$_2$-BeH]$_2$, **6.**

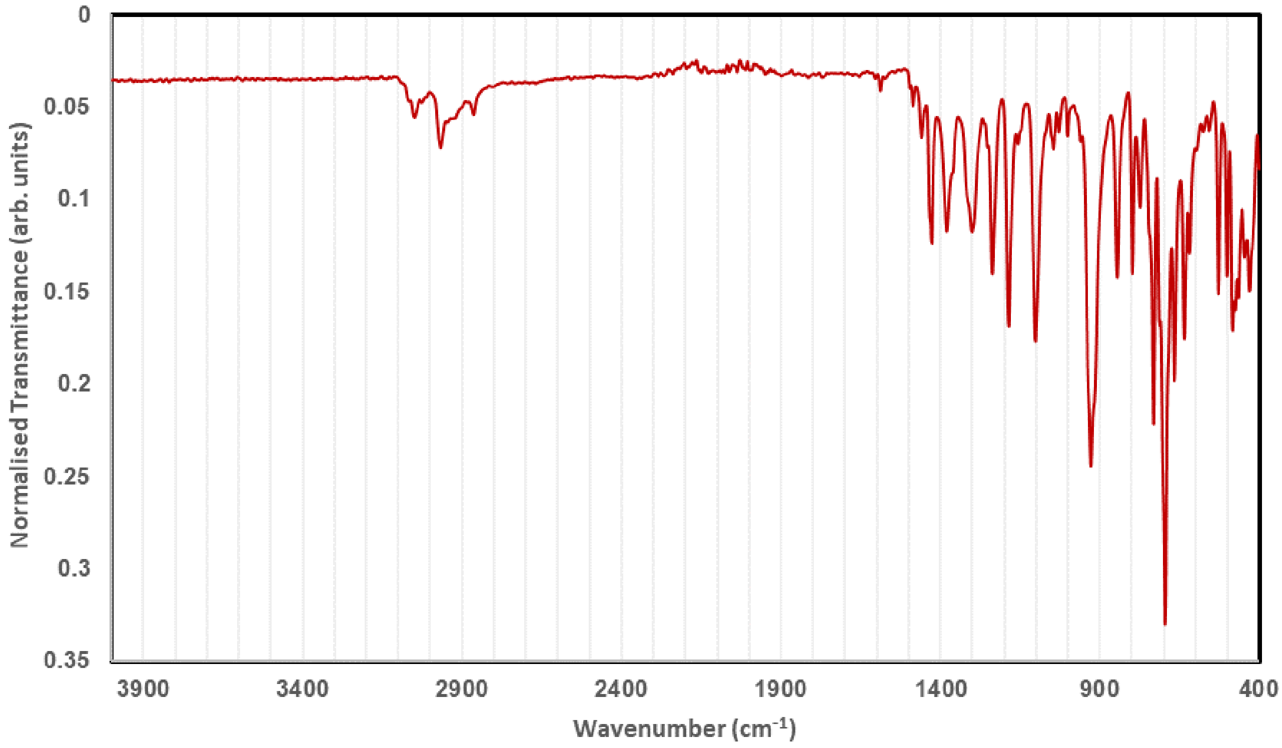

Supplementary Figure 57. ATR IR spectrum of $[^{PhPh}Dipp\text{-}\mu_2\text{-}BeH]_2$, **7.**

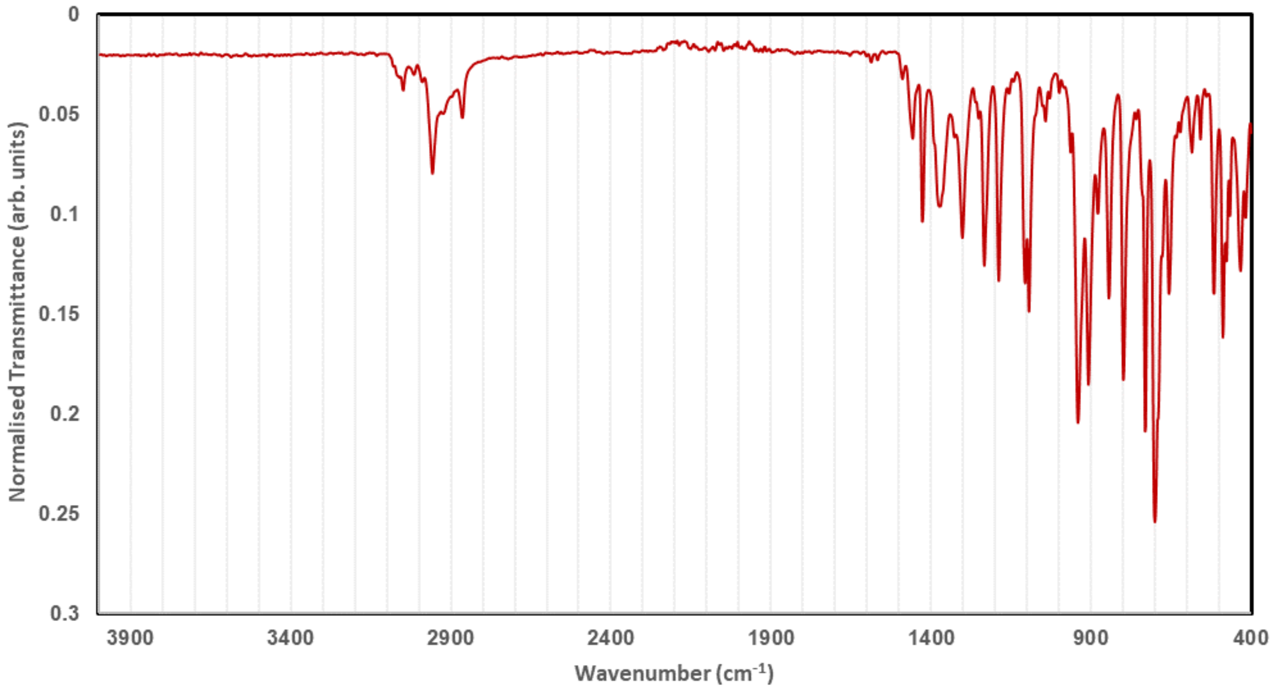

Supplementary Figure 58. ATR IR spectrum of $[^{iPPh}Dipp\text{-}\mu_2\text{-}BeH]_2$, **8.**

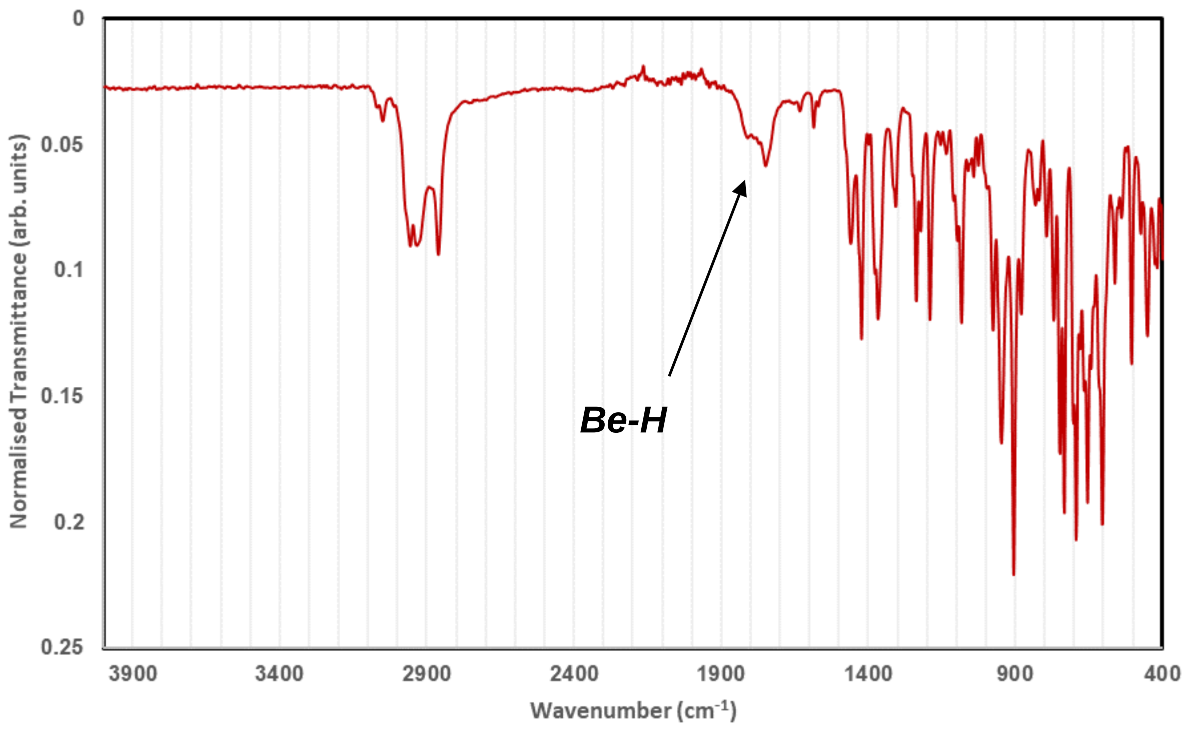

Supplementary Figure 59. ATR IR spectrum of ^{PhiP}DippBeH·NHC, **9.**

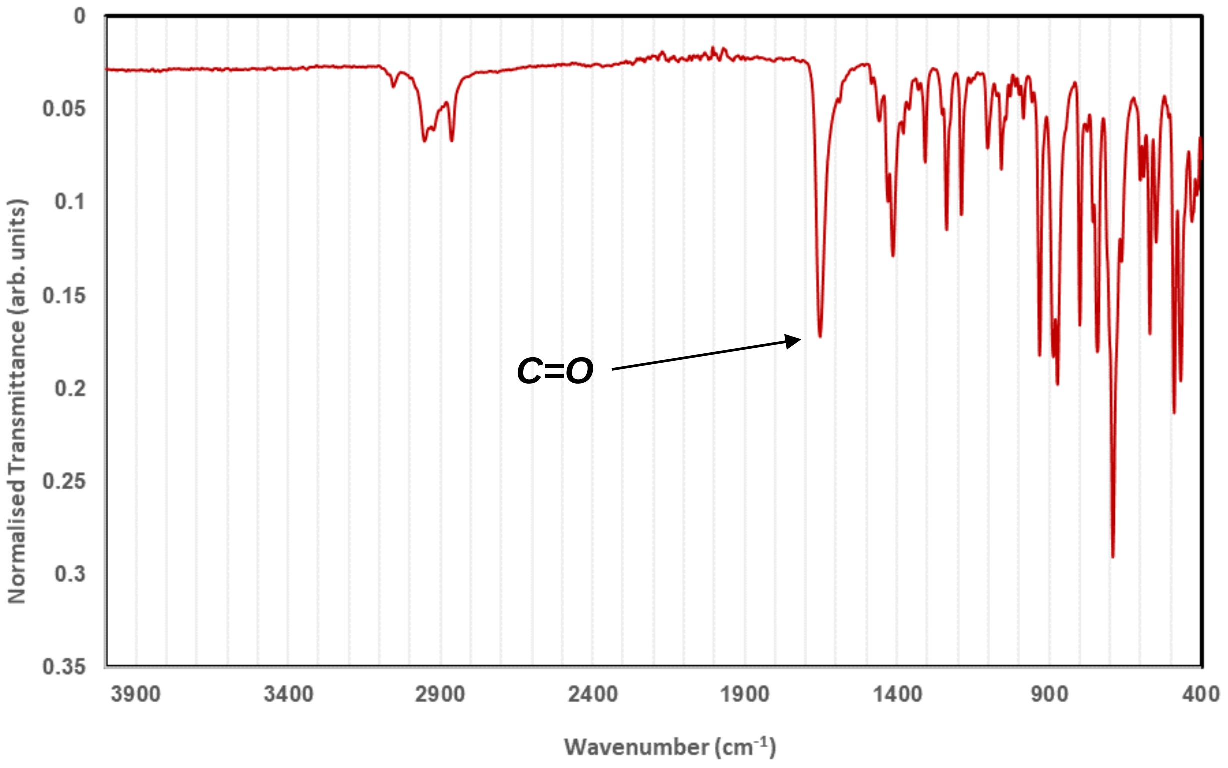

Supplementary Figure 60. ATR IR spectrum of [^{PhiP}DippBe-μ_2-{OC(H)O}]$_2$, **10.**

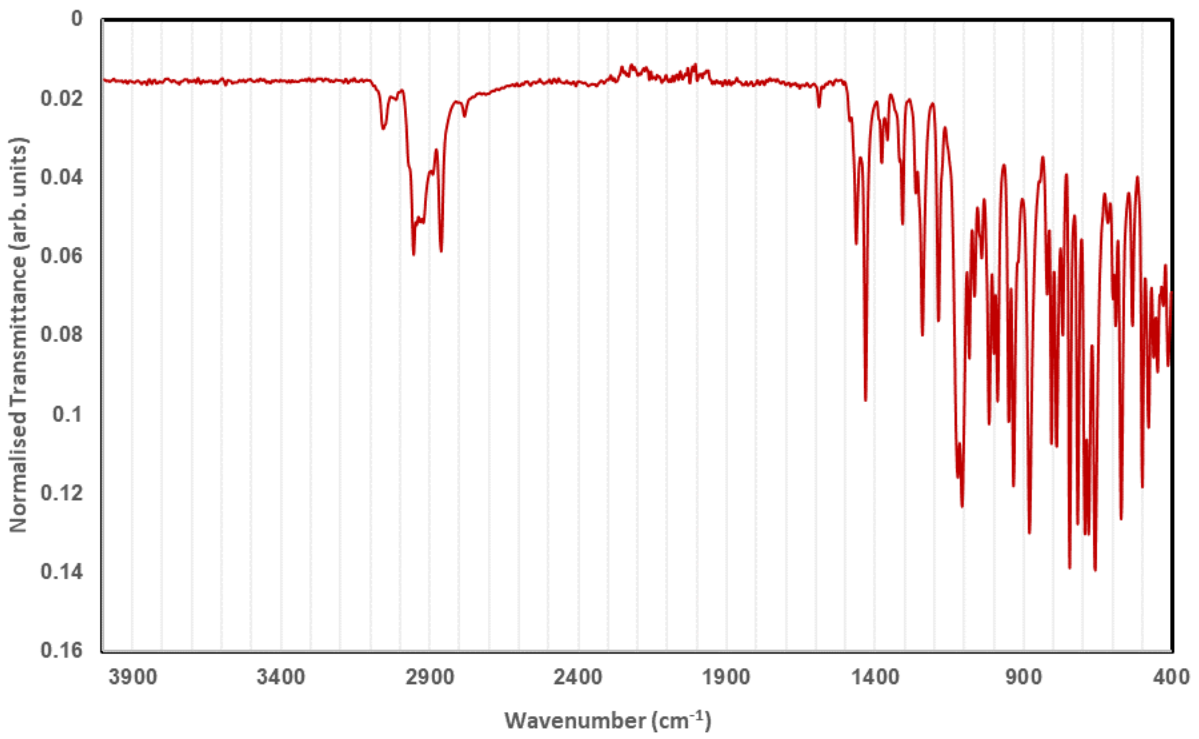

Supplementary Figure 61. ATR IR spectrum of [^{PhiP}DippBeC(H)O]$_2$, **11.**

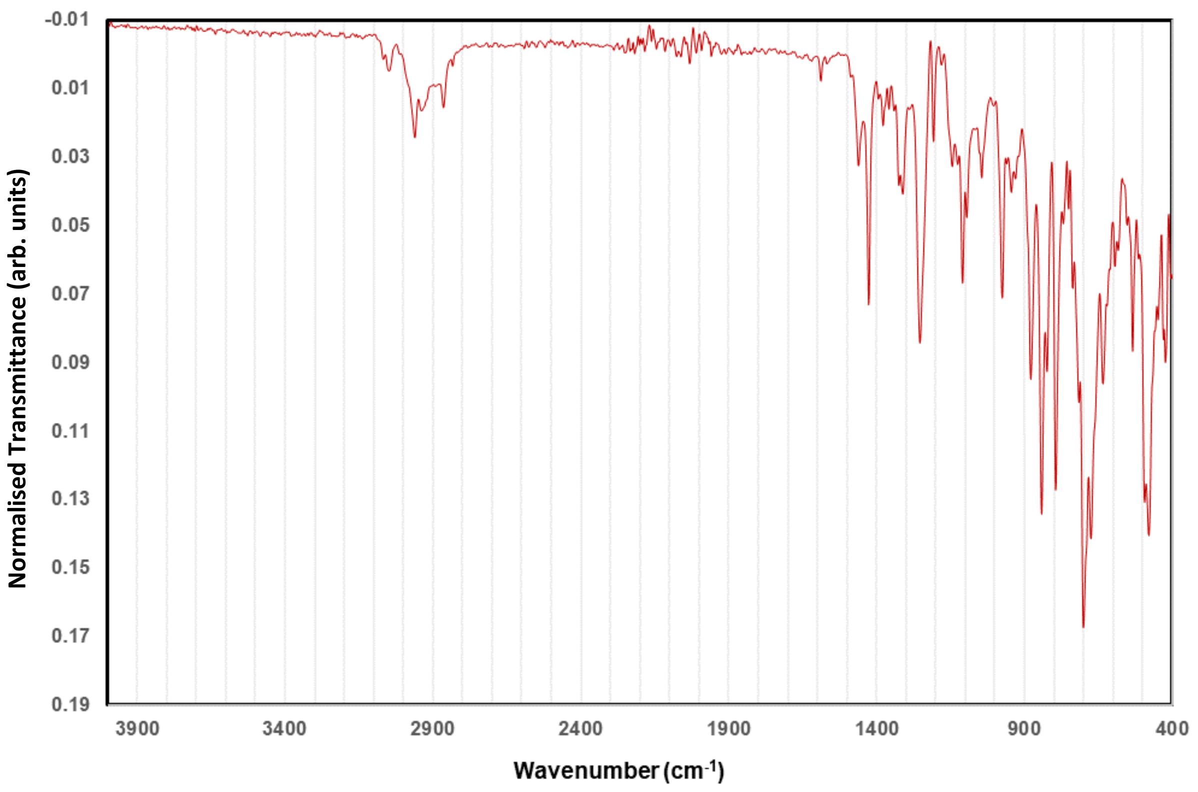

Supplementary Figure 62. ATR IR spectrum of [^{iPPh}DippBeC(H)O]$_2$, **12.**

Mass Spectra:

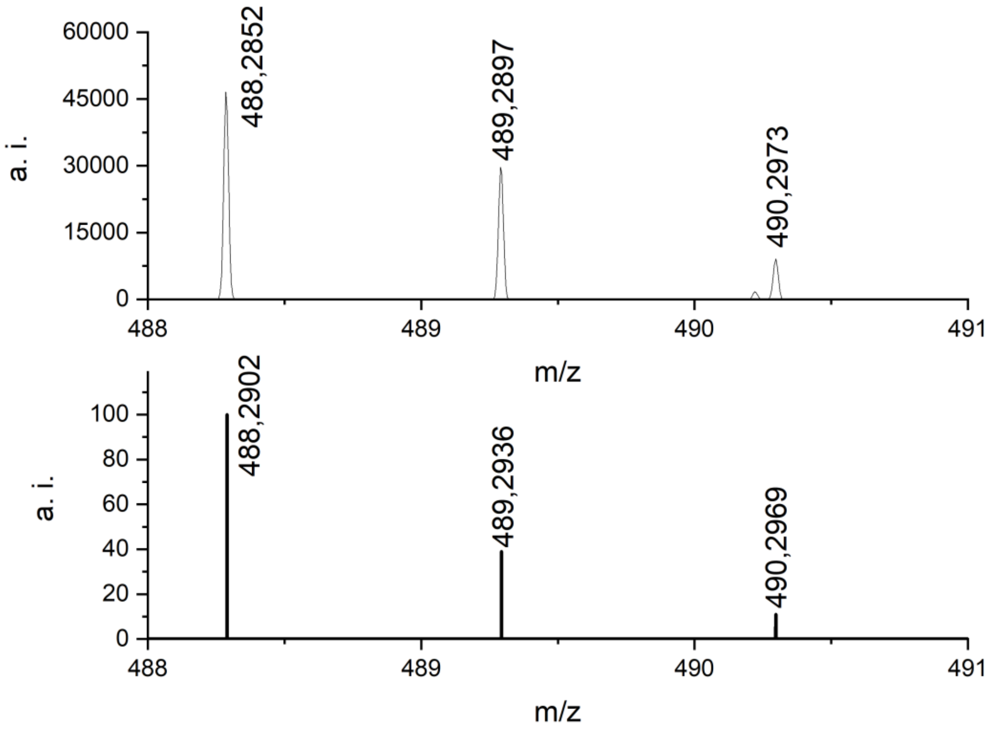

Supplementary Figure 63. LIFDI high-resolution mass spectrum of ^{iPPh}DippK.

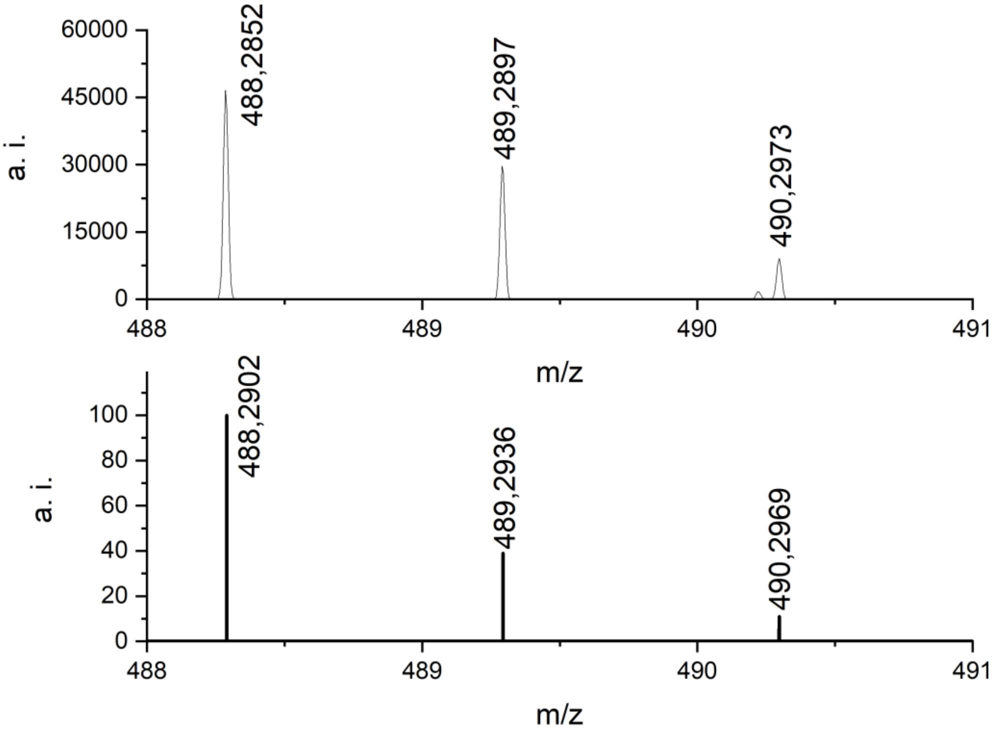

Supplementary Figure 64. LIFDI high-resolution mass spectrum of ^{PhiP}DippBeBr.

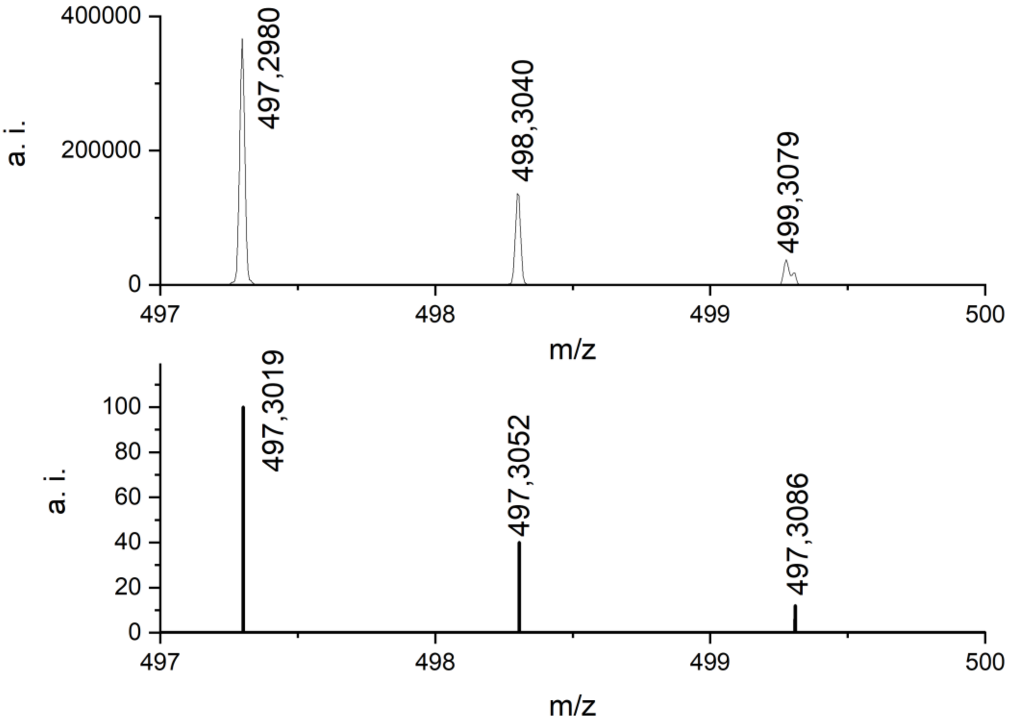

Supplementary Figure 65. LIFDI high-resolution mass spectrum of ^{PhiP}DippBeBz.

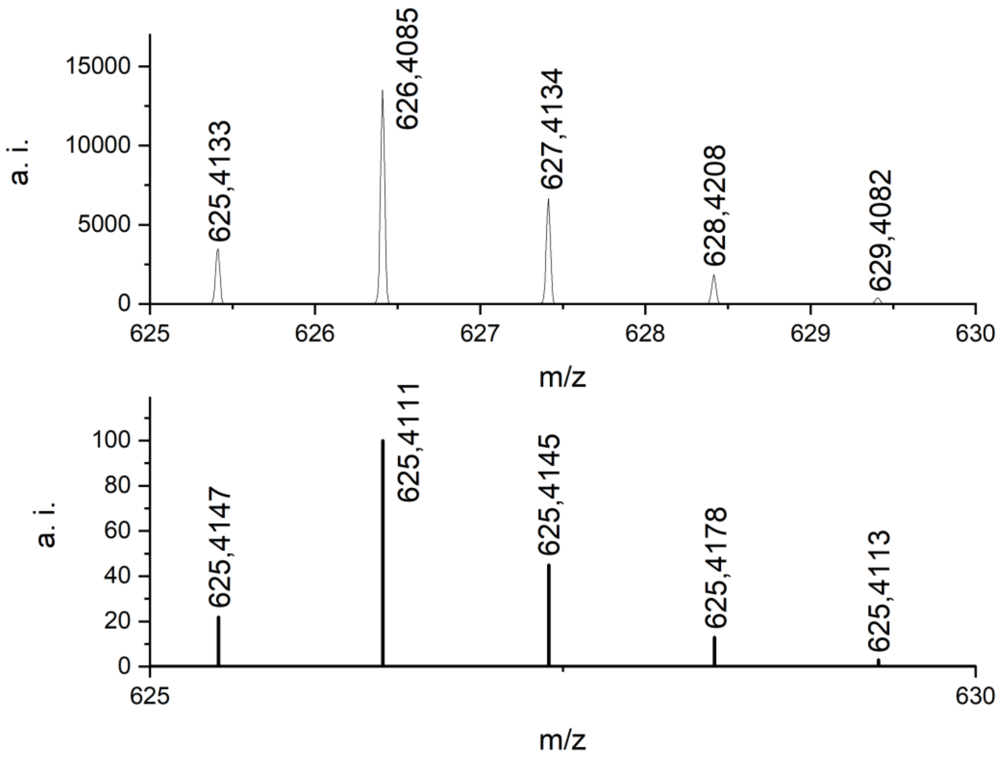

Supplementary Figure 66. LIFDI high-resolution mass spectrum of ^{PhiP}DippBe(pin)BH_2.

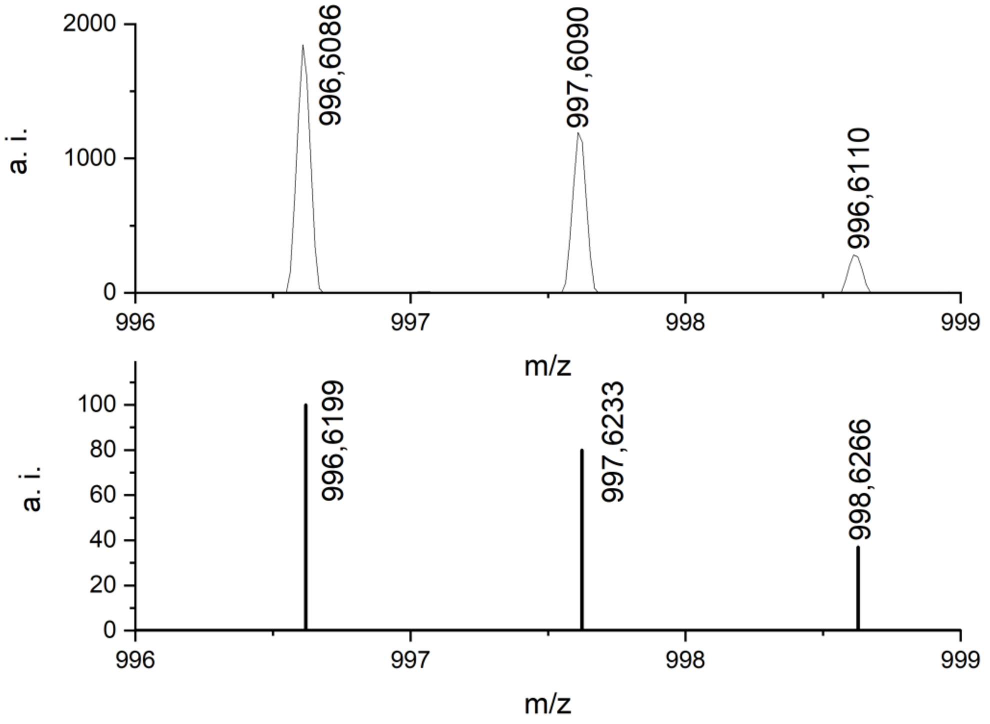

Supplementary Figure 67. LIFDI high-resolution mass spectrum of $[^{PhiP}DippNBeH]_2$.

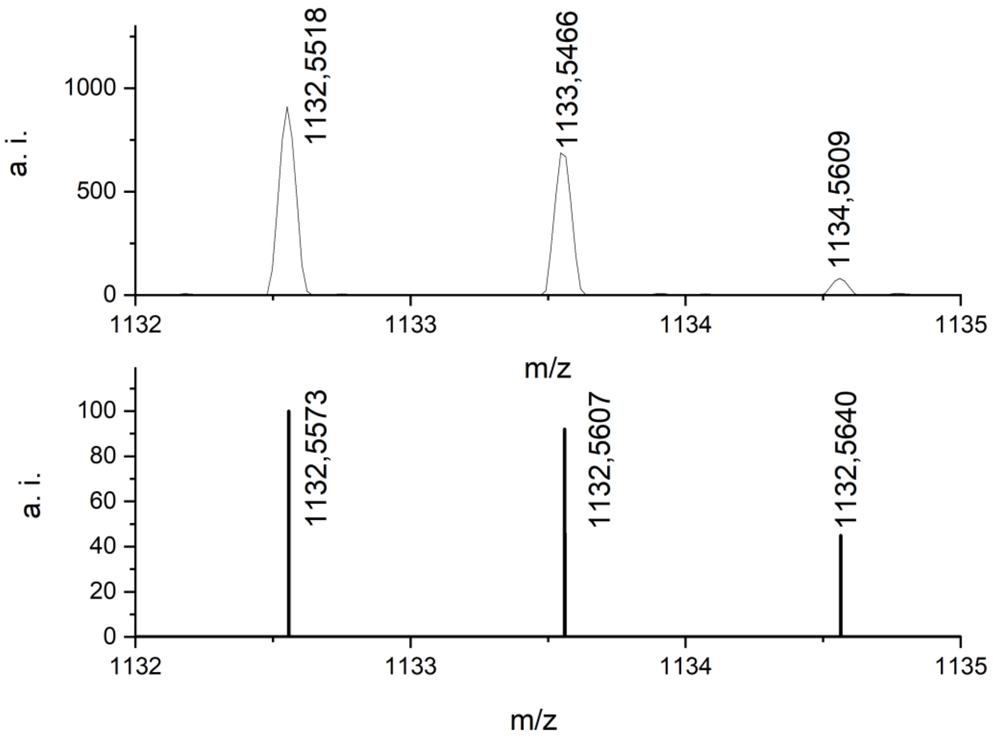

Supplementary Figure 68. LIFDI high-resolution mass spectrum of $[^{PhPh}DippBeH]_2$.

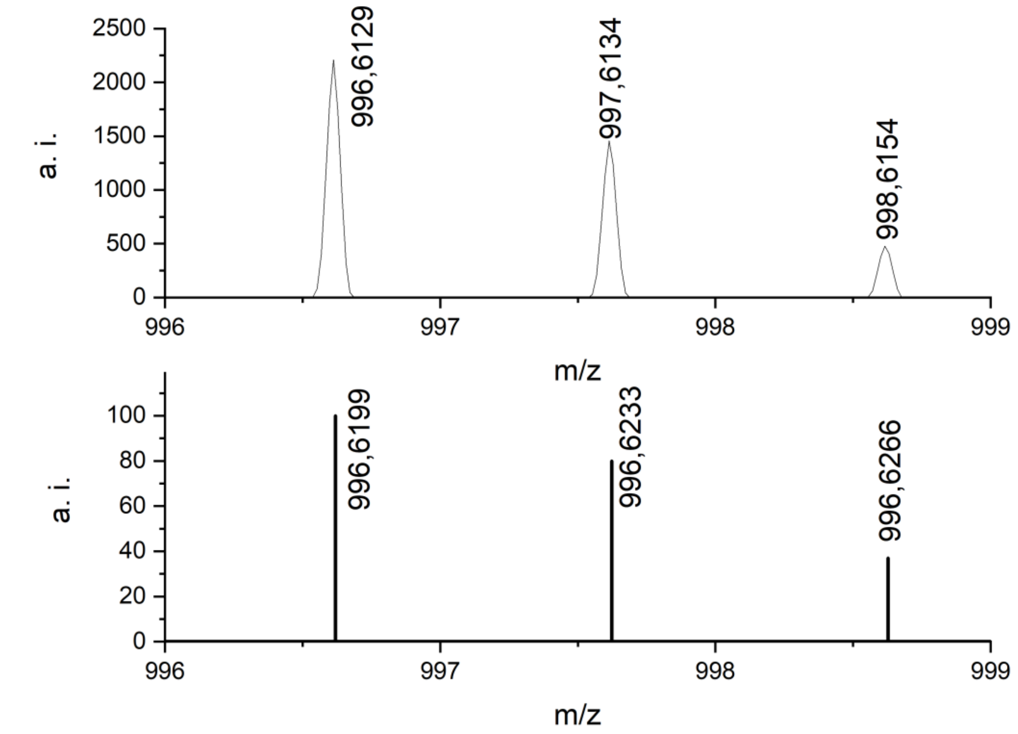

Supplementary Figure 69. LIFDI high-resolution mass spectrum of $[^{iPPh}DippBeH]_2$.

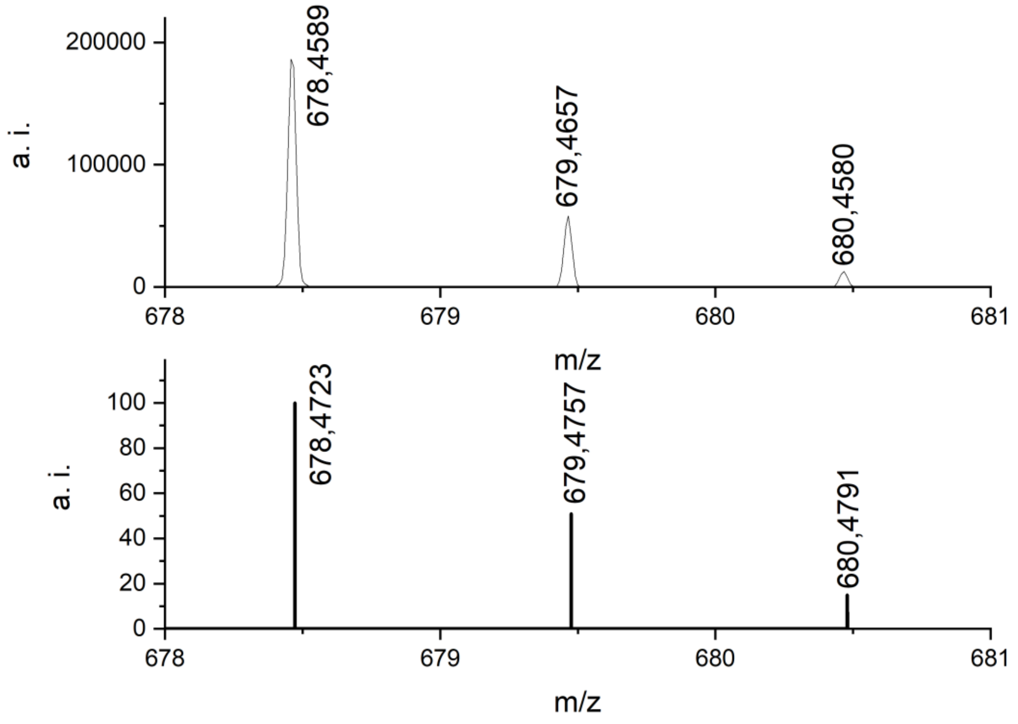

Supplementary Figure 70. LIFDI high-resolution mass spectrum of ^{PhiP}DippBeH·NHC.

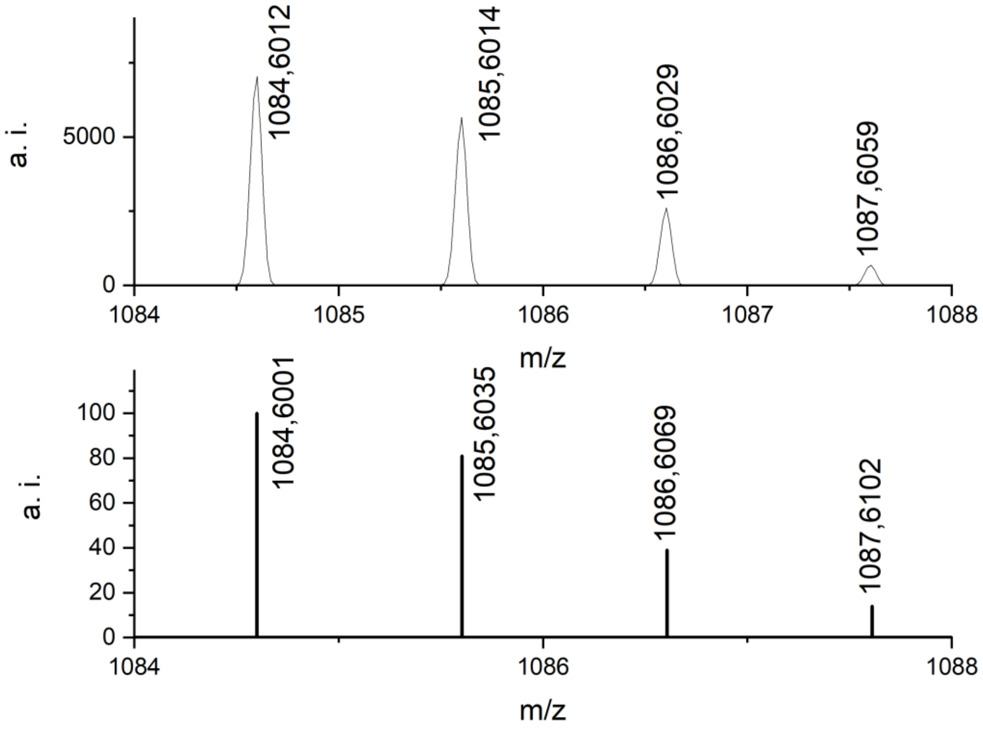

Supplementary Figure 71. LIFDI high-resolution mass spectrum of [^{PhiP}DippBeOC(H)O]$_2$.

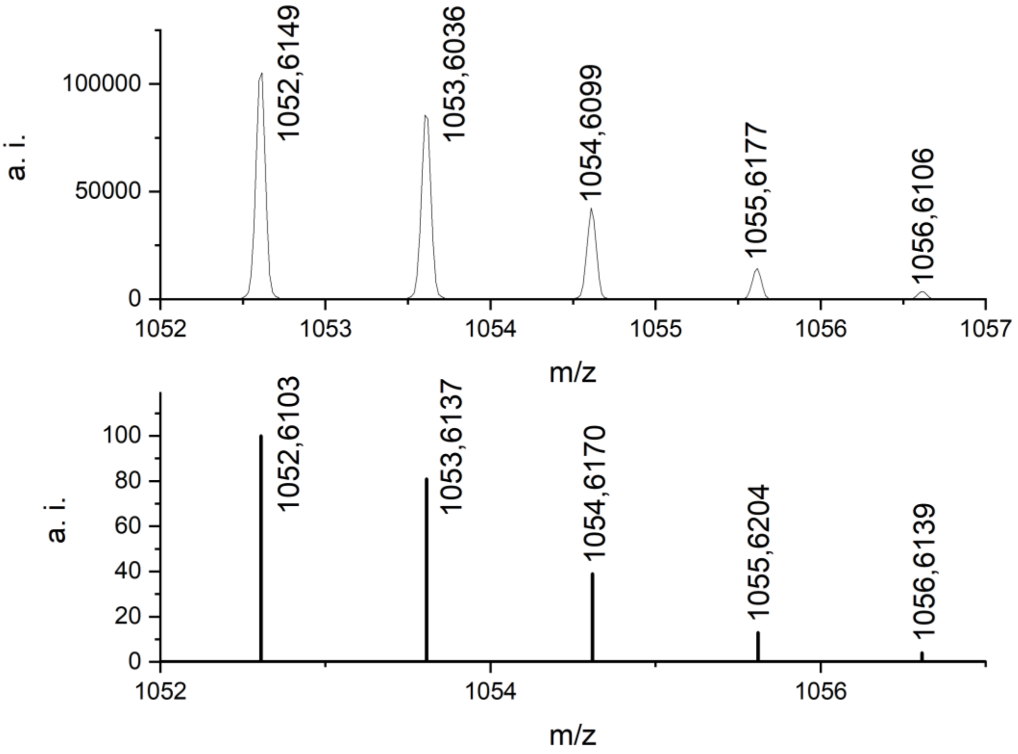

Supplementary Figure 72. LIFDI high-resolution mass spectrum of [^{PhiP}DippBeC(H)O]$_2$.

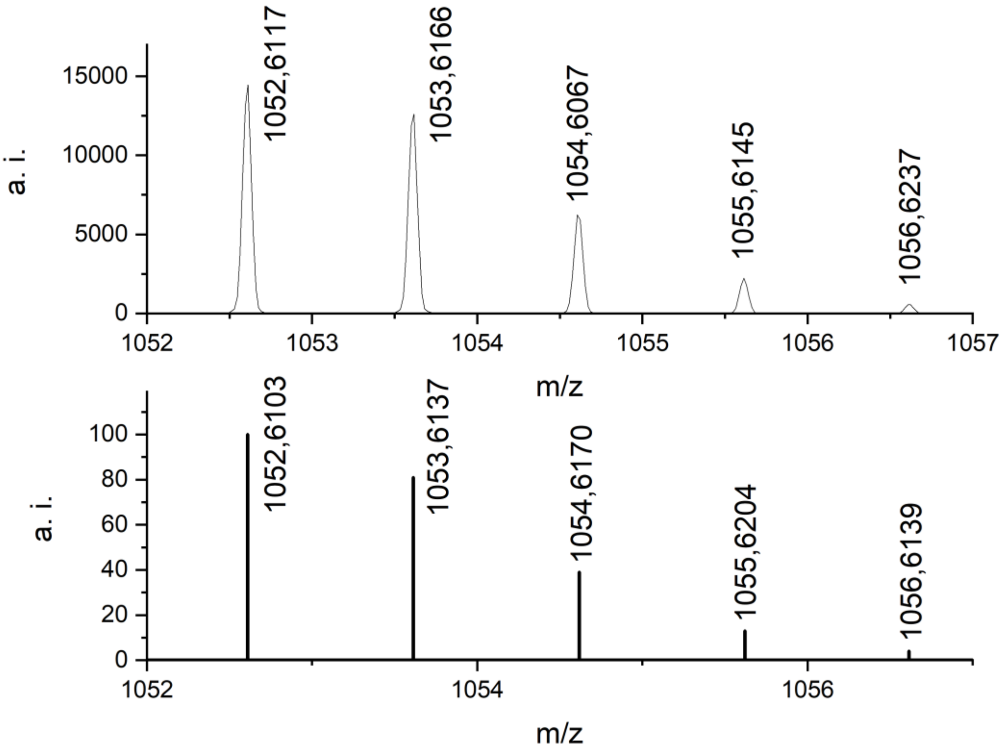

Supplementary Figure 73. LIFDI high-resolution mass spectrum of [^{iPPh}DippBeC(H)O]$_2$.

2. Single-Crystal X-Ray Crystallographic details

Single crystals of **4**-**12** suitable for X-ray structural analysis were mounted in perfluoroalkyl ether oil on a nylon loop and positioned in a 150 K cold N_2 gas stream. Data collection was performed with a STOE StadiVari diffractometer (MoKα radiation) equipped with a DECTRIS PILATUS 300K detector. Structures were solved by Direct Methods (SHELXS-97)[9] and refined by full-matrix least-squares calculations against F^2 (SHELXL-2018).[10] The positions of the hydrogen atoms were calculated and refined using a riding model, aside from hydride ligands in compounds **5**-**9**. All non-hydrogen atoms were treated with anisotropic displacement parameters. Crystal data, details of data collections, and refinements for all structures can be found in their CIF files, which are available free of charge via www.ccdc.cam.ac.uk/data_request/cif, and are summarized in Tables S1-S2.

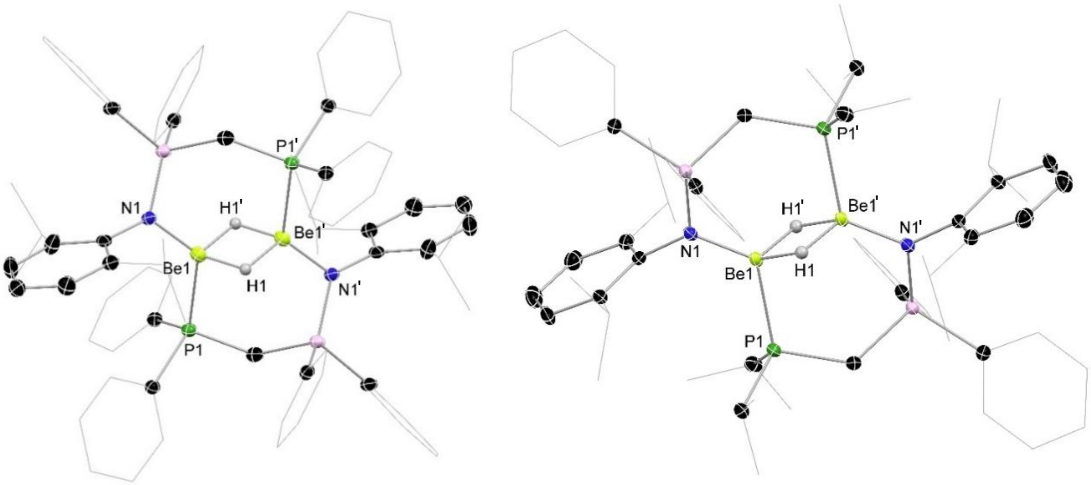

Supplementary Figure 74. The molecular structures of **7** ([^{PhPh}DippBe-μ$_2$-H]$_2$) *(left)* and **8** ([^{iPPh}Dipp-μ$_2$-BeH]$_2$) (*right*) with hydrogen atoms (aside from Be-*H* ligands) omitted and thermal ellipsoids at 30% probability. Selected bond lengths (Å) and angles (°) for **7**: Be1-P1 2.235(6); Be1-N1 1.634(5); Be1-Be1' 2.142(8); Be1-H1 1.42(4); Be1-H1' 1.44(4); P1-Be1-Be1' 105.0(3); N1-Be1-Be1' 129.8(3). For **8**: Be1-P1 2.225(5); Be1-N1 1.641(5); Be1-Be1' 2.133(8); Be1-H1 1.41(4); Be1-H1' 1.41(5); P1-Be1-Be1' 104.4(3); N1-Be1-Be1' 130.9(4).

Supplementary Table 1. Summary of X-ray data for compounds **4-8**.

	**4**	**5**	**6**	**7·toluene**	**8**
empirical form.	$C_{38}H_{50}BeNPSi$	$C_{37}H_{57}BBeNO_2PSi$	$C_{62}H_{88}Be_2N_2P_2Si_2$	$C_{88}H_{96}Be_2N_2P_2Si_2$	$C_{62}H_{88}Be_2N_2P_2Si_2$
formula wt	588.86	624.70	997.48	1317.80	997.48
crystal syst.	triclinic	monoclinic	triclinic	triclinic	triclinic
space group	*P-1*	$P2_1/n$	*P-1*	*P-1*	*P-1*
a (Å)	9.400(19)	16.530(3)	11.480(2)	11.300(2)	10.950(2)
b (Å)	9.820(2)	11.180(2)	12.140(2)	12.990(3)	12.200(2)
c (Å)	21.970(4)	21.500(4)	12.990(3)	13.300(3)	13.230(3)
α (deg.)	79.40(3)	90	95.50(3)	84.90(3)	108.50(3)
β (deg.)	88.60(3)	111.70(3)	108.40(3)	77.00(3)	97.70(3)
γ (deg.)	62.30(3)	90	117.10(3)	81.40(3)	112.30(3)
vol (Å^3)	1760.6(8)	3691.7(15)	1466.4(7)	1877.7(7)	1484.1(6)
Z	2	4	1	1	1
ρ(calc) (g.cm^{-3})	1.111	1.128	1.130	1.165	1.116
μ (mm^{-1})	0.138	0.138	0.154	0.136	0.152
F(000)	636	1360	540	704	540
T (K)	150(2)	150(2)	150(2)	150(2)	150(2)
reflns collect.	17932	25859	14622	22353	15507
unique reflns	6852	7189	5727	7330	5826
R_{int}	0.0312	0.0567	0.0620	0.0846	0.0836
R1 [$I>2\sigma(I)$]	0.0423	0.0473	0.0694	0.0720	0.0706
*w*R2 (all data)	0.1186	0.1114	0.2397	0.1797	0.2419
CCDC No.	2101682	2101683	2101684	2101685	2101686

Supplementary Table 2. Summary of X-ray data for compounds **9-12**.

	**9·(pentane)$_{0.33}$**	**10**	**11**	**12**
empirical form.	$C_{43.67}H_{68}BeN_3PSi$	$C_{64}H_{88}Be_2N_2O_4P_2Si_2$	$C_{64}H_{88}Be_2N_2O_2P_2Si_2$	$C_{64}H_{88}Be_2N_2O_2P_2Si_2$
formula wt	703.08	1085.50	1053.50	1053.50
crystal syst.	triclinic	monoclinic	triclinic	monoclinic
space group	*P-1*	$P2_1/c$	*P-1*	$P2_1$
a (Å)	13.770(3)	13.610(3)	10.910(2)	11.460(2)
b (Å)	21.150(4)	14.870(3)	12.250(3)	14.610(3)
c (Å)	23.160(5)	16.440(3)	12.490(3)	18.380(4)
α (deg.)	82.90(3)	90	97.30(3)	90
β (deg)	84.80(3)	71.00(3)	104.80(3)	102.50(3)
γ (deg.)	83.30(3)	90	108.10(3)	90
vol (Å^3)	6628(2)	3145.9(12)	1494.8(6)	3004.4(11)
Z	6	2	1	2
ρ(calc) (g.cm^{-3})	1.057	1.146	1.170	1.165
μ (mm^{-1})	0.120	0.153	0.157	0.156
F(000)	2304	1168	568	1136
T (K)	150(2)	150(2)	150(2)	150(2)
reflns collect.	25941	17171	21262	36122
unique reflns	13471	6128	6805	11534
R_{int}	0.0733	0.0495	0.0661	0.0622
R1 [I>2σ(I)]	0.0585	0.0461	0.0594	0.0490
*w*R2 (all data)	0.1630	0.1198	0.1458	0.0916
CCDC No.	2101687	2101688	2101689	2101690

3. Power X-ray diffraction details

For powder X-ray diffraction (PXRD) measurements, ~50 mg of crystalline compound were ground in an agate mortar, and a small amount (~2 mg) sealed inside 0.5 mm glass capillaries. PXRD measurements were performed at room temperature on a STOE Stadi P diffractometer equipped with a Ge(111) monochromator for Cu $K_{\alpha 1}$ radiation (λ = 1.54056 Å) and a Dectris MYTHEN DCS 1K solid-state detector. The raw powder data were processed with the software package WinXPOW.[11] Unit cell parameters were determined with the program Index and Refine from the software package WinXPOW. The experimentally obtained powder diffractograms are overlaid against the theoretical diffractograms generated from the single-crystal data.

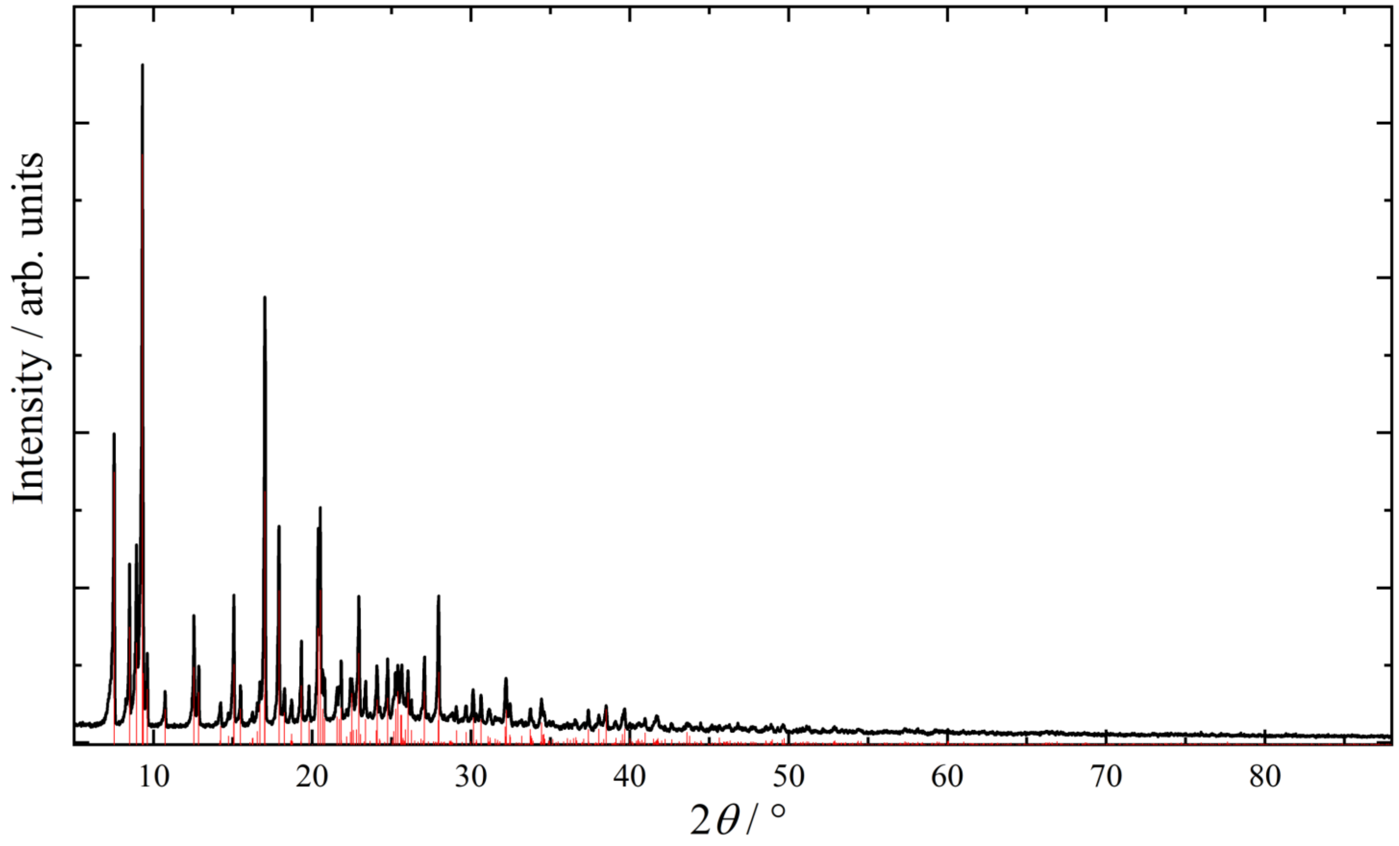

Supplementary Figure 75. Experimental powder X-ray diffraction pattern of $[^{PhiP}DippBe\text{-}\mu_2\text{-}H]_2$ (black). The red lines show the theoretical reflections obtained from the single crystal data. The lattice parameter for the theoretical diffractogram were obtained by indexing the main reflections of the experimental PXRD (a = 11.559(2) Å, b = 12.116(3) Å, c = 12.960(2) Å, α = 95.75(1)°, β = 108.69(1)°, γ = 116.19(1)°).

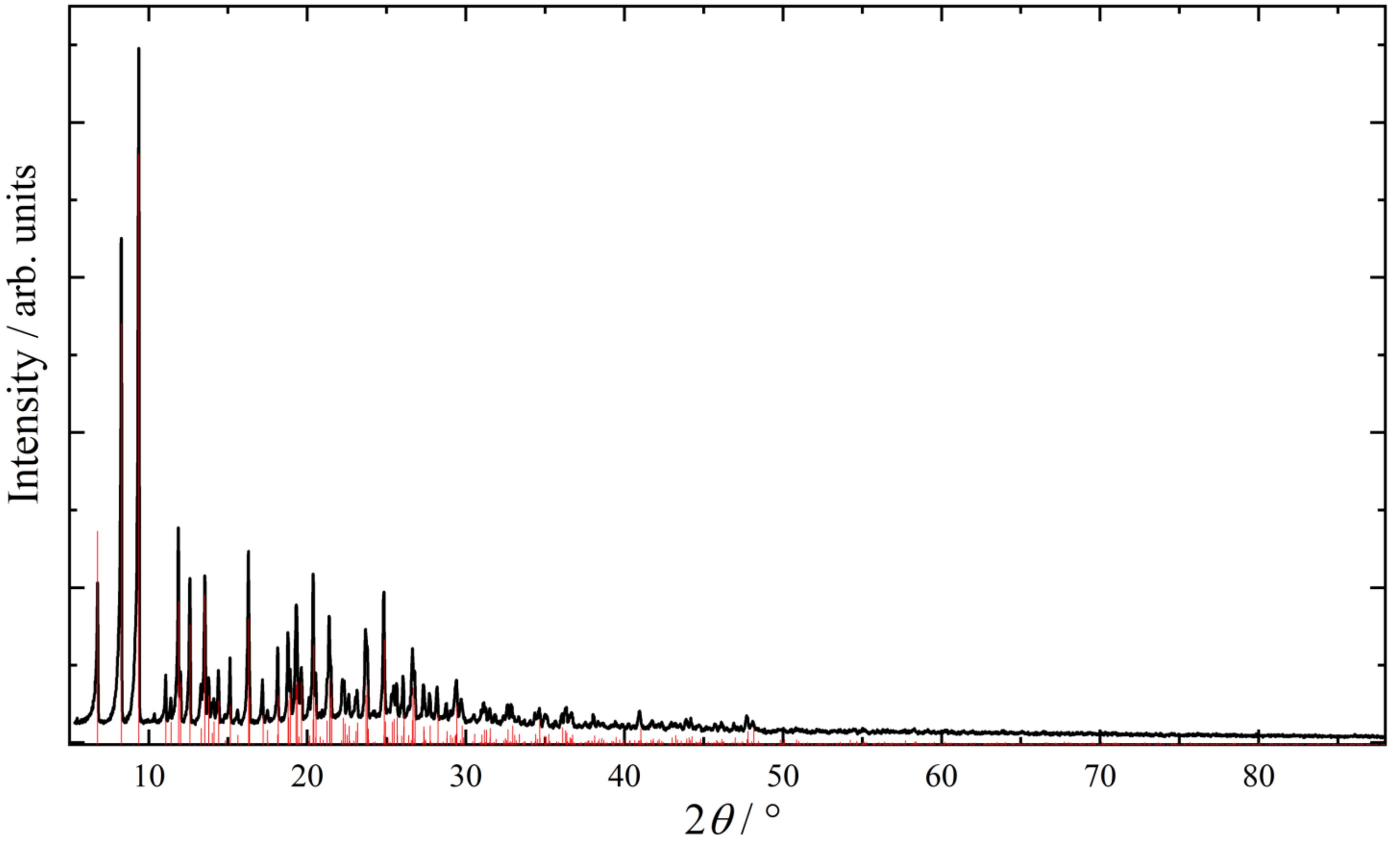

Supplementary Figure 76. Experimental powder X-ray diffraction pattern of [^{PhiP}DippBe-μ_2-{OC(H)O}]$_2$ (black). The red lines show the theoretical reflection obtained from the single crystal data. The lattice parameter for the theoretical diffractogram were obtained by indexing the main reflections of the experimental PXRD (*a* = 13.95(1) Å, *b* = 14.74(1) Å, *c* = 16.52(1) Å, α = *γ* = *90°*, *β* = 69.89(4)°).

4. Computational methods and details

DFT calculations were performed at the ωB97X-D3(SMD=benzene)/def2-TZVPP//B97-D3/def2-SVP level of theory.[12] Stationary points on the potential energy surface (PES) were characterized by harmonic vibrational frequency calculations. Transition states, which had one imaginary frequency, were analysed by intrinsic reaction coordinate (IRC) calculations to confirm the corresponding intermediates. Calculations were carried out using the GAUSSIAN 16 program suite.[13] QTIAM analysis was performed with Multiwfn 3.7.[14]

Supplementary Table 3. NBO analysis of the central $Be_2C_2O_2$ moiety in **11'**.

11	Occupation	Atom	Polarization	s-character	p-character	d-character
Bond	1.99	O	66.40%	35.51%	64.43%	0.05%
		C	33.60%	23.34%	76.44%	0.22%
Bond	1.99	O	66.40%	35.52%	64.43%	0.05%
		C	33.60%	23.34%	76.44%	0.22%
Lone Pair	1.95	O	-	38.20%	61.78%	0.02%
Lone Pair	1.89	O	-	25.17%	74.81%	0.02%
Lone Pair	1.87	O	-	1.16%	98.81%	0.03%
Lone Pair	1.95	O	-	38.20%	61.78%	0.02%
Lone Pair	1.89	O	-	25.19%	74.79%	0.02%
Lone Pair	1.87	O	-	1.13%	98.84%	0.03%
Lone Pair	1.78	C	-	28.40%	71.60%	0.01%
Lone Pair	1.78	C	-	28.40%	71.59%	0.01%
Empty orbital	0.25	Be	-	99.23%	0.77%	0.00%
Empty orbital	0.25	Be	-	99.23%	0.77%	0.00%

Supplementary Table 4. Calculated bond lengths [Å], NPA and CM5 charges, Wiberg Bond Index (WBI) and Mayer Bond Order (MBO) in the central $Be_2C_2O_2$ moiety in **11'**. Corresponding Electron Density, Laplacian of Electron Density, Kinetic Energy Density, Potential Energy Density, Total Electronic Energy Density, and Ellipticity are calculated at the Bond Critical Points of **11'**.

**Property**	**11'**	
**Bond length [Å]**	Be-C/Be-O/O-C	1.811/1.533/1.400
**NPA charge**	Be/C/O	+1.63/-0.77/-1.04
**CM5 charge**	Be/C/O	+0.63/-0.16/-0.49
**Wiberg Bond Index**	Be-C/Be-O/O-C	0.21/0.19/0.97
**Mayer Bond Order**	Be-C/Be-O/O-C	0.50/0.76/1.00
**Electron Density**	Be-C/Be-O	0.071/0.090
**Laplacian of Electron Density**	Be-C/Be-O	0.280/0.798
**Kinetic Energy Density**	Be-C/Be-O	0.086/0.182
**Potential Energy Density**	Be-C/Be-O	-0.103/-0.165
**Total Electronic Energy Density**	Be-C/Be-O	-0.016/-0.017
**Ellipticity**	Be-C/Be-O	0.13/0.04

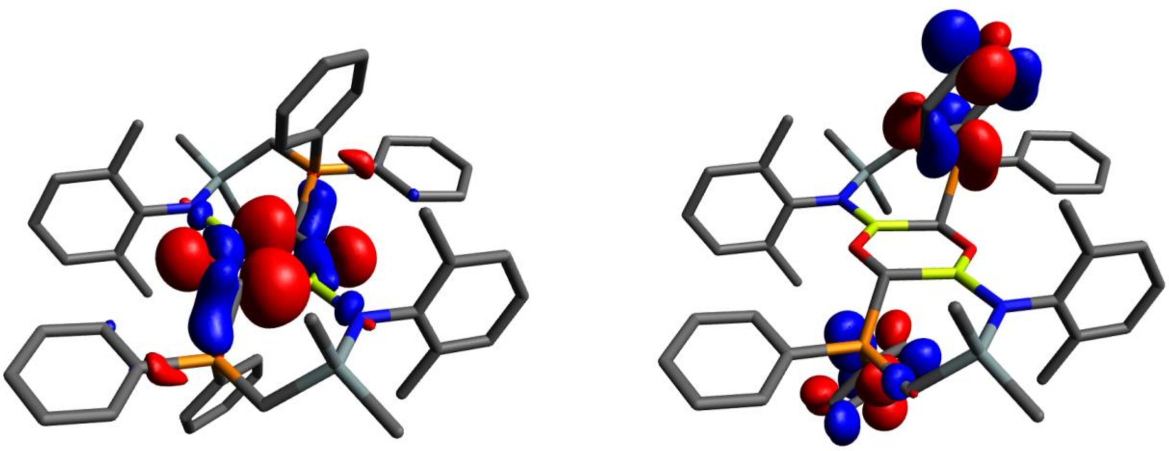

Supplementary Figure 77. HOMO (left, –4.04 eV) and LUMO (right, -1.79 eV) of **11’**.

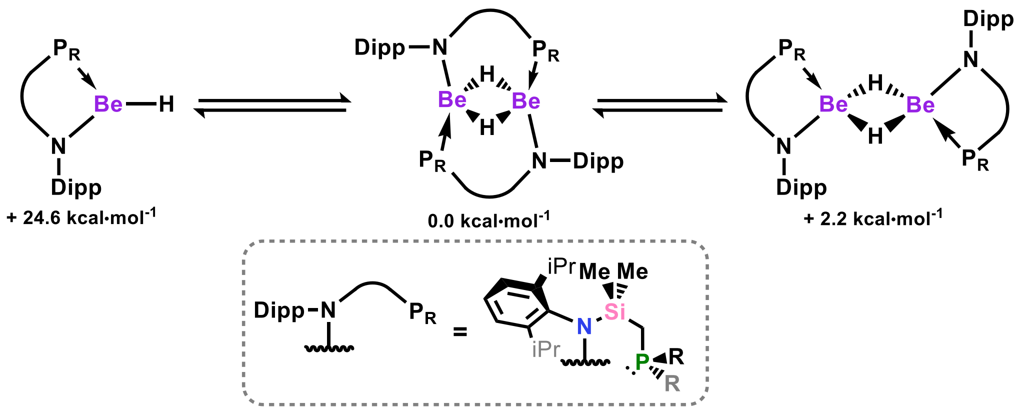

Supplementary Figure 78. Isomerisation energies of beryllium hydride complex **6’**.

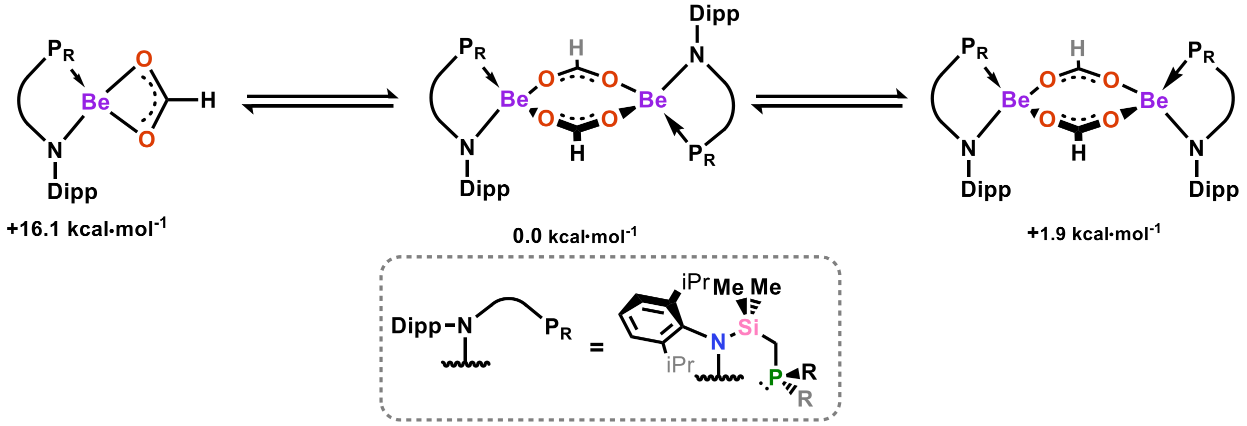

Supplementary Figure 79. Isomerisation energies of beryllium hydride complex **10**.

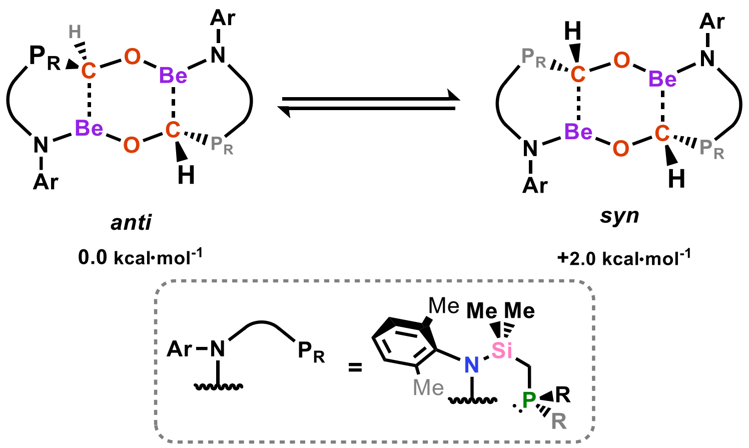

Supplementary Figure 80. Isomerisation energies of dimeric beryllium formyl complex **11’**.

Supplementary Table 5. Cartesian geometry of CO in Angstrom [Å].

Atomtype	X Coordinates	Y Coordinates	Z Coordinates
C	2.398477	0	-0.015239
O	1.412035	0	-0.584761

Supplementary Table 6. Cartesian geometry of **6’** in Angstrom [Å].

Atomtype	X Coordinates	Y Coordinates	Z Coordinates
P	2.247443	1.347144	-0.568376
Si	-0.4042	3.141332	0.065739
N	-1.546613	1.858681	-0.237818
C	3.117079	0.918832	-2.142387
C	-2.836172	2.081557	-0.775542
C	-3.105072	1.788923	-2.147951
C	-1.999942	1.254014	-3.028263
C	3.55078	1.922842	0.591276
C	-3.903484	2.543084	0.055034
C	4.546148	0.997361	0.984058
H	4.553814	-0.01778	0.572172
C	0.092201	3.258946	1.895348
C	5.5368	2.662404	2.459736
H	6.305121	2.949398	3.189236

C	-1.082495	4.794619	-0.566208
C	3.562056	3.220866	1.145581
H	2.799665	3.954015	0.863753
C	1.190915	2.80631	-0.996223
H	0.813712	2.575358	-2.010454
H	1.8295	3.705218	-1.099643
C	-5.190754	2.710428	-0.495082
H	-6.006014	3.056571	0.155546
C	5.533777	1.369691	1.907503
H	6.290148	0.632066	2.201805
C	-4.401604	1.975951	-2.663021
H	-4.594056	1.744337	-3.719664
C	4.487561	1.146583	-2.377165
H	5.105223	1.625691	-1.608119
C	-3.65677	2.817308	1.520374
C	4.549219	3.5852	2.0769
H	4.543069	4.596164	2.50439
C	2.920344	-0.09552	-4.356598
H	2.306202	-0.586881	-5.122106
C	2.33695	0.290111	-3.140796
H	1.274976	0.083022	-2.952218
C	4.289785	0.128871	-4.584354
H	4.749033	-0.183076	-5.531136
C	-5.446274	2.435723	-1.847102
H	-6.454017	2.573882	-2.260163
Be	0.972948	-0.353827	0.014567
C	5.069409	0.748339	-3.593393
H	6.139078	0.924978	-3.765526
H	0.072758	0.275087	1.032917
P	-2.247311	-1.347173	0.567874
Si	0.404229	-3.141436	-0.066596
N	1.546555	-1.858878	0.237658
C	-3.116584	-0.919045	2.142133
C	2.835749	-2.081769	0.776298

C	3.103707	-1.78941	2.148937
C	1.997919	-1.25502	3.028746
C	-3.550869	-1.922338	-0.591787
C	3.903682	-2.542961	-0.053696
C	-4.547015	-0.997116	-0.983197
H	-4.555209	0.017585	-0.570233
C	-0.091236	-3.258427	-1.896503
C	-5.53704	-2.661216	-2.46037
H	-6.305425	-2.947941	-3.189908
C	1.082153	-4.795002	0.56504
C	-3.561414	-3.219741	-1.147573
H	-2.798385	-3.952641	-0.866841
C	-1.191119	-2.806742	0.995121
H	-0.813887	-2.576357	2.009465
H	-1.829863	-3.705598	1.098024
C	5.190639	-2.71015	0.497187
H	6.006379	-3.05602	-0.152984
C	-5.534719	-1.36912	-1.906698
H	-6.291702	-0.631695	-2.19992
C	4.399978	-1.976252	2.664772
H	4.591733	-1.744793	3.721578
C	-4.486896	-1.147292	2.377447
H	-5.104689	-1.626604	1.608633
C	3.657946	-2.816865	-1.519256
C	-4.548647	-3.583728	-2.078948
H	-4.541927	-4.594205	-2.50758
C	-2.919314	0.095185	4.356354
H	-2.305018	0.586668	5.121661
C	-2.33627	-0.290173	3.140293
H	-1.274417	-0.08281	2.951344
C	-4.288594	-0.129645	4.584619
H	-4.747587	0.182095	5.531593
C	5.445256	-2.43562	1.849419
H	6.452776	-2.573617	2.263074

Be	-0.972698	0.353754	-0.014994
C	-5.068401	-0.749315	3.593921
H	-6.137943	-0.926325	3.766468
H	-0.07243	-0.275079	-1.033446
H	-0.811813	3.368307	2.52203
H	0.758857	4.119034	2.09624
H	0.614648	2.339408	2.216619
H	-4.6051	2.9111	2.078559
H	-3.086293	3.755015	1.670154
H	-3.055582	2.009606	1.972016
H	-2.340007	1.118416	-4.070009
H	-1.62902	0.280096	-2.653391
H	-1.124233	1.929381	-3.028939
H	-2.032251	5.053865	-0.062432
H	-1.293743	4.733367	-1.650673
H	-0.364101	5.61962	-0.397417
H	1.626657	-0.281311	2.653696
H	2.337458	-1.119281	4.070646
H	1.122566	-1.93086	3.029129
H	3.057595	-2.00874	-1.971262
H	3.086989	-3.754177	-1.669615
H	4.606657	-2.911142	-2.076705
H	-0.613301	-2.338687	-2.217817
H	-0.757791	-4.11838	-2.098275
H	0.813273	-3.367579	-2.522527
H	1.291527	-4.73475	1.649921
H	2.032846	-5.053435	0.062615
H	0.364349	-5.620092	0.394174

Supplementary Table 7. Cartesian geometry of TS1 (16.5 kcal/mol) in Figure 5 in Angstrom [Å].

Atomtype	X Coordinates	Y Coordinates	Z Coordinates
C	2.707145	-2.41716	2.755963
C	3.343539	-2.211273	1.510216

C	4.652561	-2.714468	1.32437
C	5.302412	-3.411286	2.357165
C	4.658518	-3.617889	3.587782
C	3.360633	-3.118961	3.781573
P	2.412439	-1.450055	0.079206
C	3.721871	-1.165729	-1.182272
C	4.635208	-0.113479	-0.939909
C	5.669911	0.143429	-1.849854
C	5.790199	-0.62318	-3.021465
C	4.865547	-1.647971	-3.283855
C	3.837079	-1.921288	-2.36748
C	1.447428	-2.942669	-0.461396
Si	-0.174423	-2.722499	-1.467155
C	-0.732102	-4.510009	-1.767072
N	-1.384304	-1.824172	-0.540695
C	-2.607673	-2.453643	-0.152137
C	-2.75064	-2.910236	1.19189
C	-3.962911	-3.490336	1.615027
C	-5.050756	-3.618961	0.742301
C	-4.918241	-3.173233	-0.580233
C	-3.715722	-2.605499	-1.049709
C	-1.611525	-2.74968	2.170296
C	-3.634078	-2.151496	-2.48369
C	0.251511	-1.818206	-3.078915
Be	-1.024759	-0.305182	-0.101686
Be	0.998103	0.599904	0.373565
N	1.189013	2.355713	0.386257
C	2.498417	2.7671	0.043843
C	2.944749	2.65824	-1.317939
C	4.249879	3.046923	-1.670096
C	5.153226	3.524558	-0.708287
C	4.736865	3.613356	0.625462
C	3.431997	3.249492	1.018663
C	2.006382	2.142018	-2.382869

C	3.037322	3.335368	2.474566
Si	-0.059681	3.406456	0.956427
C	-0.531542	3.332546	2.803015
C	-1.661032	2.998147	-0.027493
P	-2.479958	1.345481	-0.017038
C	-3.75228	1.261908	1.292107
C	-4.518215	0.075472	1.392699
C	-5.460368	-0.063226	2.422819
C	-5.640291	0.968232	3.362287
C	-4.885381	2.149187	3.260091
C	-3.944027	2.300755	2.22685
C	-3.396639	1.458188	-1.626116
C	-4.798195	1.547204	-1.721891
C	-5.413875	1.64477	-2.982379
C	-4.637661	1.666739	-4.152413
C	-3.236971	1.58343	-4.062516
C	-2.620603	1.467825	-2.808903
C	0.303381	5.219899	0.53014
O	1.471267	0.495533	2.156758
C	0.361368	0.106549	2.510909
H	4.536777	0.516081	-0.045454
H	6.597784	-0.415075	-3.734903
H	3.124605	-2.725344	-2.583779
H	1.12213	-3.385855	0.50113
H	2.127839	-3.690167	-0.912566
H	-5.763859	-3.268389	-1.275245
H	6.368193	0.963348	-1.647821
H	-4.051843	-3.835029	2.654535
H	5.171045	-2.566497	0.370737
H	4.945832	-2.242266	-4.203159
H	2.848358	-3.270787	4.740379
H	1.695794	-2.037443	2.939926
H	5.165213	-4.166279	4.392115
H	-5.991701	-4.066552	1.087977

H	6.318078	-3.79469	2.194266
H	0.067105	0.312756	-0.791226
H	-4.382843	-0.741423	0.672068
H	-6.366798	0.85019	4.176493
H	-3.361364	3.227216	2.157255
H	-1.350353	3.088005	-1.084837
H	-2.431815	3.777958	0.13622
H	5.44104	3.969721	1.3914
H	-6.041471	-0.99122	2.492234
H	4.564916	2.96099	-2.718816
H	-5.41349	1.537463	-0.814393
H	-5.023521	2.957882	3.989005
H	-2.622642	1.590043	-4.971708
H	-1.527891	1.369271	-2.748266
H	-5.12294	1.745004	-5.13359
H	6.172204	3.81702	-0.993914
H	-6.507736	1.709754	-3.046877
H	-0.283615	-0.073611	1.337042
H	-0.657669	-1.614257	-3.673342
H	0.949407	-2.412323	-3.700215
H	0.736689	-0.850327	-2.862982
H	-4.63399	-2.083893	-2.945831
H	-3.030772	-2.840042	-3.103383
H	-3.142666	-1.176402	-2.553672
H	-1.868185	-3.162356	3.161673
H	-1.342097	-1.689119	2.31073
H	-0.691893	-3.255495	1.815071
H	-1.683116	-4.581686	-2.319664
H	-0.879615	-5.019872	-0.79599
H	0.041259	-5.063986	-2.333337
H	1.74777	1.079392	-2.217735
H	2.457315	2.214884	-3.388439
H	1.044223	2.689396	-2.373653
H	2.529056	2.408663	2.79525

H	2.338665	4.173025	2.664721
H	3.919791	3.499661	3.119356
H	-0.994525	2.373551	3.111703
H	-1.267528	4.140716	2.991937
H	0.338207	3.512986	3.458662
H	0.438061	5.369538	-0.55775
H	1.231044	5.55901	1.029869
H	-0.527221	5.867417	0.871088

Supplementary Table 8. Cartesian geometry of IM1 (3.5 kcal/mol) in Figure 5 in Angstrom [Å].

Atomtype	X Coordinates	Y Coordinates	Z Coordinates
C	5.802447	1.14854	2.654096
C	4.984592	1.204689	1.512654
C	3.587771	1.347199	1.645205
C	3.025661	1.424079	2.940421
C	3.84584	1.371901	4.076628
C	5.238286	1.232886	3.937308
P	2.446677	1.30304	0.187009
C	1.440723	2.843103	0.379349
Si	-0.105642	3.191261	-0.742272
C	-0.673098	4.941758	-0.286387
C	3.564543	1.530035	-1.25923
C	4.102529	0.358184	-1.840428
C	4.979792	0.448841	-2.932592
C	5.316015	1.705877	-3.464397
C	4.779309	2.873576	-2.894331
C	3.912133	2.788135	-1.792503
N	-1.394882	2.054166	-0.419417
C	-2.63131	2.506169	0.107009
C	-2.799322	2.727285	1.506118
C	-4.040813	3.180461	1.99444
C	-5.125966	3.390475	1.13142
C	-4.974725	3.135691	-0.241629

C	-3.743374	2.69796	-0.767276
C	-1.660988	2.437701	2.458664
C	-3.57841	2.41474	-2.24193
C	0.490524	3.204821	-2.54578
Be	-1.11028	0.45658	-0.596472
Be	1.156063	-0.493969	-0.246066
N	1.442747	-2.032189	0.27366
C	2.694944	-2.58814	-0.06616
C	2.905449	-3.20015	-1.340927
C	4.209102	-3.567684	-1.730824
C	5.304509	-3.371025	-0.876018
C	5.089789	-2.83893	0.404637
C	3.801651	-2.461882	0.830605
C	1.729354	-3.480178	-2.246458
C	3.56901	-1.969655	2.23866
Si	0.116286	-2.81377	1.074543
C	-1.452751	-2.775809	-0.088064
P	-2.513756	-1.259688	-0.19196
C	-3.58179	-1.260391	1.307389
C	-4.446951	-0.160172	1.499697
C	-5.308669	-0.121509	2.606059
C	-5.319745	-1.177968	3.532594
C	-4.470715	-2.281186	3.341208
C	-3.609357	-2.326044	2.232304
C	-3.70616	-1.642504	-1.558615
C	-4.982583	-2.194422	-1.316162
C	-5.841267	-2.487881	-2.388383
C	-5.435454	-2.236657	-3.710131
C	-4.165414	-1.687785	-3.957048
C	-3.303825	-1.386894	-2.889728
C	-0.273196	-1.975723	2.734909
C	0.506611	-4.647584	1.364617
H	-4.456574	0.668348	0.784296
H	-5.989868	-1.143749	4.401331

H	-2.960787	-3.197567	2.098037
H	-1.054393	-2.89823	-1.113591
H	-2.109188	-3.649976	0.091039
H	5.935956	-2.713532	1.094613
H	-5.96308	0.748322	2.739683
H	4.362722	-4.020518	-2.720502
H	-5.310441	-2.388586	-0.287553
H	-4.477169	-3.113744	4.056486
H	-3.8444	-1.482342	-4.986435
H	-2.32069	-0.94413	-3.090291
H	-6.109824	-2.463765	-4.545866
H	6.316118	-3.649509	-1.198884
H	-6.833296	-2.913355	-2.188351
H	-0.089652	-0.023713	0.366261
H	3.839813	-0.629831	-1.440275
H	5.99372	1.776766	-4.324955
H	3.503344	3.707804	-1.358386
H	1.040125	2.781348	1.407751
H	2.11997	3.718247	0.367155
H	-5.825271	3.280889	-0.921973
H	5.392686	-0.469956	-3.367544
H	-4.16141	3.345764	3.074043
H	5.437543	1.128877	0.517225
H	5.036718	3.856854	-3.308741
H	3.39472	1.430877	5.075497
H	1.937804	1.502136	3.069646
H	5.87941	1.186743	4.826814
H	-6.089545	3.737437	1.526868
H	6.888081	1.03692	2.536153
C	0.748839	-0.259832	-1.989591
H	0.66241	-1.90102	3.320273
H	-1.014464	-2.531031	3.338187
H	-0.660103	-0.951652	2.58398
H	4.490707	-1.571446	2.696875

H	3.196506	-2.795078	2.879464
H	2.79552	-1.187523	2.257803
H	2.051629	-3.774648	-3.260991
H	1.058782	-2.607384	-2.324662
H	1.104422	-4.299161	-1.837914
H	1.425281	-4.745664	1.974137
H	0.683903	-5.178184	0.410414
H	-0.316138	-5.161008	1.898482
H	-1.101198	1.542619	2.132062
H	-2.029077	2.279261	3.488094
H	-0.932902	3.273014	2.493524
H	-3.222189	1.38142	-2.407987
H	-2.810224	3.072172	-2.691922
H	-4.526294	2.552661	-2.791789
H	1.013587	2.277695	-2.835398
H	1.195465	4.043783	-2.705585
H	-0.366706	3.335738	-3.232181
H	-0.966669	5.011584	0.776832
H	-1.559244	5.21842	-0.8887
H	0.123848	5.684499	-0.481571
O	-0.470628	0.051641	-2.158611
H	1.302771	-0.411051	-2.957575

Supplementary Table 9. Cartesian geometry of TS2 (10.9 kcal/mol) in Figure 5 in Angstrom [Å].

Atomtype	X Coordinates	Y Coordinates	Z Coordinates
C	-4.764045	-1.180177	0.249188
C	-3.613469	-1.645421	-0.421954
C	-3.770091	-2.260392	-1.684426
C	-5.045084	-2.429442	-2.245623
C	-6.185056	-1.980372	-1.560803
C	-6.037136	-1.34713	-0.317068
P	-1.91382	-1.443497	0.321499
C	-2.252099	-1.507543	2.131535

C	-2.564197	-0.287123	2.770967
C	-2.861346	-0.25642	4.142304
C	-2.837284	-1.444349	4.892837
C	-2.527695	-2.661859	4.262504
C	-2.243744	-2.696189	2.887428
C	-1.122722	-3.064804	-0.127947
Si	0.724655	-3.520271	0.252875
C	1.000035	-5.192944	-0.596639
N	1.686036	-2.287648	-0.524292
C	2.714431	-2.50334	-1.46996
C	2.495382	-2.219386	-2.853514
C	3.552789	-2.369148	-3.771367
C	4.820903	-2.804529	-3.356755
C	5.035901	-3.096066	-2.001508
C	4.00422	-2.951049	-1.052418
C	1.131456	-1.766214	-3.322325
C	4.266058	-3.214911	0.412861
C	0.980658	-3.638417	2.12298
Be	1.162472	-0.842116	-0.071903
O	1.029065	-0.396071	1.417482
Be	-0.784763	0.594807	-0.065191
N	-1.481488	1.994802	-0.587299
C	-2.7851	2.035819	-1.106191
C	-3.794457	2.557473	-0.240825
C	-5.130384	2.606639	-0.680746
C	-5.491076	2.131282	-1.949254
C	-4.507974	1.564844	-2.775709
C	-3.162971	1.496031	-2.368213
C	-3.447235	3.002257	1.166328
C	-2.121065	0.814444	-3.224659
Si	-0.502649	3.42987	-0.750871
C	0.412509	3.44952	-2.416162
C	0.674477	3.39685	0.826794
P	1.838266	1.990035	0.722216

C	3.119709	2.323108	-0.531382
C	4.0518	1.293869	-0.827033
C	4.983832	1.466609	-1.860583
C	5.012881	2.66589	-2.592797
C	4.129549	3.707513	-2.263616
C	3.185553	3.541285	-1.237473
C	2.74555	1.779478	2.305151
C	3.196437	0.4833	2.649286
C	3.892399	0.287688	3.853522
C	4.133547	1.36788	4.719231
C	3.677814	2.654653	4.381004
C	2.989469	2.863431	3.175942
C	-1.969281	4.614118	-0.313406
H	0.370356	0.048805	-0.947614
H	-2.569495	0.640096	2.186878
H	-3.058351	-1.422007	5.967652
H	-2.008707	-3.654019	2.41234
H	-1.135187	-3.074776	-1.234115
H	-1.808906	-3.874669	0.190411
H	6.027107	-3.432218	-1.665338
H	-3.104594	0.699002	4.624759
H	3.372181	-2.14792	-4.832839
H	-4.673528	-0.685155	1.222174
H	-2.506822	-3.5926	4.843947
H	-5.143675	-2.911458	-3.226966
H	-2.898475	-2.60811	-2.250398
H	-7.182037	-2.110501	-2.00056
H	5.633849	-2.919695	-4.085764
H	-6.917757	-0.971676	0.219037
H	4.033415	0.353176	-0.266275
H	5.72639	2.784225	-3.417442
H	2.504251	4.360857	-0.999412
H	0.066331	3.185498	1.726392
H	1.224712	4.337589	1.024529

H	-4.791184	1.143377	-3.749712
H	5.673039	0.65323	-2.111272
H	-5.900163	3.011136	-0.007615
H	2.641863	3.87185	2.916907
H	4.162377	4.655205	-2.81586
H	4.242957	-0.718143	4.118058
H	2.99128	-0.358695	1.978483
H	4.674602	1.207738	5.660604
H	-6.536718	2.168534	-2.280529
H	3.861563	3.499608	5.056983
H	2.024985	-3.916846	2.355453
H	0.312103	-4.384855	2.59274
H	0.781425	-2.645232	2.564334
H	5.34884	-3.260207	0.629434
H	3.823714	-4.175532	0.743722
H	3.79606	-2.43203	1.03758
H	1.080725	-1.703823	-4.424079
H	0.864203	-0.778808	-2.902894
H	0.342966	-2.459208	-2.972045
H	2.050246	-5.518553	-0.473897
H	0.805234	-5.112519	-1.682863
H	0.344301	-5.980887	-0.179324
H	-2.360514	2.925826	1.353842
H	-4.002642	2.402821	1.915466
H	-3.752668	4.051441	1.336034
H	-1.27296	1.482126	-3.457368
H	-2.550968	0.446698	-4.173004
H	-1.675432	-0.048364	-2.691716
H	0.853243	2.462559	-2.640169
H	1.202189	4.22025	-2.494164
H	-0.339146	3.674482	-3.197831
H	-2.426017	4.445645	0.667964
H	-2.788692	4.507722	-1.056325
H	-1.620262	5.666047	-0.344544

H	-0.253068	0.981496	2.384377
C	0.241398	0.794365	1.413444

Supplementary Table 10. Cartesian geometry of IM2 (-13.8 kcal/mol) in Figure 5 in Angstrom [Å].

Atomtype	X Coordinates	Y Coordinates	Z Coordinates
C	2.868509	3.012425	2.296045
C	2.529902	3.556426	1.035375
C	2.918492	4.872358	0.708129
C	3.641529	5.640349	1.635397
C	3.986057	5.096347	2.885044
C	3.600249	3.784168	3.212404
P	1.569097	2.490459	-0.109465
C	2.819191	1.599736	-1.102869
C	3.672226	0.67899	-0.443828
C	4.563548	-0.108876	-1.189139
C	4.629457	0.024963	-2.585767
C	3.826586	0.976996	-3.235148
C	2.92565	1.76386	-2.498963
C	0.526259	1.270835	0.792449
O	1.318605	0.193375	1.259923
C	0.521838	3.521785	-1.182177
Si	-0.852778	2.669107	-2.281348
C	-2.025879	4.09146	-2.735077
N	-1.590141	1.394203	-1.355216
C	-2.946564	1.064959	-1.629697
C	-4.016956	1.836079	-1.082827
C	-5.347493	1.513356	-1.417104
C	-5.646336	0.42961	-2.249994
C	-4.60016	-0.36481	-2.741588
C	-3.259147	-0.068632	-2.442818
C	-3.771206	2.970766	-0.109098
C	-2.148449	-0.963234	-2.941194
C	-0.121212	2.047485	-3.922951

Be	1.348973	-0.859888	0.112544
N	1.851583	-2.368453	0.315393
C	2.81414	-3.003265	-0.499296
C	2.503584	-3.349658	-1.849829
C	3.500265	-3.910751	-2.671129
C	4.795375	-4.149135	-2.185508
C	5.099621	-3.822691	-0.855526
C	4.130773	-3.252825	-0.006179
C	1.109282	-3.113991	-2.383364
C	4.488802	-2.854617	1.407726
Si	0.956676	-3.103255	1.621946
C	1.344598	-2.383714	3.328299
C	-0.917551	-2.819806	1.20944
P	-1.69666	-1.151973	0.946817
C	-1.907592	-0.401738	2.619025
C	-2.194002	0.98018	2.676355
C	-2.398038	1.616674	3.910622
C	-2.305084	0.880872	5.104389
C	-2.020312	-0.494503	5.055007
C	-1.829934	-1.135258	3.819523
C	-3.44493	-1.640228	0.508049
C	-3.684395	-2.75488	-0.326758
C	-4.994227	-3.142908	-0.646663
C	-6.08863	-2.420792	-0.146412
C	-5.860363	-1.297896	0.663516
C	-4.551814	-0.90949	0.989281
C	1.186039	-4.984948	1.590293
Be	-0.606995	0.478215	-0.390446
H	0.478896	-0.431876	-1.004277
H	-2.253135	1.554726	1.745575
H	-2.453412	1.377906	6.071738
H	-1.613933	-2.208221	3.800572
H	-1.010849	-3.320202	0.227305
H	-1.568425	-3.388253	1.903065

H	6.112464	-4.000078	-0.466633
H	-2.622948	2.690742	3.938402
H	3.250184	-4.172562	-3.708937
H	-4.399409	-0.031916	1.626916
H	-1.946125	-1.074456	5.984
H	-5.156269	-4.013518	-1.295343
H	-2.851184	-3.330812	-0.744504
H	-7.113213	-2.722808	-0.398047
H	5.56028	-4.59026	-2.837997
H	-6.705901	-0.711133	1.044021
H	3.622635	0.563077	0.643285
H	5.308745	-0.614495	-3.162302
H	2.304582	2.494106	-3.023433
H	-0.030643	4.181849	-0.484531
H	1.160724	4.180329	-1.801017
H	-4.82692	-1.242477	-3.361415
H	5.190328	-0.848291	-0.680408
H	-6.161945	2.117649	-0.992446
H	2.657136	5.302805	-0.267149
H	3.888201	1.103044	-4.323505
H	3.864896	3.360287	4.189472
H	2.551978	1.991373	2.548256
H	4.553476	5.697985	3.606599
H	-6.688381	0.184574	-2.491715
H	3.938413	6.665834	1.380496
H	2.404916	-2.55414	3.590244
H	0.717185	-2.827642	4.124767
H	1.168509	-1.293522	3.295392
H	5.58364	-2.826056	1.55625
H	4.074404	-3.562701	2.152554
H	4.056602	-1.86686	1.653675
H	0.984495	-3.536989	-3.396169
H	0.865615	-2.036349	-2.421227
H	0.349926	-3.565258	-1.716763

H	2.245296	-5.245818	1.774101
H	0.913829	-5.391512	0.597935
H	0.568827	-5.489307	2.35828
H	-2.69747	3.078942	0.1139
H	-4.307073	2.787556	0.843181
H	-4.140052	3.937053	-0.503104
H	-1.398825	-0.405356	-3.528881
H	-2.540599	-1.788837	-3.560808
H	-1.584914	-1.406765	-2.097948
H	0.50479	1.148904	-3.780921
H	0.470572	2.816627	-4.457833
H	-0.968623	1.768774	-4.579272
H	-2.288516	4.718312	-1.864689
H	-2.965574	3.681519	-3.150833
H	-1.564525	4.739574	-3.505182
H	0.089127	1.862929	1.62682

Supplementary Table 11. Cartesian geometry of TS3 (2.4 kcal/mol) in Figure 5 in Angstrom [Å].

Atomtype	X Coordinates	Y Coordinates	Z Coordinates
C	-4.472618	-1.2336	0.983707
C	-3.285415	-1.837028	0.52118
C	-3.384256	-2.890013	-0.419976
C	-4.635451	-3.344191	-0.862101
C	-5.81183	-2.751148	-0.378151
C	-5.722518	-1.690918	0.536997
P	-1.599725	-1.268934	1.054566
C	-1.875823	-0.647212	2.769471
C	-2.281824	0.701003	2.869959
C	-2.554674	1.27856	4.118576
C	-2.408438	0.514604	5.289106
C	-1.999853	-0.827733	5.199522
C	-1.741649	-1.409679	3.94703
C	-0.663965	-2.876704	1.257797

Si	1.247066	-3.051889	1.629694
C	1.584125	-4.913831	1.553037
N	2.069129	-2.278572	0.272228
C	3.032781	-2.931198	-0.541202
C	2.694739	-3.679428	-1.726225
C	3.741973	-4.263303	-2.469958
C	5.08658	-4.147044	-2.090174
C	5.40595	-3.453543	-0.917497
C	4.397784	-2.860607	-0.132277
C	1.26613	-3.929712	-2.2136
C	4.763701	-2.100132	1.122848
C	1.639397	-2.320476	3.323286
Be	1.436412	-0.776288	0.085073
O	1.34998	0.221028	1.248228
C	0.485954	1.292625	0.834213
P	1.425863	2.632926	-0.024012
C	0.26815	3.699069	-0.940207
Si	-1.108176	2.911173	-2.076826
C	-0.361828	2.600419	-3.804799
C	2.36974	3.662958	1.160138
C	2.804523	3.05246	2.357994
C	3.53055	3.804826	3.294676
C	3.814707	5.159789	3.048076
C	3.374511	5.768092	1.859492
C	2.656145	5.021832	0.912576
C	2.673572	1.912893	-1.152135
C	3.608803	0.984359	-0.633504
C	4.501896	0.329121	-1.49671
C	4.486869	0.605803	-2.874414
C	3.600015	1.567938	-3.383994
C	2.697924	2.221161	-2.528621
C	-2.404258	4.273386	-2.342219
N	-1.736274	1.49873	-1.271294
C	-3.07527	1.119833	-1.554679

C	-4.173775	1.614437	-0.796742
C	-5.476358	1.169781	-1.105862
C	-5.707457	0.23361	-2.12074
C	-4.625862	-0.247869	-2.875453
C	-3.323635	0.215061	-2.630985
C	-3.978255	2.558533	0.369308
C	-2.152479	-0.136985	-3.509499
Be	-0.671844	0.514578	-0.327343
H	0.544081	-0.327363	-1.012457
H	-2.380821	1.294704	1.955514
H	-2.611445	0.964174	6.269302
H	-1.432492	-2.459055	3.895124
H	-0.72881	-3.369419	0.269519
H	-1.268609	-3.497747	1.946373
H	6.454158	-3.365209	-0.599098
H	-2.875314	2.327112	4.174933
H	3.485717	-4.835375	-3.372753
H	-4.429611	-0.402006	1.695064
H	-1.884462	-1.428722	6.110672
H	-4.687179	-4.159958	-1.595339
H	-2.481928	-3.358073	-0.832671
H	-6.791852	-3.100768	-0.727438
H	5.875803	-4.612903	-2.69491
H	-6.634624	-1.201754	0.900599
H	3.62059	0.757563	0.4368
H	5.168891	0.070643	-3.547024
H	2.014556	2.963021	-2.946819
H	-0.272775	4.248668	-0.144673
H	0.837988	4.452662	-1.516918
H	-4.799929	-0.957734	-3.695954
H	5.194617	-0.418052	-1.095864
H	-6.319854	1.547081	-0.510399
H	2.319799	5.501695	-0.015393
H	3.596809	1.806921	-4.455187

H	3.870652	3.33112	4.223927
H	2.567401	1.996663	2.545909
H	4.378233	5.74517	3.785437
H	-6.727011	-0.111988	-2.334384
H	3.592497	6.826445	1.667451
H	2.713052	-2.43069	3.563401
H	1.051434	-2.807193	4.123606
H	1.398186	-1.242682	3.298148
H	5.844451	-1.872299	1.157557
H	4.519368	-2.681183	2.033711
H	4.185536	-1.162704	1.201367
H	1.260987	-4.672482	-3.033471
H	0.761133	-3.011943	-2.53584
H	0.606311	-4.29717	-1.396461
H	2.66275	-5.11415	1.698188
H	1.306161	-5.313399	0.559878
H	1.021349	-5.46945	2.326744
H	-2.908404	2.77345	0.526531
H	-4.391101	2.127143	1.301207
H	-4.500604	3.521525	0.20107
H	-1.700441	0.767664	-3.942005
H	-2.428403	-0.817571	-4.333754
H	-1.342808	-0.565626	-2.942513
H	0.344232	1.752971	-3.816383
H	0.148055	3.49576	-4.215517
H	-1.189682	2.355483	-4.496245
H	-2.697226	4.772639	-1.4002
H	-3.315606	3.829221	-2.785461
H	-2.02337	5.042822	-3.04185
H	0.042783	1.814385	1.709482
C	0.388309	-1.520894	-1.306112
O	-0.828985	-0.858475	-1.11115

Supplementary Table 12. Cartesian geometry of IM3 (-10.0 kcal/mol) in Figure 5 in Angstrom [Å].

Atomtype	X Coordinates	Y Coordinates	Z Coordinates
C	-2.866539	-3.378721	-0.906088
C	-2.919463	-2.445449	0.153866
C	-4.158672	-2.198141	0.780137
C	-5.313571	-2.878567	0.36414
C	-5.251259	-3.811511	-0.68229
C	-4.023345	-4.055864	-1.320016
P	-1.359863	-1.561392	0.658062
C	-1.801264	-0.945013	2.340321
C	-2.345833	0.352939	2.431051
C	-2.753416	0.872757	3.669687
C	-2.618613	0.099018	4.834717
C	-2.086606	-1.199589	4.750881
C	-1.686583	-1.722549	3.510539
C	-0.220264	-3.010812	0.943284
Si	1.603635	-2.88765	1.605825
C	2.166969	-4.682428	1.862425
N	2.570409	-2.105385	0.385155
C	3.661188	-2.63993	-0.33332
C	3.480596	-3.488559	-1.466577
C	4.60738	-3.928648	-2.190439
C	5.90535	-3.548835	-1.819671
C	6.086119	-2.712494	-0.706753
C	4.985333	-2.253337	0.040226
C	2.091136	-3.899574	-1.897164
C	5.178408	-1.361524	1.245798
C	1.666945	-1.966035	3.256132
Be	1.957189	-0.686413	-0.070638
O	1.386867	0.313096	0.969042
C	0.339268	1.156536	0.550212
C	1.393273	-0.494103	-1.751701
O	0.181193	-0.183875	-1.956941
Be	-0.813916	0.388973	-0.644308

N	-2.107274	1.334623	-1.124208
C	-3.456509	0.884191	-1.190755
C	-4.471881	1.449865	-0.357634
C	-5.793154	0.960857	-0.437746
C	-6.141646	-0.060738	-1.326958
C	-5.151922	-0.61164	-2.153861
C	-3.822773	-0.160436	-2.100751
C	-4.19653	2.581854	0.613093
C	-2.784705	-0.76823	-3.013145
Si	-1.649543	2.78575	-1.972735
C	-0.987171	2.469882	-3.726652
C	-0.271377	3.709486	-0.933217
P	1.021418	2.73908	-0.094895
C	2.415925	2.376271	-1.216929
C	3.549493	1.735763	-0.658746
C	4.580399	1.276773	-1.49175
C	4.508286	1.484833	-2.880516
C	3.406382	2.15876	-3.432656
C	2.356607	2.596363	-2.607282
C	1.757119	3.748805	1.251916
C	1.906005	5.147393	1.137407
C	2.453507	5.883609	2.200285
C	2.861043	5.227764	3.375364
C	2.715815	3.834105	3.489752
C	2.161901	3.08962	2.435501
C	-3.044836	4.060144	-2.143718
H	-2.452019	0.948372	1.519814
H	-2.929404	0.504611	5.806211
H	-1.282373	-2.73907	3.465228
H	-0.107847	-3.477281	-0.052818
H	-0.790784	-3.743083	1.549012
H	7.100099	-2.411508	-0.407133
H	-3.173665	1.885277	3.720883
H	4.458579	-4.578019	-3.064665

H	-4.230849	-1.464034	1.589681
H	-1.983118	-1.811184	5.656498
H	-3.963487	-4.776817	-2.145751
H	-1.919993	-3.580545	-1.423483
H	-6.156851	-4.339474	-1.007482
H	6.771127	-3.900531	-2.395781
H	-6.270364	-2.664992	0.856508
H	3.606332	1.572728	0.422652
H	5.312306	1.118945	-3.531187
H	1.490562	3.090268	-3.055634
H	-0.812526	4.221372	-0.113758
H	0.208087	4.500957	-1.540864
H	-5.411011	-1.419601	-2.850378
H	5.431651	0.74101	-1.05539
H	-6.559264	1.402174	0.215914
H	1.592669	5.666074	0.221993
H	3.350207	2.330996	-4.514832
H	3.031623	3.32117	4.40709
H	2.045627	2.000532	2.51589
H	3.290746	5.805248	4.203894
H	-7.173759	-0.431329	-1.373465
H	2.563201	6.971996	2.110453
H	2.720369	-1.888096	3.587119
H	1.090195	-2.472352	4.05262
H	1.274336	-0.943924	3.116263
H	6.234134	-1.056915	1.363434
H	4.858795	-1.872913	2.174672
H	4.545106	-0.457106	1.181851
H	2.095433	-4.350839	-2.905229
H	1.401033	-3.035943	-1.898958
H	1.650847	-4.640786	-1.202014
H	3.230891	-4.69586	2.16845
H	2.080961	-5.285996	0.940526
H	1.577281	-5.178384	2.657372

H	-3.121197	2.814623	0.676509
H	-4.560003	2.328423	1.626934
H	-4.72335	3.50539	0.305157
H	-2.292686	-0.003628	-3.638893
H	-3.230825	-1.534658	-3.670283
H	-1.96919	-1.244021	-2.442604
H	-0.263388	1.636547	-3.739752
H	-0.525216	3.371644	-4.176129
H	-1.838932	2.183634	-4.373614
H	-3.240681	4.603658	-1.202833
H	-3.980878	3.549012	-2.438336
H	-2.796141	4.800383	-2.928261
H	1.948954	-0.695729	-2.710001
H	-0.240589	1.523025	1.430089

Supplementary Table 13. Cartesian geometry of TS4 (-3.1 kcal/mol) in Figure 5 in Angstrom [Å].

Atomtype	X Coordinates	Y Coordinates	Z Coordinates
C	2.662008	2.866193	2.56334
C	2.267759	3.578228	1.408925
C	2.781945	4.868363	1.160975
C	3.689238	5.444412	2.063219
C	4.091909	4.732608	3.207461
C	3.577048	3.448563	3.45617
P	1.196745	2.723343	0.188103
C	2.446722	2.136082	-1.026485
C	3.514752	1.33323	-0.55281
C	4.4336	0.776118	-1.454451
C	4.321539	1.038384	-2.830983
C	3.296266	1.874153	-3.300551
C	2.358998	2.417908	-2.404997
C	0.259628	1.277234	0.909364
O	1.175879	0.26808	1.258987
C	0.066456	3.909324	-0.616356

Si	-1.446656	3.259449	-1.683887
C	-2.6856	4.694007	-1.72699
N	-2.066651	1.83955	-0.899264
C	-3.459677	1.554421	-0.927444
C	-4.270154	2.002124	0.159843
C	-5.646647	1.709823	0.168452
C	-6.23289	0.969485	-0.870911
C	-5.437149	0.52947	-1.93697
C	-4.058195	0.820435	-1.992076
C	-3.622165	2.743235	1.306863
C	-3.248038	0.384634	-3.193267
C	-0.888162	2.980421	-3.483445
Be	1.646286	-0.728868	0.142858
N	2.343438	-2.154265	0.423077
C	3.242482	-2.68213	-0.535346
C	2.795431	-3.35105	-1.716706
C	3.736205	-3.766814	-2.681349
C	5.1104	-3.557787	-2.49867
C	5.556519	-2.930104	-1.325823
C	4.645895	-2.492137	-0.345065
C	1.329907	-3.654229	-1.926637
C	5.138276	-1.830119	0.921472
Si	1.609196	-3.047991	1.732008
C	1.842912	-2.125856	3.36555
C	-0.270239	-3.236619	1.308324
P	-1.353086	-1.771981	0.808086
C	-2.292765	-1.401953	2.351039
C	-3.457966	-0.609629	2.240534
C	-4.135701	-0.175646	3.387622
C	-3.662728	-0.522766	4.665786
C	-2.504708	-1.307429	4.785353
C	-1.820475	-1.742922	3.636721
C	-2.604735	-2.649718	-0.251858
C	-3.629771	-3.461874	0.280451

C	-4.512064	-4.142219	-0.573681
C	-4.375364	-4.02755	-1.968947
C	-3.357186	-3.222632	-2.506057
C	-2.481212	-2.530085	-1.653241
C	2.316062	-4.810375	1.801066
Be	-0.915019	0.78761	-0.388149
O	-0.077251	0.089068	-1.716602
C	1.082162	-0.389225	-1.5209
H	-3.833725	-0.317091	1.251679
H	-4.192222	-0.177949	5.563245
H	-0.908594	-2.336915	3.753523
H	-0.258996	-3.859593	0.39486
H	-0.831667	-3.827834	2.060023
H	6.63283	-2.773083	-1.165878
H	-5.032073	0.446341	3.276358
H	3.378204	-4.271957	-3.589691
H	-1.709014	-1.878531	-2.079052
H	-2.122944	-1.580593	5.777835
H	-5.309948	-4.765991	-0.1494
H	-3.742027	-3.552396	1.368654
H	-5.06495	-4.562397	-2.635052
H	5.827931	-3.888923	-3.260951
H	-3.249266	-3.123242	-3.593891
H	3.609556	1.124913	0.518198
H	5.033781	0.587214	-3.533138
H	1.554925	3.045342	-2.793636
H	-0.39365	4.466806	0.222835
H	0.668314	4.637509	-1.193685
H	-5.888337	-0.05365	-2.75194
H	5.228249	0.120724	-1.084047
H	-6.262673	2.057094	1.009602
H	2.488694	5.419181	0.257626
H	3.208305	2.093476	-4.372255
H	3.894424	2.891139	4.346457

H	2.275047	1.852648	2.73328
H	4.812801	5.179576	3.903715
H	-7.304755	0.734089	-0.846719
H	4.091957	6.446217	1.86637
H	2.922824	-1.962603	3.544995
H	1.426297	-2.671427	4.234613
H	1.358869	-1.136172	3.279774
H	6.185326	-1.489019	0.823345
H	5.091097	-2.530736	1.779309
H	4.493317	-0.976749	1.196851
H	1.112536	-3.911414	-2.978526
H	0.682413	-2.811551	-1.627885
H	1.011509	-4.514356	-1.304693
H	3.277044	-4.82872	2.350208
H	2.510275	-5.185081	0.777978
H	1.624133	-5.514356	2.301647
H	-2.802117	2.140722	1.740107
H	-4.34997	2.968866	2.106234
H	-3.165181	3.693583	0.974725
H	-3.323998	1.133868	-4.007375
H	-3.623568	-0.571337	-3.598531
H	-2.178711	0.281927	-2.951005
H	-0.328094	2.03553	-3.593577
H	-0.281894	3.818217	-3.882341
H	-1.79213	2.903036	-4.116385
H	-2.8249	5.17365	-0.741304
H	-3.67014	4.309766	-2.055875
H	-2.363604	5.468464	-2.449555
H	1.616549	-0.622	-2.482174
H	-0.25325	1.737852	1.824281

Supplementary Table 14. Cartesian geometry of **11’** in Figure 5 in Angstrom [Å].

Atomtype	X Coordinates	Y Coordinates	Z Coordinates

P	1.945959	-2.35921	0.046756
Si	-0.611083	-4.068192	-0.928688
O	1.474027	0.165418	-0.585417
N	-1.53894	-2.744961	-0.307777
C	-2.896349	-2.795164	0.100199
C	3.659217	-2.360263	-0.603204
C	-3.238979	-3.183126	1.427697
C	2.128766	-1.902117	1.818388
C	-3.920555	-2.346855	-0.784845
C	1.16426	-4.012871	-0.107411
H	1.077112	-4.422196	0.916185
H	1.8371	-4.681818	-0.674392
C	-0.330042	-3.991542	-2.809634
C	0.898439	-1.098928	-0.757865
H	0.953346	-1.418763	-1.83105
C	1.090948	-1.224357	2.494701
H	0.16339	-0.952399	1.978573
C	-3.54513	-1.818654	-2.150202
C	-5.261266	-2.351789	-0.353506
H	-6.045267	-2.007634	-1.042483
C	-1.347429	-5.766356	-0.524545
C	4.276365	-1.09295	-0.764574
H	3.731104	-0.17681	-0.507753
C	-2.144941	-3.535075	2.409576
C	-4.590528	-3.176246	1.824655
H	-4.847868	-3.478113	2.849628
C	4.366937	-3.531027	-0.95142
H	3.914731	-4.521637	-0.818789
C	1.251885	-0.861889	3.84148
H	0.443451	-0.324152	4.352455
C	-5.603888	-2.775619	0.940131
H	-6.653818	-2.780431	1.261881
C	3.473571	-1.848725	3.854538
H	4.407237	-2.087591	4.37939

C	5.672753	-3.438956	-1.462202
H	6.217525	-4.354404	-1.726897
C	3.322975	-2.209161	2.506169
H	4.14534	-2.712751	1.982995
C	2.439474	-1.172627	4.524172
H	2.563226	-0.881206	5.57501
C	6.279069	-2.181883	-1.627901
H	7.299905	-2.114474	-2.025977
C	5.578354	-1.013266	-1.280476
H	6.034993	-0.02339	-1.402658
Be	-0.807998	-1.308396	-0.186315
P	-1.946135	2.358798	-0.045816
Si	0.610556	4.069752	0.927336
O	-1.473119	-0.165128	0.588573
N	1.538515	2.746477	0.306801
C	2.896038	2.796189	-0.100921
C	-3.659634	2.359239	0.603514
C	3.23919	3.184875	-1.42808
C	-2.128066	1.900935	-1.817361
C	3.919819	2.346585	0.784007
C	-1.165478	4.013031	0.107828
H	-1.079812	4.422675	-0.915763
H	-1.838283	4.681215	0.675767
C	0.331463	3.994374	2.808645
C	-0.898076	1.099561	0.759862
H	-0.95314	1.420411	1.832775
C	-1.089256	1.224344	-2.493357
H	-0.161397	0.953651	-1.977086
C	3.543904	1.81756	2.148913
C	5.26061	2.350887	0.352921
H	6.044233	2.005761	1.041842
C	1.344949	5.768416	0.521373
C	-4.27673	1.091811	0.764275
H	-3.731414	0.175746	0.507331

C	2.145716	3.538788	-2.409902
C	4.590811	3.17725	-1.824783
H	4.84857	3.479531	-2.849533
C	-4.367599	3.529825	0.95182
H	-3.915394	4.520505	0.819655
C	-1.249446	0.86139	-3.840094
H	-0.440235	0.324557	-4.350785
C	5.603743	2.775278	-0.940395
H	6.653718	2.779506	-1.262016
C	-3.472349	1.84546	-3.85379
H	-4.406195	2.083034	-4.378906
C	-5.673571	3.43751	1.462146
H	-6.218497	4.352836	1.726946
C	-3.322492	2.206367	-2.505472
H	-4.14553	2.709116	-1.982558
C	-2.437285	1.170508	-4.52308
H	-2.560499	0.878678	-5.573865
C	-6.279846	2.180339	1.627237
H	-7.300834	2.11274	2.024894
C	-5.578913	1.011893	1.27969
H	-6.035549	0.021949	1.401291
Be	0.808179	1.30936	0.187739
H	0.036346	2.980296	3.135361
H	-0.448708	4.707031	3.141908
H	1.26986	4.252656	3.33677
H	1.297118	6.005594	-0.556793
H	2.411124	5.785193	0.818861
H	0.822806	6.571058	1.076723
H	1.373938	2.747526	-2.425954
H	2.545063	3.666195	-3.432064
H	1.62272	4.474026	-2.134024
H	2.969181	2.559082	2.733739
H	4.434689	1.522781	2.731394
H	2.888365	0.930701	2.054145

H	-1.268002	-4.248677	-3.339064
H	0.450129	-4.704271	-3.142723
H	-0.033937	-2.977324	-3.135025
H	-2.41229	-5.782775	-0.826772
H	-1.304668	-6.002605	0.554032
H	-0.823324	-6.569742	-1.076945
H	-4.436108	-1.523483	-2.732193
H	-2.97147	-2.560974	-2.735104
H	-2.888749	-0.932326	-2.056356
H	-2.544565	-3.666507	3.431119
H	-1.376556	-2.740542	2.428231
H	-1.617663	-4.467341	2.131829

5. Supplementary references

1. P. M. Keil, T. Szilvási, and T. J. Hadlington, Reversible metathesis of ammonia in an acyclic germylene–Ni0 complex, *Chem. Sci.* **2021**, *12*, 5582–5590.
2. J. T. Patton, M. M. Bokota and K. A. Abboud, Indium-Bridged Chelating Diamide Group IV Metal Olefin Polymerization Catalysts, *Organometallics* **2002**, *21*, 10, 2145–2148.
3. A. Paparo, and C. Jones, Beryllium Halide Complexes Incorporating Neutral or Anionic Ligands: Potential Precursors for Beryllium Chemistry, *Chem. Asian J.* **2019**, *14*, 486–490.
4. P. J. Bailey, R. A. Coxall, C. M. Dick, S. Fabre, L. C. Henderson, C. Herber, S. T. Liddle, D. Loroño-González, A. Parkin, and S. Parsons, The First Structural Characterisation of a Group 2 Metal Alkylperoxide Complex: Comments on the Cleavage of Dioxygen by Magnesium Alkyl Complexes, *Chem. Eur. J.* **2003**, *9*, 4820-4828.
5. K. Dehnicke, B. Neumüller, Neues aus der Chemie des Berylliums, *Z. Anorg. Allg. Chem.* **2008**, *634*, 2703.
6. D. Naglav, M. R. Buchner, G. Bendt, F. Kraus, S. Schulz, Off the Beaten Track—A Hitchhiker's Guide to Beryllium Chemistry, *Angew. Chem. Int. Ed.* **2016**, *55*, 10562.
7 . F. Eisenträger, A. Göthlich, I. Gruber, H. Heiss, C. A. Kiener, C. Krüger, J. U. Notheis, F. Rominger, G. Scherhag, M. Schultz, B. F. Straub, M. A. O. Volland, P. Hofmann, UV-photoelectron spectroscopy and a convenient general synthesis of $^{t}Bu_2PCH_2P^{t}Bu_2$ and related species, *New J. Chem.*, **2003**, *27*, 540–550.
8 . L. Turculet, J. D. Feldman, and T. D. Tilley, Coordinatively and Electronically Unsaturated Zwitterionic Iron Silyl Complexes Featuring the Tripodal Phosphine Ligand $[PhB(CH_2P^{i}Pr_2)_3]^-$, *Organometallics*, **2003**, *22*, 4627-4629.
9. G. M. Sheldrick, SHELXL-97, Program for Crystal Strucutre Refinement, Göttingen, 1997.
10. G. Sheldrick, Crystal structure refinement with SHELXL, *Acta Crystallogr., Sect. C: Struct. Chem.*, **2015**, *71*, 3-8.
11. *WinXPOW,* 3.0.2.1, STOE & Cie GmbH, Darmstadt, Germany, **2011**.
12. (a) A. D. Becke, Density-functional thermochemistry. V. Systematic optimization of exchange-correlation functionals, *J. Chem. Phys*., **1997**, *107*, 8554-8560; (b) F. Weigend, R. Ahlrichs, Balanced basis sets of split valence, triple zeta valence and quadruple zeta valence quality for H to Rn: Design and assessment of accuracy, *Phys. Chem. Chem. Phys.*, **2005**, *7*, 3297-3305; (c) J.-D. Chai, M. Head-Gordon, Long-range corrected hybrid density functionals with damped atom–atom dispersion corrections, *Phys. Chem. Chem. Phys.*, **2008**, *10*, 6615-6620; (d) S. Grimme, J. Antony, S. Ehrlich, H. Krieg, A consistent and accurate ab initio parametrization of density functional dispersion correction (DFT-D) for the 94 elements H-Pu, *J. Chem. Phys*., **2010**, *132*, 154104; (e) A. V. Marenich, C. J. Cramer, D. G. Truhlar, Universal Solvation Model

Based on Solute Electron Density and on a Continuum Model of the Solvent Defined by the Bulk Dielectric Constant and Atomic Surface Tensions, *J. Phys. Chem. B*, **2009**, *113*, 6378-6396.

13. Gaussian 16, Revision A. 03, M. J. Frisch, G. W. Trucks, H. B. Schlegel, G. E. Scuseria, M. A. Robb, J. R. Cheeseman, G. Scalmani, V. Barone, G. A. Petersson, H. Nakatsuji, X. Li, M. Caricato, A. V. Marenich, J. Bloino, B. G. Janesko, R. Gomperts, B. Mennucci, H. P. Hratchian, J. V. Ortiz, A. F. Izmaylov, J. L. Sonnenberg, D. Williams-Young, F. Ding, F. Lipparini, F. Egidi, J. Goings, B. Peng, A. Petrone, T. Henderson, D. Ranasinghe, V. G. Zakrzewski, J. Gao, N. Rega, G. Zheng, W. Liang, M. Hada, M. Ehara, K. Toyota, R. Fukuda, J. Hasegawa, M. Ishida, T. Nakajima, Y. Honda, O. Kitao, H. Nakai, T. Vreven, K. Throssell, J. A. Montgomery, Jr., J. E. Peralta, F. Ogliaro, M. J. Bearpark, J. J. Heyd, E. N. Brothers, K. N. Kudin, V. N. Staroverov, T. A. Keith, R. Kobayashi, J. Normand, K. Raghavachari, A. P. Rendell, J. C. Burant, S. S. Iyengar, J. Tomasi, M. Cossi, J. M. Millam, M. Klene, C. Adamo, R. Cammi, J. W. Ochterski, R. L. Martin, K. Morokuma, O. Farkas, J. B. Foresman, and D. J. Fox, Gaussian, Inc., Wallingford CT, 2016.
14. T. Lu, F. Chen, Multiwfn: A multifunctional wavefunction analyzer, *J. Comput. Chem.*, **2012**, *33*, 580-592.
